# Mass-Spectrometry-Based Lipidome and Proteome Profiling of *Hottentotta saulcyi* (Scorpiones: Buthidae) Venom

**DOI:** 10.3390/toxins14060370

**Published:** 2022-05-26

**Authors:** Parviz Ghezellou, Kevin Jakob, Javad Atashi, Alireza Ghassempour, Bernhard Spengler

**Affiliations:** 1 Institute of Inorganic and Analytical Chemistry, Justus Liebig University Giessen, 35392 Giessen, Germany; kevin.jakob@chemie.uni-giessen.de; 2 Medicinal Plants and Drugs Research Institute, Shahid Beheshti University, Tehran 1983969411, Iran; avandkasheftasnim@gmail.com (J.A.); a-ghassempour@sbu.ac.ir (A.G.)

**Keywords:** scorpion, venom, proteomics, lipidomics, *Hottentotta saulcyi*, Iran

## Abstract

Scorpion venom is a complex secretory mixture of components with potential biological and physiological properties that attracted many researchers due to promising applications from clinical and pharmacological perspectives. In this study, we investigated the venom of the Iranian scorpion *Hottentotta saulcyi* (Simon, 1880) by applying mass-spectrometry-based proteomic and lipidomic approaches to assess the diversity of components present in the venom. The data revealed that the venom’s proteome composition is largely dominated by Na^+^- and K^+^-channel-impairing toxic peptides, following the enzymatic and non-enzymatic protein families, e.g., angiotensin-converting enzyme, serine protease, metalloprotease, hyaluronidase, carboxypeptidase, and cysteine-rich secretory peptide. Furthermore, lipids comprise ~1.2% of the dry weight of the crude venom. Phospholipids, ether-phospholipids, oxidized-phospholipids, triacylglycerol, cardiolipins, very-long-chain sphingomyelins, and ceramides were the most intensely detected lipid species in the scorpion venom, may acting either independently or synergistically during the envenomation alongside proteins and peptides. The results provide detailed information on the chemical makeup of the venom, helping to improve our understanding of biological molecules present in it, leading to a better insight of the medical significance of the venom, and improving the medical care of patients suffering from scorpion accidents in the relevant regions such as Iran, Iraq, Turkey, and Afghanistan.

## 1. Introduction

Scorpions represent a venomous group of arachnids distributed on all of the continents except Antarctica. The number of discovered scorpions is approximately 2567 species as of writing this paper, classified into 23 families (https://www.ntnu.no/ub/scorpion-files/, accessed on 1 June 2021). Among them, only a few species—mostly from the Buthidae family, the largest and widespread of the families, as well as the Scorpionidae and Hemiscorpiidae families—are responsible for the pathological manifestations induced by scorpion stings to humans [1,2]. In general, the clinical observations of scorpion envenomation and severity of symptoms are directly correlated to the concentration of injected venom, the kind of scorpion species, and the victim’s age. The common symptoms start with local pain, sweat, febricula, nausea, vomiting, feeling faint and rapid alternating cycles of hyper- and hypotension. However, the severe form of scorpion envenomation can be fatal—especially in children and elderly individuals—by progressing to cardiovascular collapse associated with respiratory complications (e.g., bronchospasm and pulmonary edema) and myocardial failure [3]. Given that scorpion envenomation incidence exceeds 1.2 million individuals per year, resulting in more than 3250 deaths, scorpionism is a life-threatening emergency and remains a severe health problem in several countries [1,2,3,4].

Scorpion venom is a complex secretory mixture that contains water, salts, lipids, amino acids, mucopolysaccharides, nucleotides, peptides, proteins, and some other unknown compounds [5,6,7]. Evidence from previous studies revealed that the biological activities of peptides cause the toxic properties of scorpion venoms, which target membrane-bound protein channels and receptors specifically [5]. In general, the peptides of scorpion venoms can be categorized into two main groups: disulfide-bridged peptides (DBPs), which are specifically acting on membrane ionic channels (Na^+^, K^+^, Ca^2+^, and Cl^−^) [8,9], and non-disulfide bridged peptides (NDBPs) showing different activities, such as antimicrobial, bradykinin-potentiating, hemolytic, and immunomodulation [10,11] activity. Alternatively, biologically active molecules present in the venoms of various scorpion species have been shown to cover a wide range of therapeutic modalities from antibacterial to anticancer [12]. These compounds are ideal candidates for new-drug developments due to their high specificity and potency for specific molecular targets [13]. By way of example, chlorotoxin (CTX) isolated from the venom of a deathstalker scorpion (*Leiurus quinquestriatus*) binds preferentially to the human glioma and medulloblastoma cells, allowing to the diagnosis of different types of cancer. By using CTX, Olson et al., recently developed a bioconjugate as a “Tumor Paint” product for effective imaging of a wide variety of tumors without affecting healthy tissue [14]. Therefore, scorpions have attracted many researchers due to potential applications from chemical and pharmacological as well as clinical and pathological perspectives.

Over the last decade, evaluation and estimation of the toxin composition of scorpion venoms have been enhanced by technological improvements. The development of high-resolution mass spectrometry (HRMS) combined with transcriptomic analysis has played a pivotal role in obtaining a holistic overview of the venom’s complexity. Mass-spectrometry-based molecular mapping is a promising technique for assessing a wide range of molecules present in biological samples. Given the extensive research conducted to identify and characterize the molecular components of venom, we still generally lack a deep understanding of venom components and their functions. So far, 1014 proteins/peptides have been assigned to reviewed entries in the UniProt database from 77 scorpion species, of which 819 of entries indicated a toxin (https://www.uniprot.org/biocuration_project/Toxins, accessed on 1 March 2021). However, more than 200,000 different proteinaceous components are estimated to be expressed in the venom glands of all known scorpion species [2].

In contrast to proteinaceous components, little consideration has been devoted to small metabolite constituents of venoms. Marie and Ibrahim (1976) reported that lipids compose 1.7% of the dry weight of deathstalker scorpion, *Leiurus quinquestriatus*, crude venom [15]. They also revealed that lipids were not derived by cells shed from the venom glands, and also that delipidated venom did not change its toxicity. Recently, LC-MS analysis of venoms from several snake species has shown that their venoms generally contain a wide variety of metabolites which may induce certain biological activities during snakebite independently or synergistically [16,17]. Like some proteinic components of scorpion venoms, small metabolites have recently shown beneficial characteristics similar to bactericidal activities [18]. Even so, metabolite information regarding animal venoms, e.g., scorpion and spider, is primarily unexplored and more extensive research is required.

The genus *Hottentotta* is one of the most broadly spread genera of the family Buthidae, with its species found across Asia and Africa [19]. The clinical effects of stings by the representatives of the genus are described as suffering local effects such as severe pain and swelling without significant systemic symptoms [20,21]. However, despite the high envenomation rate [20,21], sufficient clinical and venom-compositional information is still not available. The venom gland transcriptional profiles of just two species of the genus, *H. judaicus* and *H. conspersus*, have been investigated so far [22,23]. These studies revealed the existence of transcripts annotating to sodium-, potassium-, chloride-, and calcium-channel toxins as well as bradykinin-potentiating peptides, among others, in the venom gland of the *Hottentotta* genus. However, there are not any proteomics data confirming at least their expression in the secreted venom yet.

In this study, we employed liquid chromatography tandem mass spectrometry (LC-MS/MS)-based proteomic and lipidomic approaches to explore the molecular diversity of the yet unstudied venom of *H. saulcyi*. With a median lethal dose (LD50) value (intravenous administration) of 0.73 mg/kg (Turkey) [24], 1.07 mg/kg (Iraq) [25], and 1.01 mg/kg (Iran) [26] in the laboratory mice (20 ± 2 g), the *H. saulcyi* venom seems to be one of the family’s most toxic species, and accidents with the species can be dangerous to humans in the distributed area including Iran, Iraq, Syria, Turkey and Afghanistan. Despite the close similarities in LD50 value, intraspecies venom variation has been reported in scorpion specimens [27,28,29], which can result from, e.g., evolutionary history, climatic factors, ontogeny, or adaptation toward different prey. Thus, knowledge of venom composition among *H. saulcyi* populations geographically can provide critical information for predicting clinical symptoms and the likely efficacy of an existing antivenom and for influencing the design of more effective immunizing mixtures for future antivenom production.

## 2. Results

### 2.1. Mass Fingerprint of Crude Venom

**Figure 1:**
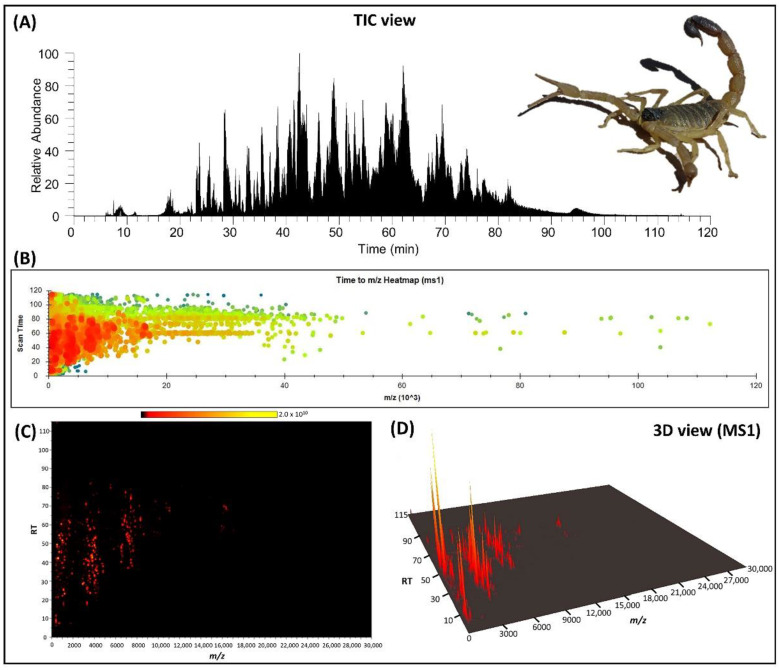
Mass profiling of *Hottentotta saulcyi* venom. (**A**) Total ion chromatogram (TIC) of the soluble venom on the C18 column under a ACN/H_2_O gradient elution for 120 min (using Xcalibur software); (**B**) heatmap of MS1 spectra, based on decolvoluted accurate mass (*m*/*z*) and scan time without intensity filtering; and (**C**) 2D visualization of MS1 from mass range below 30 kDa (using SeeMS software). (**D**) MS1 feature in 3D plot, limited to mass range <30 kDa (using Mass++ software).

Analysis of the whole soluble venom, using liquid chromatography–Orbitrap mass spectrometry, resulted in the elution profile of venom peaks as shown in the TIC chromatogram in Figure 1A. The deconvoluted spectrum revealed the molecular mass distribution in the range of 350 Da to 112 kDa, as shown as a heatmap in Figure 1B. After processing the spectrum manually by removing the peaks with intensities lower than 1E5 as well as dimers or doubly charged species, oxidation, deamination, hydration, and sodium adducts, a mass list was created along with an estimate of the number of peptides contained in the venom. A total of 203 individual signals, ranging from 360 to 40,855 Da, were detected as shown in Table 1. The molecular mass profile is distinctly tri-modal, with the most abundant components falling within the ranges of ≤1800 Da, 2900–4000 Da, and 6500–7700 Da (Figure 1C,D). Although there are no data regarding the components of *H. saulcyi* venom, the mass categories indicate that the species, as a member of the Buthidae scorpion, follows mostly the expected venom composition trends mentioned by previous studies [5,12]. The largest cluster refers to the low molecular masses (≤1800 Da), which are relatively unknown structurally and functionally. In contrast, the molecular mass ranges of 2900–4000 Da and 6500–7700 Da are well known and specified in the categories of K^+^ and Na^+^ channel toxins, respectively.

Furthermore, the majority of high-mass components are grouped in the range of about 10–11 and 16 kDa and eluted in the retention time of 70 to 80 min (Figure 1C,D; Table 1) due to their hydrophobic features [10,11]. In general, proteins with molecular mass higher than 9 kDa comprise a minor part of scorpion venom components [5], which is also revealed here in the venom of *H. saulcyi*. Not much is known about these proteins, and only few of them appear to be enzymes according to their homology with protein families such as hyaluronidase and phospholipase A2 [10]. However, in order to validate and match directly the listed molecular masses with protein families, species-specific venom gland transcriptomic analysis is required.

### 2.2. Peptidomics

Peptide spectra recorded by top-down peptidomics of crude venom were de novo sequenced, generating high-quality sequence tags using the PEAKS software (https://www.bioinfor.com/Peaks-studio, accessed on 1 March 2021). A table including all sequence tags with average local confidence (ALC) of more than 50% is supplied in the Supporting Information. The obtained sequences were manually searched against the arachnid and animal toxins databases using BLAST (https://blast.ncbi.nlm.nih.gov/Blast.cgi, accessed on 1 March 2021) to identify the homologs of proteins. The output results indicated that most of the obtained sequence tags did not have any match in the databases employed. The main reasons for this are the lack of species-specific databases and sequence variations. The latter is driven by different mutations that also comprise the principal parts of *Hottentotta* toxin transcripts [23]. The result has led to identification of polypeptides present in the venom; they belong to the five known families previously discovered in the scorpion venom of other species, which include Na^+^ and K^+^ channel toxins, bradykinin-potentiating peptide, orcokinin, and elongation factor (Table 2).

### 2.3. Protein Identification Using In-Gel and In-Solution Tryptic Digestion followed by LC-MS/MS

**Figure 2:**
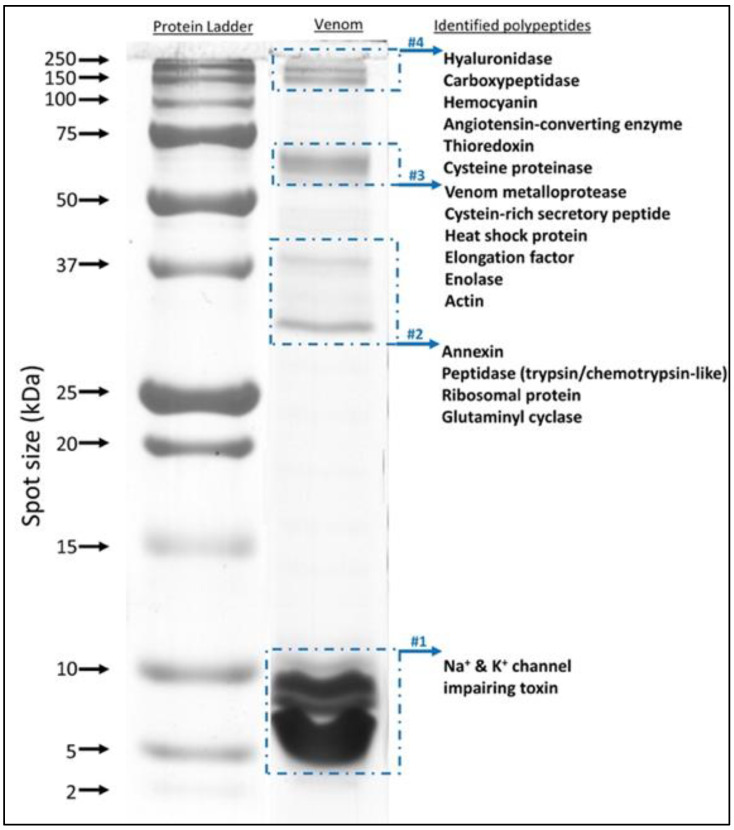
1D SDS-PAGE of *Hottentotta saulcyi* crude venom and protein identification of the bands; 20 µg of the crude venom solution was loaded onto a lane of 12.5% polyacrylamide gel and stained with Coomassie blue after electrophoresis. The four selected regions (marked by blue dashed rectangles) were cut, followed by tryptic digestion and LC-MS/MS analysis. The spectral data were searched for within the UniProtKB database (https://www.uniprot.org/, accessed on 1 March 2021), limited to the taxonomy Arachnida (ID#6854). The identified polypeptides of each band are indicated below the arrow.

SDS-PAGE of the whole soluble venom was used for further investigation of the composition and complexity of *H. saulcyi* venom. As expected from Figure 1, the separated pattern uncovered the distribution of proteins at different molecular weights ranging from 3 to 150 kDa (Figure 2). The more intense bands were clearly observed in the low molecular mass range (less than 10 kDa), which occupied about 62% of the protein spots in the venom lane of the gel. In the next step, the whole lane was cut into the four bands, followed by in-gel digestion with trypsin, and the extracted peptides were analyzed using LC-MS/MS for protein identification. Through sequence database searching using tandem mass spectral data, proteins were identified from all separated bands (Supplementary Materials, Table S2). Several major toxin groups were identified, including Na+ and K+ channel modulators, venom metalloprotease, cysteine-rich secretory peptides, hyaluronidase, and peptidase-like proteins, as well as numerous cellular processing proteins such as hemocyanin, thioredoxin, actin, heat shock protein, annexin, glutaminyl cyclase, enolase, and elongation factor (Figure 2).

To obtain a comprehensive proteome analysis, the unseparated venom was also digested directly in solution, and resulting peptides were analyzed by LC-MS/MS. Interpretation of tandem mass spectra against the UniProtKB database restricted by taxonomy to Arachnida (a total of 729,678 sequences) resulted in the identification of peptides derived from proteins related to venom and cellular or biological processes. The cumulative total of identified proteins in both approaches (in-gel and in-solution digestion) was 200, filtering proteins that had at least two unique peptides together with less than 1% FDR (Table S1, Supplementary Materials). Toxin categories of identified proteins in both techniques are presented in Table 3. Based on the results of SDS-PAGE and mass fingerprint analyses, highly abundant protein entities were identified as toxins, modulating the ion channel functions, including the K^+^-channel-impairing toxins (with 18 sequence entries) and Na^+^-channel-impairing toxins (with 11 sequence entries). In addition to enzymes as mentioned above for gel electrophoresis, several venom components were determined from tryptic peptides. However, this was limited to a minor extent, such as for angiotensin-converting enzyme and protease inhibitor.

### 2.4. The Scorpion Venom Lipidome

Lipids account for about 1.2% (m/m) of *H. saulcyi* venom composition (839 µg/70 mg of the crude venom). In total, 399 unique lipids across 30 different lipid classes in positive ion mode and 317 unique lipids across 26 different lipid classes in negative ion mode were identified from the extracted lipid fraction of the venom (Figure 3). For more detailed information, all identified lipid species are listed in Supplementary Materials Tables S3 and S4. Additionally, the matching fragment ion series obtained from tandem mass spectra are graphically depicted in Supplementary Materials Figures S1–S39 for representative lipids.

**Figure 3:**
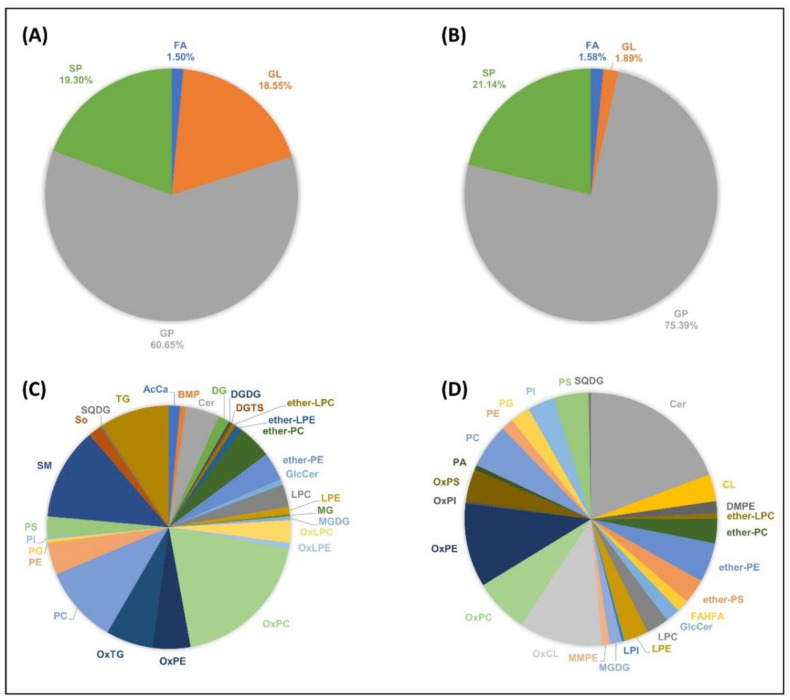
Scorpion venom lipidome. Relative abundance of the identified lipid categories detected in (**A**) positive and (**B**) negative ion mode as well as lipid types detected in (**C**) positive and (**D**) negative ion mode in the venom of *H. saulcyi*. AcCa: acylcarnitine; BMP: bis(monoacylglycero)phosphate; Cer: ceramide; CL: cardiolipin; CoQ: coenzyme Q; DG: diacylglycerol; DGDG: digalactosyldiacylglycerol; DGTS: diacylglyceryltrimethylhomoserine; DMPE: dimethylphosphatidylethanolamine; ether-: prefix for ether-linked lipids; FA: fatty acyls; FAHFA: fatty acid ester of hydroxyl fatty acid; GL: glycerolipids; GlcCer: glucosylceramides; GP: glycerophospholipids; HBMP: hemibismonoacylglycerophosphate; LDGTS: lysodiacylglyceryltrimethylhomoserine; LPA: lysophosphatidic acid; LPC: lysophosphatidylcholine; LPE: lysophosphatidylethanolamine; LPI: lysophosphatidylinositol; MG: monoacylglycerol; MGDG: monogalactosyldiacylglycerol; MMPE: monomethyl-phosphatidylethanolamine; Ox: prefix for oxidized lipids; PA: phosphatidic acid; PC: phosphatidylcholine; PE: phosphatidylethanolamine; PEtOH: phosphatidylethanol; PG: phosphatidylglycerol; PI: phosphatidylinositol; PK: polyketides; PMeOH: phosphatidylmethanol; PR: prenol lipids; PS: phosphatidylserine; SL: saccharolipids; SM: sphingomyelin; So: sphingosine; SP: sphingolipids; SQDG: sulfoquinovosyldiacylglycerol; ST: sterol lipids; TG: triacylglycerol.

All identified lipid species belonged to the four lipid categories (Figure 3A,B), namely: fatty acyls (FAs), glycerolipids (GLs), glycerophospholipids (GPs), and sphingolipids (SPs). Among them, GPs were the most abundant venom lipids, detected in both ion polarities. The absolute number of identified lipids in the lipid categories was 242 GPs (60.6%), 77 SPs (19.3%), 74 GLs (18.5%), and 6 FAs (1.5%) in positive ion mode, and 239 GPs (75.4%), 67 SPs (21.1%), 6 GLs (1.9%), and 4 FAs (1.5%) in negative ion mode. In addition, the major lipid types identified on the *H. saulcyi* venom in positive ion mode included protonated oxidized phosphatidylcholines (OxPCs), sphingomyelins (SMs), phosphatidylcholines (PCs), and ammoniated triacylglycerols (TGs). The distribution of the 30 different lipid types in positive ion mode is shown in Figure 3C. In negative ion mode, the major lipid types were found to be deprotonated and formate adducts of ceramides (Cers), [M − 2H]^2−^ species of oxidized cardiolipins (OxCLs), deprotonated oxidized phosphatidylethanolamines (OxPEs), and formate adducts of OxPCs (Figure 3D). The results show that oxidized species were mostly detected in negative ion mode rather than in positive ion mode. High levels of oxidized lipids might be a consequence of the milking method, in which the extracted venom is exposed to the open air for seconds before deep freezing.

## 3. Discussion

### 3.1. The Proteome of H. saulcyi Venom

The data from mass spectrometric fingerprinting, gel electrophoresis, and bottom-up proteomics revealed that the proteome composition of Iranian *H. salucyi* venom is dominated by toxins with molecular masses less than 10 kDa, assumed to modulate K^+^ and Na^+^ channel activities. These findings were also supported by our peptidomics results, which discovered molecular weights of 7071.1187, 7312.2598, 7462.3755, and 7074.1265 Da in the crude venom homologated to NaTxs (long-chain toxins) and two fragmented peptides, 1817.9668 and 2146.1191 Da belonging to the KTxs (short-chain toxins). The present outcomes also correlate well with previous reports on proteome analyses of scorpion venoms in which Na+- and K+-channel-impairing toxins comprise the major toxin components of Buthidae scorpion venoms [30,31].

The primary differentiation between the scorpion venom of Buthidae and non-Buthidae families is the predominant presence of NaTxs in the venom of the Buthidae family. They are responsible for the neurotoxic nature of scorpion venoms and play a leading role in the medical consequences of scorpionism [9]. NaTxs are classified into two families, α-NaTx and β-NaTx, according to their physiological effects on the voltage-gated sodium channel [32]. While α-NaTxs delay or inhibit the channel’s normal inactivation process, β-NaTxs encourage the channel opening at more negative membrane potentials [11,22]. Interestingly, studies of transcripts of the venom glands of two species of the genus *Hottentotta* have shown that the number of sequences encoding NaTxs is much lower than for other members of the Buthidae family [19,23]. Furthermore, α-NaTx transcripts in the venom glands of both species were found to be under-represented compared to β-NaTx. However, the proteomics results of *H. saulcyi* venom here confirmed α-NaTx only in the venom. It is noteworthy that variations between the expressed proteome in scorpion venom and the venom gland transcription profile have been reported [33].

The majority of peptides identified in the *H. saulcyi* venom belongs to the toxins acting on potassium channels (KTx). KTx is another important group of scorpion venom peptides which inhibit K+ channel activities. Based on their amino acid sequences, length, and structure, KTxs can be categorized into seven families, including α-, β-, γ-, σ-, ԑ-, κ-, and λ-KTxs, with α-KTx being the largest, containing 31 subfamilies. MS/MS database search of *H. saulcyi* digested venom indicated that most KTx-annotated peptides are similar to sequences found in transcriptomes of scorpion species of *Mesobuthus eupeus* and *Androctonus bicolor* (Table 3). Results suggests that two subfamilies of KTx, α-KTx and β-KTx, exist in the venom of *H. saulcyi*. Although information regarding their venom toxicity functions is limited, they can be suggested to play an essential role in insect-hunting [23].

Regarding toxins that target other channels, sequence information coding for chloride (ClTx) and calcium (CaTx) channel toxins was found in the venom gland transcriptomes of the *Hottentotta* genus [22,23]. However, we did not detect any of them in the venom proteome of *H. saulcyi*. Moreover, studies on several scorpion species have reported enzymatic components to be found in the scorpions’ venoms [31]. In addition to playing an essential role in cellular metabolic processes, these components can also act as toxins in animal venoms (e.g., snakes and spiders), engaging in the envenomation process considerably [30,34,35]. We also identified peptide sequences corresponding to enzymes such as serine proteases, metalloproteases, hyaluronidases, carboxypeptidase, and angiotensin-converting enzyme (Table 3). Although a high abundance of protease transcripts has been reported in the *H. judaicus* venom gland [23], they are expressed in only minor quantities in the venom. Serine proteases are known for their hydrolytic activity: cleaving peptide amide bonds. Their fibrinolytic activities are also reported in scorpion, *Tityus bahiensis*, *T. serrulatus,* and *T. discrepans* venoms except metalloproteases [36,37]. A recent report indicated an interesting novel target for snake serine protease acting as a potassium channel blocker [38]. Hyaluronidases are known as spreading factors which degrade the hyaluronan of the interstitial matrix and facilitate toxin diffusion [39]. Angiotensin-converting enzymes (ACEs) are also detected in other scorpion species, *Tityus bahiensis*, *T. stigmurus*, *T. serrulatus,* and *H. judaicus*. It is considered that ACEs contribute to envenomation symptoms by releasing angiotensin II and causing hypertension [40]. Other proteinaceous toxins belonging to cysteine-rich secretory proteins (CRISPs) and protease inhibitors were detected. They have also been found in the venom of other animals, showing various functions. Recent studies have revealed that CRISPs as non-enzymatic proteins can regulate a range of ion channels [41,42] and mediate inflammatory responses [43].

Along with the proteinaceous constituents pointed above, we also detected small molecules (<1 kDa) by mass profiling the crude venom. These molecules accounted for a considerable part of the non-proteinaceous components of the venom. Small molecules are often reported within scorpion venoms belonging to, e.g., lipids, free amino acids, nucleotides, and amines [44]. However, the efficiency of structure identification, mainly because of a lack of database, is much lower than for peptides/proteins [45]. Nevertheless, a few non-peptide small molecules of scorpion venoms have been characterized confidently, such as 1,4-benzoquinone derivatives, adenosine, adenosine monophosphate, and citric acid [18,44]. They can play essential toxic or non-toxic roles, and future research should aim to explore their structure and functions in more detail. In the following, we describe the identification of lipids within *H. saulcyi* venom, and more extensive work is still needed to identify and characterize the rest of the metabolite.

### 3.2. The Lipidome of H. saulcyi Venom

A broad diversity of lipid species was detected in the venom of *H. saulcyi*. GPs and SPs are the most abundant lipid components of the scorpion venom. They were also reported as the most plentiful lipid parts of Palestine yellow scorpion, *L. quinquestriatus*, venom [15]. Phospholipids and ether-phospholipids comprise most of the glycerophospholipids identified here, which—as bioactive lipid mediators and platelet-activating factors (PAFs)—may be involved in diverse physiological and pathological processes, e.g., inflammation, allergy, and apoptosis, as response to envenomation [17,46,47]. Additionally, the scorpion venoms, like snake venoms [17], contain a wide range of sphingomyelin (SM) and ceramide (Cer) species. Apart from the primary function of sphingolipids in maintaining cell membrane function and integrity, some species are bioactive, and a variety of cellular processes are attributed to them, including cell survival and growth, immune-cell trafficking, cell differentiation, and autophagy [48]. One of the most interesting features concerning the sphingolipid composition of the *H. saulcyi* venom is the presence of very-long-chain Cer and SM species, such as SM (d20:1/26:0), SM (d20:2/24:0), SM (d18:1/24:0), Cer (d20:2/20:0), Cer (d28:1/h20:0), and Cer (d18:1/24:0). A recent investigation revealed that exogenously added very-long-chain SMs, e.g., SM (d18:1/24:0), can activate mouse macrophages, resulting in inflammatory responses, whereas short-chain SM (d18:1/6:0) did not [49]. In addition, ceramides also showed apoptotic activities on HeLa and lung cancer cell lines [49,50]. Another interesting point that can be mentioned is the presence of a large diversity of cardiolipin (CL) and triacylglycerol (TG) species in the venom. CLs are unique phospholipids carrying two negatively charged phosphate groups in their polar head as well as four hydrophobic acyl chains. They play an essential role in energy metabolism, apoptosis, mitophagy, and signaling to promote inflammation [51,52]. TGs contain a glycerol backbone with three esterified FAs. They are the essential source of stored energy in the animal, playing an important role in energy homeostasis [53]. Recent studies have shown that the metabolic functions of TGs are not limited to energy storage but also serve as signaling molecules, for example to enhance the inflammatory function of macrophages [53] or to cope with a broad spectrum of abiotic stresses [54].

The production and storage of venom are metabolically expensive, especially for scorpions adapting to survive in extremely arid and semi-arid environments on limited resources for at least 400 million years [55]. Scorpions learned to control and regulate venom injection wisely to minimize metabolic expenses. As a result, it is conceivable that scorpions, besides peptides, also used lipids to increase the range of biological activities of their venoms to overcome prey or defend themselves. However, to assign the function of these molecules in the scorpion venoms, one must have a deep understanding of the pathogenesis of reactions induced by scorpion envenomation, requiring future research.

## 4. Conclusions

Now, analytical methods play a key role in the identification and characterization of diverse biomolecular species in animal venoms. Here, we have investigated for the first time the proteome and lipidome of *Hottentotta saulcyi* venom using mass-spectrometry-based techniques. The results indicate that scorpion venom compositions are highly heterogenous and complex to a much larger extent than previously thought. Our study revealed that the toxic part of *H. saulcyi* venom is primarily dominated by Na^+^- and K^+^-channel toxins. Moreover, low-molecular-weight non-proteinaceous molecules comprise a considerable portion of the crude venom with lipids as ~1.2% of the dry weight. Despite a confident lipid identification in this study, a comprehensive analysis is still needed to elucidate the other metabolites of venom, which is our future plan. The MS information provided here will be useful in future research on the venom of scorpions and other venomous animals and will provide a better understanding of the complex pathology behind their envenomation.

## 5. Materials and Methods

### 5.1. Samples

Adult scorpions of the species *Hottentotta saulcyi* were collected from the area around Fars Province, Iran. Venom samples were obtained by electrical stimulation of the telson (posterior-most part of the scorpion) followed by immediate flash-freezing with liquid nitrogen. The specimens were released at their capture site and the pooled venom sample was lyophilized and stored at −80 °C for future research.

### 5.2. Chemicals

LC-MS-grade acetonitrile (ACN), water (H_2_O), acetone (ACE), methanol (MeOH), isopropanol (IPA), formic acid (FA), methyl-tert-butyl ether (MTBE), ammonium formate (AF), and bovine serum albumin (BSA) were purchased from Sigma-Aldrich (Steinheim, Germany). RapiGest was purchased from Waters (Waters Corporation, Milford, MA, USA). The mass-spec-grade Trypsin/Lys-C mix was purchased from Promega (Promega, Mannheim, Germany). Zip Tip C18 was purchased from Millipore (Millipore, Bedford, MA, USA). Coomassie Brilliant Blue reagent was purchased from Bio-Rad (Feldkirchen, Germany).

### 5.3. Proteomics Sample Preparation

After venom extraction, the pooled venom was centrifuged at 12,000× *g* at 4 °C for 10 min, and the supernatant was stored at −80 °C until further use. Protein concentration was determined before each proteomics analysis using a standard Bradford protein assay [56], with BSA used as a reference. Absorbance was measured spectrophotometrically at 590 nm on a BioTek Synergy 2 plate reader (BioTek, Winooski, VT, USA) with Gen5 software (version 2.01; https://www.biotek.com/, accessed on 1 March 2021).

The crude venom was separated with 15% tris-glycine sodium dodecyl sulfate polyacrylamide gel electrophoresis (SDS-PAGE, Bio-Rad, Feldkirchen, Germany) according to the Laemmli procedure [57]. SDS-PAGE gel was run without reducing agent and stained with Coomassie Brilliant Blue G-250 (Sigma-Aldrich, Steinheim, Germany). The scanned gel picture was analyzed for relative densities of protein spots using ImageJ software (https://imagej.nih.gov/ij/list.html, accessed on 1 March 2021). Subsequently, the protein bands were cut manually and subjected to in-gel tryptic digestion.

### 5.4. UHPLC-MS/MS Proteomics

For top-down peptidomics, the crude venom was separated using a Jupiter C18 (4.6 × 250 mm, 3 µm 300 A° particle size) column (Phenomenex, Torrance, CA, USA) attached to an UltiMate 3000 RSLC UHPLC system (Thermo Fisher Scientific, Bremen, Germany), and the system was coupled to a Q Exactive HF-X Orbitrap (Thermo Fisher Scientific, Bremen, Germany) mass spectrometer. Chromatographic analysis was performed at a flow rate of 400 µL/min, using water/0.1% FA (mobile phase A) and ACN/0.1% FA (mobile phase B). The gradient of 115 min was applied as follows: isocratically (2% B) for 3 min, 2–55% B over 100 min, and re-equilibration in 2% B. The mass spectrometer was operated in data-dependent acquisition (top-5 DDA) with the following parameters in full MS scans: mass range *m*/*z* 350–2000, mass resolution of 120,000 (@ *m*/*z* 200), AGC target of 1e6, injection time (IT) of 100 ms and MS/MS scans: mass resolution 30,000 (@ *m*/*z* 200), AGC target of 1e5, IT of 120 ms, isolation window *m/z* ±1.3, dynamic exclusion 30 s, and normalized collision energy (NCE) of 28.

The raw files were examined using PEAKS Studio software suite version 8.5 (Bioinformatics Solutions Inc., Waterloo, CA, USA). De-novo-sequenced peptides with average local confidence (ALC) scores ≥50% were selected for database searches against the Arachnida (taxon ID # 6854) from UniProtKB (https://www.uniprot.org/, downloaded on 1 March 2021) without enzyme constraint using a mass tolerance of 10 ppm and mass accuracy of 0.1 Da for the precursor and fragment ions, respectively. A decoy database was set to < 1% in order to calculate the false discovery rate (FDR). Peptide sequences obtained from de novo analysis do not match with any sequence in the database submitted to the homology search by blasting manually against the Uniport/SwissProt databases (https://www.uniprot.org/, accessed on 1 March 2021).

For bottom-up proteomics, the whole venom (20 µg) was diluted with 50 mM ammonium bicarbonate buffer containing 0.1% RapiGest and incubated for 15 min in a thermomixer at 80 °C (Eppendorf Thermomixer C, Hamburg, Germany) to complete proteome solubilization. The venom and SDS-PAGE band samples were reduced and alkylated with 100 mm dithiothreitol (DTT) at 56 °C for 15 min, and 200 mm IAA at room temperature (dark place) for 30 min, respectively. The digestion was performed with mass spec grade Trypsin/Lys-C mix (1:25 enzyme to proteins ratio) at 37 °C. The reaction was stopped after 16 h by adding FA and incubating at 37 °C for 10 min prior to centrifugation. The peptide samples were desalted before the mass measurements by using ZipTip C18 and then concentrated by using Eppendorf Concentrator Plus (Eppendorf, Hamburg, Germany) and finally stored at −80 °C for future use.

The peptides were separated using an UltiMate 3000 RSLC HPLC system (Ultrahigh-Performance Liquid Chromatography, Thermo Fisher Scientific, Bremen, Germany) on a Kinetex C18 (2.1 × 100 mm, 2.6 µm 100 Å particle size) column (Phenomenex, CA, USA) coupled to a Q Exactive HF-X (Thermo Fisher Scientific, Bremen, Germany) mass spectrometer. Chromatographic analysis was performed at 250 µL/min flow rate with water/0.1% formic acid (mobile phase A) and ACN/0.1% formic acid (mobile phase B). The optimized gradient elution of 90 min was applied as follows: isocratically (2% B) for 5 min, followed by 2–40% B over 70 min, 40–50% B over 5 min, 50–98% B over 2 min, and re-equilibration in 2% B. The mass spectrometers were operated in data-dependent acquisition (top-10 DDA) with the following parameters in full MS scans: mass range of *m*/*z* 350 to 1800, resolution of 120,000 (@ *m*/*z* 200), AGC target of 3e6, IT of 50 ms and MS/MS scans: mass range of *m/z* 200 to 2000, mass resolution of 30,000 (@ *m*/*z* 200), AGC target of 1e5, IT of 120 ms, isolation window *m*/*z* 1.3 and dynamic exclusion of 60s.

The raw files were searched against the UniProtKB flat-file database taxonomically set to the Arachnida (taxon ID # 6854) databases (downloaded on 1 March 2020) using Proteome Discoverer software suite, version 2.2 (Thermo Fisher Scientific, Bremen, Germany) with the peptide precursor and fragment ion mass tolerance set to 10 and 0.6 ppm, respectively. The parameters were set to two maximum missed cleavage sites of trypsin digestion, minimum peptide length of 6, MS1 and MS2 tolerances of 10 ppm and 0.5 Da, respectively. The dynamic modification was set to oxidation (+15.995 Da [M]) and static modification to carbamidomethyl (+57.021 Da [C]). Percolator [58] node was used to validate identified peptide spectrum matches (PSMs) and filter the data with parameters of a strict Target FDR (false discovery rate) of 0.01 and a relaxed Target FDR of 0.05. The MaxQuant contaminant database was used to mark contaminants in the results file.

### 5.5. Lipid Extraction and UHPLC-MS/MS Lipidomics

The lipidome of venom was extracted using methyl-tert-butyl ether (MTBE) extraction procedure [59] with some modification. Briefly, 300 µL cold methanol was added to the 70 mg sample and vortexed for 5 min and incubated on ice for 10 min. Afterwards, 1 mL of cold MTBE was added to the solution, vortexed, and sonicated for 5 min with ice. The solution was incubated for 1h on the Thermomixer (Eppendorf Thermomixer C, Hamburg, Germany) at 2 °C with 950 rpm. For the next step, 250 µL of cold water was added, incubated for 5 min, and cold centrifuged (Beckman Coulter, Krefeld, Germany) for 10 min at 12,000 rpm. The upper organic layer, which should contain the lipids, was transferred into a new pre-cooled microtube, and the whole lipid extraction process was repeated again for the rest. The organic layers were then vaporized by a nitrogen flow and the dry lipids weighed using an analytical balance (Mettler Toledo, Gießen, Germany). The lipid samples were finally kept at −80 °C until further use.

The extracted lipids were separated on an analytical column Kinetex C18 (Phenomenex, Torrance, CA, USA) (2.1 × 100 mm, 2.6 µm 100 A° particle size), connected to a Thermo Scientific Dionex UltiMate 3000 UHPLC system. Mobile phase A was a mixture of ACN/H_2_O (60:40), and mobile phase B was a mixture of IPA/ACN/H_2_O (90:8:2). Both mobile phases contained 10 mM ammonium formate and 0.1% formic acid. The flow rate was set to 250 µL/min, and gradient elution started at 20% mobile phase B, rising to 30% B over 4 min; 45% B over 2 min; 60% B over 4 min; 65% B over 4 min and held for another 4 min; 90% B over 13 min, and the column re-equilibrated with 20% B for 10 min prior to the next injection.

A heated electrospray ionization source (HESI II) connected to the Q Exactive HF-X Orbitrap mass spectrometer was used for ionization of the samples in positive and negative ion mode. Ion source settings were tuned as follows: spray voltage of 3.8 kV, source temperature of 325 °C, capillary temperature of 300 °C, sheath gas of 60 (40 for negative polarity), and auxiliary gas of 20 (10 for negative polarity). Each sample was measured in both ion polarities using Data Dependent Acquisition (Top-10) with the following parameter values in full MS scan: mass range *m/z* 200–1200, mass resolution of 60,000 (@ *m*/*z* 200), AGC target of 5e6, IT of 75 ms and MS/MS scans: mass resolution of 60,000 (@ *m*/*z* 200), AGC target of 5e6, IT of 175 ms, isolation window *m*/*z* ±1, dynamic exclusion of 6 s, and stepped NCE levels of 20–25–30.

The raw files were converted to an mzXML format using MSconvert [60] and then further processed by MZmine version 2.3 (http://mzmine.github.io/, accessed on 1 March 2021) [61]. Lipids were identified via LipidMatch R-based software (http://secim.ufl.edu/secim-tools/lipidmatch/, accessed on 1 March 2021) [62] and reported only for compounds which exhibited a precursor ion *m/z* error below 7 ppm and MS/MS peaks corresponding to matching fatty acid chain lengths.

## Figures and Tables

**Table 1:** Mass fingerprint of the *Hottentotta saulcyi* venom. Molecular mass signals with more than 1 × 10^5^ intensity were detected and grouped in 10 min intervals. Molecular masses below 3 kDa were reported as monoisotopic mass values, while those above 3 kDa were reported as average mass values.

RT * (min)	MW ** (Da)
0–10	366.10, 393.01, 419.31, 441.29, 527.15, 610.25, 689.21, 735.04, 830.53, 896.48, 945.58, 1114.58, 1760.88, 1848.55
10–20	360.16, 389.22, 516.24, 741.47, 1121.62, 1210.48, 2200.16, 3276.27, 3357.40, 3588.60, 3904.57, 4059.84, 4186.87
20–30	374.22, 479.29, 534.31, 562.26, 842.39, 1032.63, 1138.61, 1186.50, 1212.58, 2138.07, 2201.16, 2254.55, 2950.13, 3006.15, 3313.41, 3359.40, 3387.41, 3434.58, 3462.50, 3590.60, 3874.56, 3974.65, 4083.90, 4174.96, 6802.07, 7177.19, 8843.36
30–40	429.27, 649.36, 804.47, 872.53, 1036.43, 1114.59, 1361.71, 1872.76, 1899.79, 3189.31, 3283.53, 3422.45, 3487.63, 3453.42, 3501.65, 3633.60, 3771.46, 3908.63, 3965.63, 4002.74, 4130.68, 4261.81, 4297.83, 4684.11, 4773.18, 4819.21, 6165.57, 6947.85, 7099.04, 7255.22, 7312.23, 7481.41, 7927.26, 8004.45, 8333.30
40–50	465.27, 522.29, 594.36, 643.40, 664.34, 709.28, 1162.52, 1176.54, 1300.67, 1349.60, 1998.96, 3189.28, 3411.84, 3445.66, 3544.52, 3738.45, 3797.50, 3824.54, 3853.80, 4050.58, 6488.80, 6536.80, 6563.86, 7308.22, 7668.39
50–60	765.35, 1002.40, 1575.65, 2146.16, 3375.45, 3547.46, 3632.32, 3820.60, 4063.62, 3968.71, 6633.89, 7026.07, 7074.05, 7109.11, 7121.10, 7135.16, 7264.21, 7325.25, 7339.23, 7371.39, 7405.41, 7462.21, 7866.45, 8589.02, 9970.49
60–70	621.37, 939.38, 1325.60, 1616.75, 1685.74, 2708.25, 4397.92, 4408.93, 4568.81, 5787.46, 6781.88, 6847.90, 6852.89, 6907.91, 6917.29, 7414.32, 7423.20, 7428.17, 7588.38, 7718.27, 8238.59, 8282.66, 8294.63, 10,162.66, 11,088.19, 11,737.29, 16,339.35, 16,348.28, 16,476.37, 18,298.81, 30,419.97, 37,947.32, 40,855.16
70–80	1098.77, 1149.31, 1307.75, 1420.94, 3242.44, 3941.52, 5524.45, 5555.44, 6932.92, 7407.20, 9761.33, 10,766.28, 14,957.42, 16,195.19, 24,694.55, 40,034.10
80–90	1331.50, 1576.64, 1864.05, 3617.49, 3676.49, 4128.9358, 5525.40, 5596.94, 11,839.56
90–100	386.98, 415.21, 558.92
100–110	468.30, 2268.11, 3111.71

* Retention time, ** Molecular weight.

**Table 2:** Peptide identification of the *Hottentotta saulcyi* venom by de novo sequencing. Top-down venomics data was processed for de novo sequence in PEAKS software. The highly confident sequences were manually searched in BLAST against a non-redundant sequence database limited to taxonomy Arachnida.

Peptide	*m/z*	z	Mass	Protein Name	Organism	Sequence ID	E-Value *	Description
SLENEVFWDVMKKLDFEGP	761.7128	3	2282.1292	putative toxin, partial	*Hottentotta judaicus*	F1CJ05	1 × 10^−2^	-
WGELDFWDVMKKFFPDLP (−0.98)	1135.0576	2	2268.1150	putative toxin, partial	*Hottentotta judaicus*	F1CJ05	3 × 10^−2^	-
FDEDLNVGFNDFGAPSRSH (+15.99)	1070.4878	2	2138.9292	venom neuropeptide-2	*Mesobuthus eupeus*	E4VP42	2 × 10^−5^	Neuropeptide signaling
DFDELDNVGFNDFGPASGVLQ (−0.98)	1128.0040	2	2254.0066	venom neuropeptide-3	*Mesobuthus eupeus*	E4VP55	7 × 10^−10^	Neuropeptide signaling
RSQPSGCNVGFNDFGPASRGPS (−0.98)	1118.5049	2	2235.0015	venom neuropeptide-2	*Mesobuthus eupeus*	E4VP42	6 × 10^−6^	Neuropeptide signaling
MLLDNVGFNDFGPASRHC	996.9552	2	1991.8982	venom neuropeptide-3	*Mesobuthus eupeus*	E4VP55	2 × 10^−7^	Neuropeptide signaling
QPQDLELDKSGFGGFH	831.3739	2	1660.7480	Putative orcokinin	*Hottentotta judaicus*	E4VP55	3 × 10^−5^	Neuropeptide signaling
DLELDKSGFGGFH	711.3378	2	1420.6624	Putative orcokinin	*Hottentotta judaicus*	F8THJ9	3 × 10^−4^	Neuropeptide signaling
RGGKELMNSLKEKLSEAKE	537.5423	4	2146.1191	U9-buthitoxin-Hja1	*Hottentotta judaicus*	F1CIW9	4 × 10^−9^	K^+^ channel impairing toxin
FAANTVLNGPEEEAAVENF	1011.4805	2	2020.9377	Putative toxin Tx297	*Mesobuthus martensii*	B8XH54	4 × 10^−5^	Bradykinin-potentiating peptide
EPDVLNGLLEEAAVPAAE	918.9564	2	1835.9153	Putative toxin Tx297	*Buthus occitanus israelis*	B8XH54	4 × 10^−2^	Bradykinin-potentiating peptide
PAALNHLNGPEEEAAVPAAE	1000.4883	2	1998.9646	Putative toxin Tx297	*Buthus occitanus israelis*	B8XH54	1 × 10^−5^	Bradykinin-potentiating peptide
HAPLKEKLSNMLETAHA	945.5087	2	1888.9829	Elongation factor	*Leptotrombidium deliense*	A0A443ST94	5 × 10^−2^	GTP-binding protein
KNRELMNSLKEKLSE	455.4981	4	1817.9668	Potassium channel toxin	*Mesobuthus eupeus*	P0CH57	2 × 10^−4^	K^+^ channel impairing toxin
WVPGNYPGVLSY	676.3341	2	1350.6721	Toxin b subunit beta	*Androctonus crassicauda*	P0C2A3	4 × 10^−3^	Na^+^ channel impairing toxin
KKDGYPVDSGNCKYECLKDDYCNDLCLERKADKGYCYWGKVSCYCYGLPDNSPTKTSGKCNPA	1180.5389	6	7071.1187	Alpha-toxin CsE5	*Centruroides sculpturatus*	P46066	2 × 10^−68^	Na^+^ channel impairing toxin
ARDGYIANDRNCVYTCALNPYCDSECKKNGADSGYCQW (+15.99) FGRFGNACW (+15.99) CKNLPDKVPIRIPGECRG	1220.3782	6	7312.2598	MeuNaTxalpha-9	*Mesobuthus eupeus*	D8UWD8	1 × 10^−71^	Na^+^ channel impairing toxin
LKDGYIVDDRNCTYFCGTNAYCNEECVKLKGESGYCQWVGRYGNACWCYKLPDHVRTVQAGRCRS (−0.98)	1245.5728	6	7462.3755	Alpha-toxin Bot11	*Buthus occitanus tunetanus*	P01486	5 × 10^−72^	Na^+^ channel impairing toxin
GRDAYIADSENCTYTCALNPYCNDLCTKNGAKSGYCQW (+15.99) AGRYGNACW (+15.99) CIDLPDKVPIRISGSCR	1012.1568	7	7074.1265	Makatoxin-1	*Mesobuthus martensii*	P56569	1 × 10^−69^	Na^+^ channel impairing toxin

* BLAST expect value.

**Table 3:** Identified polypeptides of *Hottentotta saulcyi* venom by LC-MS/MS analysis of tryptic peptides derived from in-gel- and in-solution-digested proteins. Proteins with 1% FDR and containing at least two unique peptides were considered identified and were listed here. Proteins involved in cellular or biological processes are not shown here (see Supplementary Table S1).

Accession	Description	Organism	Coverage (%)	#Peptides	#Unique	Avg. Mass	-10lgP
**Na^+^- channel toxins**							
P0DJH8	Alpha-toxin Bu1 (α-NaTx)	*Buthacus macrocentrus*	60	6	3	7485	85.69
D5HR49	Neurotoxin 9 (Fragment)	*Androctonus bicolor*	86	6	3	7750	82.98
D5HR55	Neurotoxin 2 (Fragment)	*Hottentotta judaicus*	35	3	2	7421	48.05
F1CJ50	U1-buthitoxin-Hj1b	*Hottentotta judaicus*	14	2	2	10,792	50.24
B8XGY7	Putative alpha toxin Tx405 (α-NaTx)	*Buthus occitanus israelis*	32	4	3	9324	48.05
B8XGY1	Putative alpha toxin Tx93 (α-NaTx)	*Buthus occitanus israelis*	31	4	3	9625	48.05
F0V3W0	Alpha neurotoxin precusor (α-NaTx)	*Hottentotta judaicus*	61	6	4	9312	48.05
Q56TT9	Alpha-insect toxin BjaIT (α-NaTx)	*Hottentotta judaicus*	61	6	4	9270	48.05
F1CJ53	Alpha-insect toxin BjaIT (Fragment) (α-NaTx)	*Hottentotta judaicus*	65	4	3	4367	48.05
Q86SE0	Toxin Aam2	*Androctonus amoreuxi*	29	4	2	9283	112.38
P45668	Neurotoxin-2 (Fragment)	*Hottentotta tamulus*	54	2	2	2686	106.55
**K^+^- channel toxins**							
A0A0K0LC05	Potassium channel blocker AbKTx-2 (β-KTx)	*Androctonus bicolor*	16	2	2	10,307	62.92
A0A0K0LC09	Potassium channel blocker AbKTx-7 (α-KTx)	*Androctonus bicolor*	16	2	2	10,308	62.92
A0A088D9U2	Potassium channel blocker pMeKTx28-2 (β-KTx)	*Mesobuthus eupeus*	16	2	2	10,264	62.92
A0A0K0LC11	Potassium channel blocker AbKTx-3 (α-KTx)	*Androctonus bicolor*	19	2	2	8507	62.92
A0A0K0LCJ0	Potassium channel blocker AbKTx-5 (α-KTx)	*Androctonus bicolor*	16	2	2	10,380	62.92
A0A088DB26	Potassium channel blocker pMeKTx28-3 (β-KTx)	*Mesobuthus eupeus*	16	2	2	10,250	62.92
A0A0K0LC08	Potassium channel blocker AbKTx-4 (α-KTx)	*Androctonus bicolor*	31	2	2	5163	62.92
B8XH36	Putative potassium channel toxin Tx633 (β-KTx)	*Buthus occitanus israelis*	18	2	2	8688	62.92
A0A143MGJ8	Potassium channel toxin meuK28-2 (β-KTx)	*Mesobuthus eupeus*	16	2	2	10,408	62.92
A0A0U4GZ05	Potassium channel toxin KTx3 (β-KTx)	*Odontobuthus doriae*	16	2	2	10,313	62.92
A9XE60	Potassium channel toxin MeuTXK-beta-1 (β-KTx)	*Mesobuthus eupeus*	10	3	3	10,338	46.55
A0A0K0LC02	Potassium channel blocker AbKTx-10 (α-KTx)	*Androctonus bicolor*	10	2	2	10,110	46.55
A9XE59	Potassium channel toxin MeuTXK-beta-2 (β-KTx)	*Mesobuthus eupeus*	10	3	3	10,328	46.55
A0A0K0LBZ4	Potassium channel blocker AbKTx-6 (α-KTx)	*Androctonus bicolor*	10	2	2	10,103	46.55
A0A0K0LCI9	Potassium channel blocker AbKTx-11 (α-KTx)	*Androctonus bicolor*	21	5	5	10,086	46.55
A0A0K0LC06	Potassium channel blocker AbKTx-9 (α-KTx)	*Androctonus bicolor*	10	2	2	10,102	46.55
E4VP56	Putative bifunctional venom peptide-5 (β-KTx)	*Mesobuthus eupeus*	14	3	3	7076	46.55
E4VP14	Putative bi-functional venom peptide (β-KTx)	*Mesobuthus eupeus*	10	3	3	10,338	46.55
**Enzymes**							
P86100	Hyaluronidase-1	*Mesobuthus martensii*	54	32	25	47,433	248.11
A0A0C9RFM5	Hyaluronidase	*Tityus bahiensis*	11	4	3	46,533	85.94
A0A1E1WWG5	Hyaluronidase	*Tityus obscurus*	21	5	4	46,678	94.81
F1CIW6	Hyaluronidase (Fragment)	*Hottentotta judaicus*	52	15	9	20,715	182.73
E4VNZ7	Venom metalloprotease-1	*Mesobuthus eupeus*	35	32	24	44,842	239.74
A0A0U4HEU8	Venom protein VP4	*Odontobuthus doriae*	28	8	2	15,973	157.24
A0A1E1WW02	Putative metalloproteinase (Fragment)	*Tityus obscurus*	6	7	4	41,037	89.28
E4VNZ8	Venom metalloprotease-2 (Fragment)	*Mesobuthus eupeus*	32	29	21	35,602	239.74
F1CIU8	Putative M12B metalloprotease (Fragment)	*Hottentotta judaicus*	16	7	7	38,519	192.13
A0A0U1SF04	Peptidase_M14 domain-containing protein (Fragment)	*Isometrus maculatus*	31	5	2	23,077	119.46
E4VP21	Chymotrypsin-like protease-1	*Mesobuthus eupeus*	26	6	4	29,641	143.14
F1CIY2	Putative transmembranal serine protease (Fragment)	*Hottentotta judaicus*	35	6	4	25,193	181.63
F1CJ26	M12B metalloprotease (Fragment)	*Hottentotta judaicus*	37	12	12	27,664	125.9
A0A2I9LNS6	Acid phosphatase	*Centruroides hentzi*	20	6	6	43,244	91.97
A0A4Y2BUR0	Carboxypeptidase E	*Araneus ventricosus*	5	2	2	50,168	67.35
A0A1S5QN46	Carboxypeptidase E	*Tityus serrulatus*	15	7	4	53,838	141.45
A0A1W7RAV1	Carboxypeptidase	*Hadrurus spadix*	6	2	2	49,002	75.85
A0A1E1WVT7	Angiotensin-converting enzyme	*Tityus obscurus*	8	4	4	72,883	65.33
F1CJ87	Putative angiotensin-converting enzyme (Fragment)	*Hottentotta judaicus*	41	3	3	4575	103.37
F1CJ25	Putative angiotensin-converting enzyme (Fragment)	*Hottentotta judaicus*	22	7	7	30,499	147.9
**Other components**							
A0A2I9LPW9	Venom factor	*Centruroides hentzi*	2	3	3	200,889	68.23
F8THJ4	CRISP3 (Fragment)	*Hottentotta judaicus*	73	29	28	21,201	259.16
T1E6Y3	CAP-Iso-2 (Fragment)	*Isometroides vescus*	8	2	2	44,650	53.16
F1CJ75	Putative cysteine-rich secretory peptide (Fragment)	*Hottentotta judaicus*	40	14	11	23,920	219.13

## Data Availability

All mass spectrometric raw files were deposited to the MassIVE repository (https://massive.ucsd.edu/) with the dataset identifier MSV000089086.

## Supplementary Materials

The following supporting information can be downloaded at: https://www.mdpi.com/article/10.3390/toxins14060370/s1, Figures S1–S39: HCD MS/MS fragmentation patterns of the representative lipids; Tables S1–S2: List of all identified protein groups in *Hottentotta saulcyi* venom using in-solution and in-gel tryptic digestion; Table S3: List of all top ranked identified lipids from *Hottentotta saulcyi* venom in positive-ion mode; Table S4: List of all top ranked identified lipids from *Hottentotta saulcyi* venom in negative-ion mode; Table S5: List of all peptide de novo sequences (XLSX format).
