# Supplementary material for: Mass-Spectrometry-Based Lipidome and Proteome Profiling of Hottentotta saulcyi (Scorpiones: Buthidae) Venom
Source: Toxins (Basel). 2022 May 26;14(6):370. doi: 10.3390/toxins14060370 (PMC9228814; doi:10.3390/toxins14060370)
Supplement: Supplementary file 1 [file toxins-14-00370-s001.zip › toxins-1734694 - Supplementary - Final.pdf]

toxins

MDPI

Supplementary Materials

Mass spectrometry-based lipidome and proteome profiling of scorpion, *Hottentotta saulcyi*, venom

Parviz Ghezellou, Kevin Jakob, Javad Atashi, Alireza Ghassempour and Bernhard Spengler

Table S1. Identified protein families in *Hottentotta saulcyi* venom by in-solution and in-gel tryptic digestion.

Accession #	Description	Organism	Coverage (%)	#Peptides	#Unique	Avg. Mass	-10lgP
		**Na^+- channel toxins**					
P0DJH8	Alpha-toxin Bu1	Buthacus macrocentrus	60	6	3	7485	85.69
D5HR49	Neurotoxin 9 (Fragment)	Androctonus bicolor	86	6	3	7750	82.98
D5HR55	Neurotoxin 2 (Fragment)	Hottentotta judaicus	35	3	2	7421	48.05
F1CJ50	U1-buthitoxin-Hj1b	Hottentotta judaicus	14	2	2	10792	50.24
B8XGY7	Putative alpha toxin Tx405	Buthus occitanus israelis	32	4	3	9324	48.05
B8XGY1	Putative alpha toxin Tx93	Buthus occitanus israelis	31	4	3	9625	48.05
F0V3W0	Alpha neurotoxin precusor	Hottentotta judaicus	61	6	4	9312	48.05
Q56TT9	Alpha-insect toxin BjaIT	Hottentotta judaicus	61	6	4	9270	48.05
F1CJ53	BjaIT (Fragment)	Hottentotta judaicus	65	4	3	4367	48.05
Q86SE0	Toxin Aam2	Androctonus amoreuxi	29	4	2	9283	112.38
P45668	Neurotoxin-2 (Fragment)	Hottentotta tamulus	54	2	2	2686	106.55
		**K^+- channel toxins**					
A0A0K0LC05	Potassium channel blocker AbKTx-2	Androctonus bicolor	16	2	2	10307	62.92
A0A0K0LC09	Potassium channel blocker AbKTx-7	Androctonus bicolor	16	2	2	10308	62.92
A0A088D9U2	Potassium channel blocker pMeKTx28-2	Mesobuthus eupeus	16	2	2	10264	62.92
A0A0K0LC11	Potassium channel blocker AbKTx-3 (Fragment)	Androctonus bicolor	19	2	2	8507	62.92
A0A0K0LCJ0	Potassium channel blocker AbKTx-5	Androctonus bicolor	16	2	2	10380	62.92
A0A088DB26	Potassium channel blocker pMeKTx28-3	Mesobuthus eupeus	16	2	2	10250	62.92
A0A0K0LC08	Potassium channel blocker AbKTx-4 (Fragment)	Androctonus bicolor	31	2	2	5163	62.92

B8XH36	Putative potassium channel toxin Tx633	Buthus occitanus israelis	18	2	2	8688	62.92
A0A143MGJ8	Potassium channel toxin meuK28-2	Mesobuthus eupeus	16	2	2	10408	62.92
A0A0U4GZ05	Potassium channel toxin KTx3	Odontobuthus doriae	16	2	2	10313	62.92
A9XE60	Potassium channel toxin MeuTXK-beta-1	Mesobuthus eupeus	10	3	3	10338	46.55
A0A0K0LC02	Potassium channel blocker AbKTx-10	Androctonus bicolor	10	2	2	10110	46.55
A9XE59	Potassium channel toxin MeuTXK-beta-2	Mesobuthus eupeus	10	3	3	10328	46.55
A0A0K0LBZ4	Potassium channel blocker AbKTx-6	Androctonus bicolor	10	2	2	10103	46.55
A0A0K0LCI9	Potassium channel blocker AbKTx-11	Androctonus bicolor	21	5	5	10086	46.55
A0A0K0LC06	Potassium channel blocker AbKTx-9	Androctonus bicolor	10	2	2	10102	46.55
E4VP56	Putative bifunctional venom peptide-5 (Fragment)	Mesobuthus eupeus	14	3	3	7076	46.55
E4VP14	Putative bi-functional venom peptide	Mesobuthus eupeus	10	3	3	10338	46.55
**Enzymes**							
F8THJ4	CRISP3 (Fragment)	Hottentotta judaicus	73	29	28	21201	259.16
T1E6Y3	CAP-Iso-2 (Fragment)	Isometroides vescus	8	2	2	44650	53.16
F1CJ75	Putative cystein-rich secretory peptide (Fragment)	Hottentotta judaicus	40	14	11	23920	219.13
P86100	Hyaluronidase-1	Mesobuthus martensii	54	32	25	47433	248.11
A0A0C9RFM5	Hyaluronidase	Tityus bahiensis	11	4	3	46533	85.94
A0A1E1WWG5	Hyaluronidase	Tityus obscurus	21	5	4	46678	94.81
F1CIW6	Hyaluronidase (Fragment)	Hottentotta judaicus	52	15	9	20715	182.73
E4VNZ7	Venom metalloprotease-1	Mesobuthus eupeus	35	32	24	44842	239.74
A0A0U4HEU8	Venom protein VP4	Odontobuthus doriae	28	8	2	15973	157.24
A0A1E1WW02	Putative metalloproteinase (Fragment)	Tityus obscurus	6	7	4	41037	89.28
E4VNZ8	Venom metalloprotease-2 (Fragment)	Mesobuthus eupeus	32	29	21	35602	239.74
F1CIU8	Putative M12B metalloprotease (Fragment)	Hottentotta judaicus	16	7	7	38519	192.13
A0A0U1SF04	Peptidase_M14 domain-containing protein (Fragment)	Isometrus maculatus	31	5	2	23077	119.46
E4VP21	Chymotrypsin-like protease-1	Mesobuthus eupeus	26	6	4	29641	143.14
F1CIY2	Putative transmembranal serine protease (Fragment)	Hottentotta judaicus	35	6	4	25193	181.63

F1CJ26	M12B metalloprotease (Fragment)	Hottentotta judaicus	37	12	12	27664	125.9
A0A2I9LNS6	Acid phosphatase	Centruroides hentzi	20	6	6	43244	91.97
A0A4Y2BUR0	Carboxypeptidase E	Araneus ventricosus	5	2	2	50168	67.35
A0A1S5QN46	Carboxypeptidase E	Tityus serrulatus	15	7	4	53838	141.45
A0A1W7RAV1	Carboxypeptidase	Hadrurus spadix	6	2	2	49002	75.85
A0A1E1WVT7	Angiotensin-converting enzyme	Tityus obscurus	8	4	4	72883	65.33
F1CJ87	Putative angiotensin-converting enzyme (Fragment)	Hottentotta judaicus	41	3	3	4575	103.37
F1CJ25	Putative angiotensin converting enzyme (Fragment)	Hottentotta judaicus	22	7	7	30499	147.9
**Protease Inhibitors**							
A0A2I9LPW9	Venom factor	Centruroides hentzi	2	3	3	200889	68.23
**Cellular processes' proteins**							
A0A0K8RMV8	Annexin (Fragment)	Ixodes ricinus	15	2	2	17503	81.91
A0A3S3REF1	Actin-like protein (Fragment)	Dinothrombium tinctorium	52	18	2	42515	191.93
A0A146CJA5	Venom toxin meuVNP2	Mesobuthus eupeus	22	4	4	19494	141.68
E4VP42	Venom neuropeptide-2	Mesobuthus eupeus	24	4	4	17948	141.68
E4VP55	Venom neuropeptide-3	Mesobuthus eupeus	22	4	4	19473	141.68
F8THJ9	Putative orcokinin	Hottentotta judaicus	22	4	4	19512	141.68
F1CJ05	Putative toxin (Fragment)	Hottentotta judaicus	49	9	9	4122	119.88
F8THK0	Putative RNA-binding protein (Fragment)	Hottentotta judaicus	21	15	15	54278	180.8
A0A3G5AP15	L Chain L Crystal Structure Of Fab Dx-2930	Tetranychus truncatus	63	9	9	18149	180.32
A0A0K0LC99	Cellular protein AbCp-54 (Fragment)	Androctonus bicolor	43	12	4	10213	169.67
F1CIY0	Hypothetical secreted protein (Fragment)	Hottentotta judaicus	20	9	9	25552	144.14
A0A1E1WVQ8	Putative peptidylglycine alpha-amidating monooxygenase	Tityus obscurus	36	11	8	39271	123.55
P80476	Hemocyanin AA6 chain	Androctonus australis	19	11	3	71786	116.28
A0A1E1WVS7	Putative hemocyanin subunit	Tityus obscurus	12	6	4	72760	113.75
F1CIY3	Hypothetical secreted protein (Fragment)	Hottentotta judaicus	22	3	3	12153	112.32
F1CJ13	Thioredoxin domain-containing protein (Fragment)	Hottentotta judaicus	53	14	13	37184	109.78
A0A2I9LNW8	Protein D3	Centruroides hentzi	24	3	3	20341	106.27
A0A1E1WW95	Putative hemocyanin subunit	Tityus obscurus	18	12	3	72099	135.8

A0A0C9RFK9	TSA: Tityus bahiensis Tbah00905 mRNA sequence	Tityus bahiensis	19	7	2	44944	132.67
A0A3G5AP91	Saliva protein-15 (Fragment)	Tetranychus truncatus	40	3	3	13658	130.75
A0A1E1WWC5	Putative hemocyanin subunit	Tityus obscurus	14	8	4	71588	126.47
A0A1W7RAD3	Hemocyanin subunit 5	Hadrurus spadix	8	4	2	71708	94.8
C6H100	Hemocyanin subunit 5a	Pandinus imperator	17	9	4	71985	105.78
A0A1E1WVN1	Putative hemocyanin subunit	Tityus obscurus	14	7	4	72270	104.82
A0A1E1WVL0	Uncharacterized protein	Tityus obscurus	20	7	7	46784	104.67
A0A2H4RIG3	Elongation factor 1-alpha (Fragment)	Nanorchestes sp.	16	7	7	44786	98.68
A0A2H4RIR3	Elongation factor 1-alpha (Fragment)	Sancassania sp.	22	9	9	40969	98.68
A8UZ17	Elongation factor 1-alpha (Fragment)	Chortoglyphus arcuatus	19	8	8	41430	98.68
I1ZBX4	Elongation factor 1-alpha (Fragment)	Chortoglyphus sp.	19	8	8	41444	98.68
I1ZBX3	Elongation factor 1-alpha (Fragment)	Chortoglyphus sp.	18	7	7	38253	98.68
A8UY66	Elongation factor 1-alpha (Fragment)	Chortoglyphus sp.	18	7	7	38538	98.68
B0F547	Elongation factor 1-alpha (Fragment)	Sancassania aff. sphaerogaster	18	7	7	38238	98.68
I1ZBY2	Elongation factor 1-alpha (Fragment)	Horstia sp.	20	8	8	40787	98.68
I1ZBX7	Elongation factor 1-alpha (Fragment)	Oryzomyopus sp.	19	8	8	41322	98.68
A8UY99	Elongation factor 1-alpha (Fragment)	Naiadacarus arboricola	18	7	7	38214	98.68
I1ZBZ8	Elongation factor 1-alpha (Fragment)	Naiadacarus arboricola	18	7	7	38327	98.68
I1ZBY3	Elongation factor 1-alpha (Fragment)	Horstia glabra	18	7	7	38121	98.68
I1ZBY1	Elongation factor 1-alpha (Fragment)	Horstia sp.	18	7	7	38164	98.68
I1ZBZ9	Elongation factor 1-alpha (Fragment)	Askinasia sp.	18	7	7	38272	98.68
I1ZBZ4	Elongation factor 1-alpha (Fragment)	Capillaroglyphus polypori	18	7	7	38044	98.68
A8UYB4	Elongation factor 1-alpha (Fragment)	Sennertionyx manicati	18	7	7	38476	98.68
I1ZBZ3	Elongation factor 1-alpha (Fragment)	Aleuroglyphus ovatus	18	7	7	38517	98.68
I1ZC02	Elongation factor 1-alpha (Fragment)	Sennertionyx manicati	18	7	7	38547	98.68
A0A2H4RIG2	Elongation factor 1-alpha (Fragment)	Nanorchestes sp.	15	6	6	40220	98.68
A0A2H4RIG9	Elongation factor 1-alpha (Fragment)	Nanorchestes sp.	19	5	5	26109	98.68

A3F5A4	Elongation factor 1 alpha (Fragment)	Aleuroglyphus ovatus	25	5	5	19399	98.68
A0A1V9XXZ1	Histone H4	Tropilaelaps mercedesae	26	8	8	28105	98.47
A0A443SJI7	Histone H4	Leptotrombidium deliense	11	6	6	45915	98.47
A0A2L2Y3K7	Histone H4 (Fragment)	Parasteatoda tepidariorum	54	6	6	9262	98.47
A0A132ACK5	Histone H4	Sarcoptes scabiei	43	6	6	11367	98.47
Q4PM69	Histone H4	Ixodes scapularis	43	6	6	11367	98.47
D5J6Z3	Histone H4 (Fragment)	Pelinobius muticus	43	6	6	11367	98.47
A0A3G5APF0	Histone H4	Tetranychus evansi	43	6	6	11367	98.47
G3MN54	Histone H4	Amblyomma maculatum	43	6	6	11367	98.47
A0A3S4QJF9	Histone H4	Dinothrombium tinctorium	43	6	6	11367	98.47
E7D161	Histone H4 (Fragment)	Latrodectus hesperus	43	6	6	11367	98.47
A0A0N7I0H5	Histone H4	Dolomedes sulfureus	43	6	6	11367	98.47
A0A131Z809	Histone H4	Rhipicephalus appendiculatus	43	6	6	11367	98.47
A0A1V9XAM9	Histone H4	Tropilaelaps mercedesae	43	6	6	11367	98.47
A0A1W7RAF1	Histone H4	Hadrurus spadix	43	6	6	11367	98.47
A0A023FHA6	Histone H4	Amblyomma cajennense	43	6	6	11367	98.47
A0A443S2R0	Histone H4	Leptotrombidium deliense	43	6	6	11367	98.47
T1JYV7	Histone H4	Tetranychus urticae	43	6	6	11367	98.47
A0A1Z5LH91	Histone H4 (Fragment)	Ornithodoros moubata	43	6	6	11367	98.47
A0A087UHZ2	Histone H4 (Fragment)	Stegodyphus mimosarum	43	6	6	11367	98.47
A0A0K8RQR8	Histone H4 (Fragment)	Ixodes ricinus	42	6	6	11496	98.47
A0A1E1XVN0	Histone H4 (Fragment)	Amblyomma sculptum	42	6	6	11667	98.47
A0A1E1X193	Histone H4 (Fragment)	Amblyomma aureolatum	41	6	6	11772	98.47
A0A1E1X0M3	Histone H4 (Fragment)	Amblyomma aureolatum	41	6	6	11754	98.47
A0A3S3SL30	Histone H4	Dinothrombium tinctorium	41	6	6	11774	98.47
A0A131XP52	Histone H4 (Fragment)	Hyalomma excavatum	41	6	6	11859	98.47
A0A023FTG4	Histone H4 (Fragment)	Amblyomma parvum	40	6	6	12049	98.47

A0A1E1XSW7	Histone H4 (Fragment)	Amblyomma sculptum	40	6	6	12184	98.47
A0A131XJJ1	Histone H4 (Fragment)	Hyalomma excavatum	39	6	6	12574	98.47
A0A147B8K8	Histone H2A (Fragment)	Carios mimon	26	4	4	17456	54.67
A0A2L2XWA5	Histone H2A (Fragment)	Parasteatoda tepidariorum	40	4	4	11273	54.67
B7PSI5	Histone H2A	Ixodes scapularis	34	4	4	13364	54.67
A0A034WXE4	Histone H2A	Rhipicephalus microplus	34	4	4	13364	54.67
A0A443SPR7	Histone H2A	Leptotrombidium deliense	34	4	4	13332	54.67
A0A1E1WZS9	Histone H2A	Amblyomma aureolatum	34	4	4	13364	54.67
G3MN09	Histone H2A	Amblyomma maculatum	34	4	4	13364	54.67
A0A1Z5L6M4	Histone H2A (Fragment)	Ornithodoros moubata	34	4	4	13320	54.67
T1K5S4	Histone H2A	Tetranychus urticae	34	4	4	13304	54.67
A0A2R5LGI1	Histone H2A	Ornithodoros turicata	34	4	4	13364	54.67
A0A096XIA9	Histone H2A	Nuttalliella namaqua	34	4	4	13320	54.67
A0A1Y3BPP8	Histone H2A	Euroglyphus maynei	34	4	4	13321	54.67
A0A4Y2CXS9	Histone H2A	Araneus ventricosus	34	4	4	13348	54.67
A0A131YGT3	Histone H2A	Rhipicephalus appendiculatus	34	4	4	13364	54.67
G3MN11	Histone H2A	Amblyomma maculatum	34	4	4	13320	54.67
A0A0K8RIJ1	Histone H2A	Ixodes ricinus	34	4	4	13364	54.67
L7M671	Histone H2A	Rhipicephalus pulchellus	34	4	4	13364	54.67
A0A023FGQ4	Histone H2A	Amblyomma cajennense	34	4	4	13364	54.67
A0A1Z5KXS9	Histone H2A (Fragment)	Ornithodoros moubata	34	4	4	13364	54.67
A0A131XMJ1	Histone H2A	Hyalomma excavatum	34	4	4	13364	54.67
V5HJX3	Histone H2A	Ixodes ricinus	34	4	4	13648	54.67
A0A1V9X3Y7	Histone H2A	Tropilaelaps mercedesae	34	4	4	13408	54.67
A0A1V9X8R3	Histone H2A	Tropilaelaps mercedesae	34	4	4	13435	54.67
A0A0A0V6S1	Histone H2A (Fragment)	Scytodes thoracica	33	4	4	13566	54.67
A0A4Y2QZR4	Histone H2A	Araneus ventricosus	30	4	4	14806	54.67

A0A087UUJ7	Histone H2A (Fragment)	Stegodyphus mimosarum	29	4	4	15402	54.67
A0A4D5RWU4	Histone H2A (Fragment)	Ixodes scapularis	28	4	4	16247	54.67
A0A1V9X602	Histone H2A (Fragment)	Tropilaelaps mercedesae	35	3	3	9985	54.67
A0A023GLJ3	Putative heat shock protein	Amblyomma triste	8	4	2	73015	55.86
A0A293LNE1	Heat shock protein (Fragment)	Ornithodoros erraticus	8	4	2	70514	55.86
A0A443R284	Heat shock 70 kDa protein 1A/1B-like isoform X1	Dinothrombium tinctorium	8	4	2	70261	55.86
A0A1W7RAC4	Heat shock 70 kDa protein	Hadrurus spadix	8	4	2	70942	55.86
J7G3V2	Heat shock cognate protein 70 (Fragment)	Latrodectus hesperus	19	4	2	29928	55.86
A0A023FQ54	Putative heat shock protein (Fragment)	Amblyomma cajennense	10	4	2	57108	55.86
A5HLG0	Heat shock protein 70kDa (Fragment)	Kibramoa guapa	8	4	2	68813	55.86
A0A443RN36	Heat shock 70 kDa protein 1A/1B-like isoform X1	Dinothrombium tinctorium	8	4	2	70327	55.86
A0A1E1XLB1	Putative peptidylglycine alpha-amidating monooxygenase (Fragment)	Amblyomma sculptum	17	8	3	36177	98.18
F1CJ57	Hypothetical secreted protein	Hottentotta judaicus	21	3	3	17209	96.42
A0A4Y2UXR1	Polyubiquitin (Fragment)	Araneus ventricosus	10	5	5	59989	87.85
A0A1W7R9R2	Ribosomal protein L40	Hadrurus spadix	36	4	4	14701	87.85
A0A4D5RXG9	Putative polyubiquitin (Fragment)	Ixodes scapularis	16	4	4	31359	87.85
A0A023GMC0	Uncharacterized protein (Fragment)	Amblyomma triste	12	4	4	41950	87.85
A0A5J6YGB0	60S ribosomal protein L40A-like protein	Centruroides edwardsii	29	4	4	17902	87.85
B7SP62	Ubiquitin/ribosomal protein S27a fusion protein	Dermacentor variabilis	29	4	4	17915	87.85
Q09JW5	Ubiquitin/40S ribosomal protein S27a fusion protein	Argas monolakensis	20	4	4	26510	87.85
A0A0K8R331	Putative ubiquitin/40s ribosomal protein s27a fusion (Fragment)	Ixodes ricinus	25	4	4	21533	87.85
A0A1W7R9R4	60S ribosomal protein L40A	Hadrurus spadix	29	4	4	17979	87.85
A0A0K8R2I3	Putative ubiquitin/40s ribosomal protein s27a (Fragment)	Ixodes ricinus	28	3	3	13609	87.85
A0A023FGJ7	Uncharacterized protein (Fragment)	Amblyomma cajennense	32	4	4	15981	87.85
A0A023GEE1	Putative ubiquitin-40s ribosomal protein s27a (Fragment)	Amblyomma triste	51	4	4	10334	87.85
A0A0C9S1S7	Putative polyubiquitin	Amblyomma americanum	20	4	4	25821	87.85
F0J9P3	Ubiquitin (Fragment)	Amblyomma variegatum	19	4	4	27093	87.85

A0A1E1XER6	Putative ubiquitin c variant (Fragment)	Amblyomma aureolatum	12	4	4	44587	87.85
F1CJ00	Hypothetical secreted protein	Hottentotta judaicus	18	2	2	13139	85.16
A0A2I9LNZ2	Endoplasmic reticulum resident protein 44	Centruroides hentzi	10	3	3	48319	83.9
A0A1Y3B181	Disulfide-isomerase-like protein (Fragment)	Euroglyphus maynei	20	3	2	28323	74.95
A0A2R5L496	Glyceraldehyde-3-phosphate dehydrogenase (Fragment)	Ornithodoros turicata	13	3	2	35474	72.97
B7QJH0	Thioredoxin peroxidase putative	Ixodes scapularis	12	3	2	31992	60.51
B0LUH3	Thioredoxin peroxidase	Ixodes ricinus	14	3	2	27978	60.51
Q4PN30	Thioredoxin peroxidase	Ixodes scapularis	14	3	2	27974	60.51
A0A2P6K1Z0	Rho	Nephila clavipes	5	2	2	61272	52.37
A0A1W7RAP7	Protein D3	Hadrurus spadix	18	2	2	20187	60.74
A0A1W7R9Z2	Protein disulfide-isomerase	Hadrurus spadix	9	4	2	56388	52.39
G3MFC4	Tubulin alpha chain (Fragment)	Amblyomma maculatum	10	3	2	49049	50.88
A0A3S4R6E6	Tubulin alpha chain	Dinothrombium tinctorium	8	3	2	50094	50.88
A0A2I9LP74	Peptidylglycine alpha-amidating monooxygenase	Centruroides hentzi	25	11	9	39030	50.16
A0A4Y5UGG0	Peptidylglycine alpha-amidating monooxygenase	Cupiennius salei	7	3	3	39691	49.4
A0A0C9RP93	TSA: Tityus bahiensis Tbah00758 mRNA sequence	Tityus bahiensis	11	4	4	50989	47.68
A0A131XZT3	Putative synaptic vesicle membrane protein vat-1 log-like isoform x1	Ixodes ricinus	5	2	2	45581	45.77
A0A131XTI5	Putative synaptic vesicle membrane protein vat-1 log-like isoform x1	Ixodes ricinus	5	2	2	47174	45.77
A0A4D5RL55	Putative vesicle amine transport protein isoform x1 (Fragment)	Ixodes scapularis	5	2	2	47973	45.77
A0A0K8RCN0	Putative quinone oxidoreductase	Ixodes ricinus	5	2	2	45507	45.77
A0A1S4M2U8	Quinone oxidoreductase putative	Ixodes scapularis	6	2	2	39836	45.77

Table S2. Identified protein families in *Hottentotta saulcyi* venom by in-gel tryptic digestion.

Accession #	Description	Organism	Molecular Mass (kDa)
**Gel Spot # 1**			
F1CIW7	Iota-buthitoxin-Hj1b	*Hottentotta judaicus*	9.6
Q2NNB8	Toxin1 (Fragment)	Hottentotta saulcyi	7.4
F1CJ05	Putative toxin (Fragment)	Hottentotta judaicus	4.1
A0A0U4GT75	Sodium channel toxin NaTx8	Odontobuthus doriae	11
A0A2I9LPD1	Nucleoside diphosphate kinase	Centruroides hentzi	19.4
F0V3V8	Insect excitatory neurotoxin precusor	Hottentotta judaicus	10.5
A0A088D9U2	Potassium channel blocker pMeKTx28-2	Mesobuthus eupeus	10.3
A0A0U4QF63	Sodium channel toxin NaTx7	Odontobuthus doriae	10.9

A0A059UI30	Potassium channel toxin Meg-beta-KTx1	Mesobuthus gibbosus	10.2
P17728	Alpha-insect toxin LqhaIT	Leiurus hebraeus	9.6
F1CIV8	Beta-buthitoxin-Hj2a	Hottentotta judaicus	9.6
**Gel Spot # 2**			
A0A2I9LNW8	Protein D3	Centruroides hentzi	20.3
A0A0K0LCJ8	Cellular protein AbCp-5 (Fragment)	Androctonus bicolor	8.3
A0A0K8RMV8	Annexin (Fragment)	Ixodes ricinus	17.5
A0A1Z5L7J4	40S ribosomal protein S7 (Fragment)	Ornithodoros moubata	12.2
	Putative beta actin variant (Fragment)	Ixodes scapularis	44.4
F1CIY0	Hypothetical secreted protein (Fragment)	Hottentotta judaicus	25.5
F1CJ98	Putative glutaminyl cyclase (Fragment)	Hottentotta judaicus	19
F1CIW1	Putative trypsin/chemotrypsin-like S1/S6 peptidase	Hottentotta judaicus	17.8
F1CIY2	Putative transmembranal serine protease (Fragment)	Hottentotta judaicus	25.1
A0A0U1TYH0	Tetraspanin (Fragment)	Isometrus maculatus	26.2
A0A1E1XQN6	Putative cdk5 regulatory subunit associated protein 1-like 1 (Fragment)	Amblyomma sculptum	48.7
F1CIU8	Putative M12B metalloprotease (Fragment)	Hottentotta judaicus	38.5
**Gel Spot # 3**			
A0A1W7RB61	Alpha-enolase	Hadrurus spadix	46.6
A5HLG2	Heat shock protein 70kDa (Fragment)	Plectreurys tecate	68.6
E4VNZ7	Venom metalloprotease-1	Mesobuthus eupeus	44.8
F1CJ75	Putative cystein-rich secretory peptide (Fragment)	Hottentotta judaicus	23.9
F1CJ26	M12B metalloprotease (Fragment)	Hottentotta judaicus	27.6
A0A4D5RKV1	Putative serine proteinase inhibitor (Fragment)	Ixodes scapularis	50.9
A0A0C9RP88	TSA: Tityus bahiensis Tbah00853 mRNA sequence	Tityus bahiensis	47.6
A0A443SJI7	Histone H4 OS=Leptotrombidium deliense	Leptotrombidium deliense	45.9
A0A3S4R6E6	Tubulin alpha chain	Dinothrombium tinctorium	50.1
A0A3G5AP15	L Chain L, Crystal Structure Of Fab Dx-2930	Tetranychus truncatus	18.1
A0A2R5LFR2	ATP synthase subunit beta (Fragment)	Ornithodoros turicata	56.7
F8THJ4	CRISP3 (Fragment)	Hottentotta judaicus	21.2
A0A293LZG0	Glyceraldehyde-3-phosphate dehydrogenase (Fragment)	Ornithodoros erraticus	36.2
A0A3G5AP91	Saliva protein-15 (Fragment)	Tetranychus truncatus	13.6
A0A443QDL4	Actin-like protein	Dinothrombium tinctorium	41.8
I1ZBY2	Elongation factor 1-alpha (Fragment)	Horstia sp.	40.8
**Gel Spot # 4**			
A0A1S5QN46	Carboxypeptidase E	Tityus serrulatus	53.8
A0A1E1XMD8	Putative cysteine proteinase (Fragment)	Amblyomma sculptum	53.3
P86100	Hyaluronidase-1	Mesobuthus martensii	47.4
A0A0U1SF04	Peptidase_M14 domain-containing protein (Fragment)	Isometrus maculatus	23.1
A0A218QX58	Putative cysteine-rich protein	Tityus serrulatus	44.8
A0A2I9LP14	Hemocyanin subunit F	Centruroides hentzi	71.9
F1CJ13	Thioredoxin domain-containing protein (Fragment)	Hottentotta judaicus	37.2
A0A2I9LNZ2	Endoplasmic reticulum resident protein 44	Centruroides hentzi	48.3

A0A087TJM9	Carboxypeptidase E (Fragment)	Stegodyphus mimosarum	52.1
A0A1W7RAV1	Carboxypeptidase	Hadrurus spadix	49
B7QJH0	Thioredoxin peroxidase, putative	Ixodes scapularis	32
P80476	Hemocyanin AA6 chain	Androctonus australis	71.7
A0A2I9LPB9	Neuroendocrine convertase 1	Centruroides hentzi	77.7
F1CJ25	Putative angiotensin converting enzyme (Fragment)	Hottentotta judaicus	30.5
F1CJ87	Putative angiotensin-converting enzyme (Fragment)	Hottentotta judaicus	
A0A1E1WVT7	Angiotensin-converting enzyme	Tityus obscurus	72.8
F8THK0	Putative RNA-binding protein (Fragment)	Hottentotta judaicus	54.2
L7M2K4	Amino acid transporter	Rhipicephalus pulchellus	51.7
A0A1E1WWC5	Putative hemocyanin subunit	Tityus obscurus	71.5
A0A2P6K1Z0	Rho	Nephila clavipes	61.2
A0A1V9X9D1	Protein dopey-1-like (Fragment)	Tropilaelaps mercedesae	255
A0A1E1WVL7	Putative hemolectin	Tityus obscurus	37.9
C5J8B0	Hemocyanin-like peptide (Fragment)	Opisthacanthus cayaporum	20.5
A0A2I9LPG6	Polypeptide N-acetylgalactosaminyltransferase	Centruroides hentzi	67.8
A0A2L2Y9E8	Hemocyanin subunit G (Fragment)	Parasteatoda tepidariorum	17.3
A0A1E1WVN1	Putative hemocyanin subunit	Tityus obscurus	72.2
A0A1S5QN46	Carboxypeptidase E	Tityus serrulatus	53.8
A0A1E1XMD8	Putative cysteine proteinase (Fragment)	Amblyomma sculptum	53.3
P86100	Hyaluronidase-1	Mesobuthus martensii	47.4
A0A0U1SF04	Peptidase_M14 domain-containing protein (Fragment)	Isometrus maculatus	23.1
A0A218QX58	Putative cysteine-rich protein	Tityus serrulatus	44.8

Table S3. List of all top-ranked identified lipids from *Hottentotta saulcyi* venom in positive-ion mode. The data obtained by LipidMatch software and the formula of the neutral lipids as well as the theoretical m/z values were obtained using the LipidPioneer template.[1].

Formula of Neutral Lipid	Adducts Confirmed	Experimental *m/z*	Theoretical *m/z*	Lipids
$C_{17}H_{27}NO_4$	[M+H]+	310.2016	310.2013	AcCar(10:3)
$C_{17}H_{33}NO_4$	[M+H]+	316.2486	316.2482	AcCar(10:0)
$C_{21}H_{39}NO_4$	[M+H]+	370.2960	370.2952	AcCar(14:1)
$C_{23}H_{45}NO_4$	[M+H]+	400.3430	400.3421	AcCar(16:0)
$C_{27}H_{53}NO_4$	[M+H]+	456.4061	456.4047	AcCar(20:0)
$C_{29}H_{57}NO_4$	[M+H]+	484.4364	484.4360	AcCar(22:0)
$C_{42}H_{79}O_{10}P$	[M+NH4]+	792.5757	792.5749	BMP(18:1_18:1)
$C_{42}H_{81}O_{10}P$	[M+NH4]+	794.5915	794.5905	BMP(18:0_18:1)
$C_{54}H_{110}NO_{10}P$	[M+NH4]+	964.7892	964.7940	BMP(22:0_26:0)
$C_{32}H_{63}NO_3$	[M+H]+	510.4890	510.4880	Cer(d16:1/16:0)
$C_{34}H_{67}NO_3$	[M+H]+	538.5198	538.5193	Cer(d18:1/16:0)
$C_{34}H_{69}NO_3$	[M+H]+	540.5341	540.5350	Cer(d18:0/16:0)
$C_{36}H_{65}NO_3$	[M+H]+	560.5019	560.5037	Cer(d18:2/18:2)
$C_{36}H_{69}NO_3$	[M+H]+	564.5361	564.5350	Cer(d20:2/16:0)
$C_{36}H_{71}NO_3$	[M+H]+	566.5498	566.5506	Cer(d16:1/20:0)
$C_{38}H_{73}NO_3$	[M+H]+	592.5678	592.5663	Cer(d20:2/18:0)
$C_{38}H_{75}NO_3$	[M+H]+	594.5822	594.5819	Cer(d16:1/22:0)
$C_{38}H_{77}NO_3$	[M+H]+	596.5969	596.5976	Cer(d16:0/22:0)

$C_{40}H_{73}NO_3$	[M+H]+	616.5645	616.5663	Cer(d20:2/20:2)
$C_{40}H_{77}NO_3$	[M+H]+	620.5976	620.5976	Cer(d20:2/20:0)
$C_{40}H_{79}NO_3$	[M+H]+	622.6132	622.6132	Cer(d18:1/22:0)
$C_{42}H_{77}NO_3$	[M+H]+	644.5963	644.5976	Cer(d20:2/22:2)
$C_{42}H_{81}NO_3$	[M+H]+	648.6273	648.6289	Cer(d20:2/22:0)
$C_{44}H_{85}NO_3$	[M+H]+	676.6610	676.6602	Cer(d20:2/24:0)
$C_{49}H_{95}NO_3$	[M+H]+	746.7386	746.7384	Cer(d20:2/29:0)
$C_{50}H_{97}NO_3$	[M+H]+	760.7532	760.7541	Cer(d22:2/28:0)
$C_{60}H_{117}NO_3$	[M+H]+	900.9076	900.9106	Cer(d18:1/42:1)
$C_{35}H_{66}O_5$	[M+NH4]+	584.5265	584.5248	DG(16:0_16:1)
$C_{37}H_{70}O_5$	[M+NH4]+	612.5578	612.5561	DG(16:0_18:1)
$C_{39}H_{66}O_5$	[M+NH4]+	632.5263	632.5248	DG(18:2_18:3)
$C_{39}H_{68}O_5$	[M+NH4]+	634.5413	634.5405	DG(18:2_18:2)
$C_{39}H_{70}O_5$	[M+NH4]+	636.5589	636.5561	DG(18:1_18:2)
$C_{39}H_{72}O_5$	[M+NH4]+	638.5729	638.5718	DG(18:1_18:1)
$C_{42}H_{74}O_{15}$	[M+NH4]+	836.5414	836.5370	DGDG(9:0_18:2)
$C_{44}H_{81}NO_7$	[M+H]+	736.6076	736.6086	DGTS(16:0_18:2)
$C_{24}H_{50}NO_6P$	[M+H]+	480.3454	480.3448	Ether-LPC(O-16:1)
$C_{26}H_{54}NO_6P$	[M+H]+	508.3771	508.3761	Ether-LPC(O-18:1)
$C_{21}H_{44}NO_6P$	[M+H]+	438.2969	438.2979	Ether-LPE(P-16:0)
$C_{21}H_{46}NO_6P$	[M+H]+	440.3143	440.3135	Ether-LPE(O-16:0)
$C_{23}H_{48}NO_6P$	[M+H]+	466.3280	466.3292	Ether-LPE(P-18:0)
$C_{23}H_{50}NO_6P$	[M+H]+	468.3463	468.3448	Ether-LPE(O-18:0)
$C_{38}H_{78}NO_7P$	[M+H]+	692.5592	692.5588	Ether-PC(O-16:0/14:0)
$C_{39}H_{80}NO_7P$	[M+H]+	706.5751	706.5745	Ether-PC(O-16:0/15:0)
$C_{40}H_{80}NO_7P$	[M+H]+	718.5732	718.5745	Ether-PC(O-16:0/16:1)
$C_{40}H_{82}NO_7P$	[M+H]+	720.5894	720.5901	Ether-PC(O-16:0/16:0)
$C_{41}H_{82}NO_7P$	[M+H]+	732.5894	732.5901	Ether-PC(O-16:0/17:1)
$C_{42}H_{80}NO_7P$	[M+H]+	742.5748	742.5745	Ether-PC(P-16:0/18:2)
$C_{44}H_{78}NO_7P$	[M+H]+	764.5602	764.5588	Ether-PC(O-16:1/20:5)
$C_{44}H_{80}NO_7P$	[M+H]+	766.5751	766.5745	Ether-PC(O-16:0/20:5)
$C_{44}H_{82}NO_7P$	[M+H]+	768.5903	768.5901	Ether-PC(O-16:0/20:4)
$C_{44}H_{84}NO_7P$	[M+H]+	770.6056	770.6058	Ether-PC(P-18:0/18:2)
$C_{44}H_{86}NO_7P$	[M+H]+	772.6215	772.6214	Ether-PC(P-18:0/18:1)
$C_{44}H_{88}NO_7P$	[M+H]+	774.6369	774.6371	Ether-PC(O-18:0/18:1)
$C_{46}H_{82}NO_7P$	[M+H]+	792.5909	792.5901	Ether-PC(P-18:0/20:5)
$C_{46}H_{84}NO_7P$	[M+H]+	794.6058	794.6058	Ether-PC(P-18:0/20:4)
$C_{46}H_{90}NO_7P$	[M+H]+	800.6528	800.6527	Ether-PC(O-20:0/18:2)
$C_{46}H_{92}NO_7P$	[M+H]+	802.6732	802.6684	Ether-PC(O-20:0/18:1)
$C_{48}H_{88}NO_7P$	[M+H]+	822.6395	822.6371	Ether-PC(P-20:0/20:4)
$C_{48}H_{90}NO_7P$	[M+H]+	824.6564	824.6527	Ether-PC(O-20:0/20:4)
$C_{39}H_{74}NO_7P$	[M+H]+	700.5287	700.5275	Ether-PE(P-16:0/18:2)
$C_{39}H_{76}NO_7P$	[M+H]+	702.5432	702.5432	Ether-PE(P-16:0/18:1)
$C_{39}H_{78}NO_7P$	[M+H]+	704.5556	704.5588	Ether-PE(O-16:0/18:1)
$C_{40}H_{76}NO_7P$	[M+H]+	714.5437	714.5432	Ether-PE(P-18:1/17:1)
$C_{41}H_{72}NO_7P$	[M+H]+	722.5130	722.5119	Ether-PE(P-16:0/20:5)
$C_{41}H_{74}NO_7P$	[M+H]+	724.5289	724.5275	Ether-PE(P-16:0/20:4)
$C_{41}H_{78}NO_7P$	[M+H]+	728.5593	728.5588	Ether-PE(P-18:0/18:2)
$C_{41}H_{80}NO_7P$	[M+H]+	730.5749	730.5745	Ether-PE(P-18:0/18:1)

$C_{43}H_{74}NO_7P$	[M+H]+	748.5285	748.5275	Ether-PE(P-18:1/20:5)
$C_{43}H_{76}NO_7P$	[M+H]+	750.5439	750.5432	Ether-PE(P-18:1/20:4)
$C_{43}H_{82}NO_7P$	[M+H]+	756.5906	756.5901	Ether-PE(P-20:1/18:1)
$C_{43}H_{84}NO_7P$	[M+H]+	758.6076	758.6058	Ether-PE(P-20:0/18:1)
$C_{45}H_{80}NO_7P$	[M+H]+	778.5755	778.5745	Ether-PE(P-20:1/20:4)
$C_{45}H_{82}NO_7P$	[M+H]+	780.5907	780.5901	Ether-PE(P-20:0/20:4)
$C_{45}H_{84}NO_7P$	[M+H]+	782.6073	782.6058	Ether-PE(P-20:0/20:3)
$C_{44}H_{83}NO_8$	[M+H]+	754.6203	754.6191	GlcCer(d16:1/22:1)
$C_{44}H_{85}NO_8$	[M+H]+	756.6383	756.6348	GlcCer(d16:1/22:0)
$C_{65}H_{123}NO_8$	[M+H]+	1046.9285	1046.9321	GlcCer(d17:1/42:2)
$C_{22}H_{46}NO_7P$	[M+H]+	468.3093	468.3084	LPC(14:0)
$C_{24}H_{48}NO_7P$	[M+H]+	494.3251	494.3241	LPC(16:1)
$C_{25}H_{52}NO_7P$	[M+H]+	510.3568	510.3554	LPC(17:0)
$C_{26}H_{48}NO_7P$	[M+H]+	518.3232	518.3241	LPC(18:3)
$C_{26}H_{50}NO_7P$	[M+H]+ I [M+Na]+	520.3409	520.3397	LPC(18:2)
$C_{26}H_{52}NO_7P$	[M+H]+ I [M+Na]+	522.3568	522.3554	LPC(18:1)
$C_{26}H_{54}NO_7P$	[M+H]+ I [M+Na]+	524.3724	524.3710	LPC(18:0)
$C_{27}H_{56}NO_7P$	[M+H]+	538.3886	538.3867	LPC(19:0)
$C_{28}H_{48}NO_7P$	[M+H]+	542.3250	542.3241	LPC(20:5)
$C_{28}H_{50}NO_7P$	[M+H]+	544.3407	544.3397	LPC(20:4)
$C_{28}H_{52}NO_7P$	[M+H]+	546.3547	546.3554	LPC(20:3)
$C_{28}H_{58}NO_7P$	[M+H]+	552.4047	552.4023	LPC(20:0)
$C_{23}H_{44}NO_7P$	[M+H]+	478.2930	478.2928	LPE(18:2)
$C_{23}H_{46}NO_7P$	[M+H]+ I [M+Na]+	480.3089	480.3084	LPE(18:1)
$C_{23}H_{48}NO_7P$	[M+H]+ I [M+Na]+	482.3254	482.3241	LPE(18:0)
$C_{25}H_{44}NO_7P$	[M+H]+	502.2930	502.2928	LPE(20:4)
$C_{25}H_{42}O_4$	[M+NH4]+	424.3427	424.3421	MG(22:4)
$C_{46}H_{82}O_{10}$	[M+NH4-CO]+	784.6286	784.6297	MGDG(15:1_22:2)
$C_{50}H_{88}O_{10}$	[M+NH4-CO]+	838.6784	838.6766	MGDG(17:0_24:4)
$C_{25}H_{48}NO_9P$	[M+H]+	538.3157	538.3139	OxLPC(17:2(OO))
$C_{25}H_{50}NO_9P$	[M+H]+	540.3318	540.3296	OxLPC(17:1(OO))
$C_{28}H_{54}NO_9P$	[M+H]+	580.3625	580.3609	OxLPC(20:2(OO))
$C_{28}H_{48}NO_{10}P$	[M+H]+	590.3082	590.3088	OxLPC(20:5(OOO))
$C_{30}H_{60}NO_8P$	[M+H]+	594.4156	594.4129	OxLPC(22:1(OH))
$C_{28}H_{54}NO_{10}P$	[M+H]+	596.3569	596.3558	OxLPC(20:2(OOO))
$C_{30}H_{50}NO_9P$	[M+H]+	600.3285	600.3296	OxLPC(22:6(OO))
$C_{30}H_{58}NO_9P$	[M+H]+	608.3945	608.3922	OxLPC(22:2(OO))
$C_{32}H_{64}NO_8P$	[M+H]+	622.4458	622.4442	OxLPC(24:1(OH))
$C_{32}H_{62}NO_9P$	[M+H]+	636.4237	636.4235	OxLPC(24:1(KeOH))
$C_{32}H_{64}NO_9P$	[M+H]+	638.4401	638.4391	OxLPC(24:1(OO))
$C_{32}H_{64}NO_{10}P$	[M+H]+	654.4337	654.4340	OxLPC(24:1(OOO))
$C_{27}H_{52}NO_8P$	[M+H]+	550.3517	550.3503	OxLPE(22:2(OH))
$C_{29}H_{58}NO_8P$	[M+H]+	580.3993	580.3973	OxLPE(24:1(OH))
$C_{29}H_{58}NO_9P$	[M+H]+	596.3937	596.3922	OxLPE(24:1(OO))
$C_{29}H_{56}NO_9P$	[M+H]+	594.3768	594.3765	OxPC(16:0_5:0(CHO))
$C_{29}H_{56}NO_{10}P$	[M+H]+	610.3726	610.3714	OxPC(16:0_5:0(COOH))
$C_{31}H_{58}NO_9P$	[M+H]+	620.3935	620.3922	OxPC(18:1_5:0(CHO))
$C_{31}H_{60}NO_9P$	[M+H]+	622.4099	622.4078	OxPC(18:0_5:0(CHO))
$C_{31}H_{56}NO_{10}P$	[M+H]+	634.3734	634.3714	OxPC(17:2(OH)_6:0(CHO))

$C_{31}H_{58}NO_{10}P$	[M+H]+	636.3866	636.3871	OxPC(18:1_5:0(COOH))
$C_{31}H_{60}NO_{10}P$	[M+H]+	638.4041	638.4027	OxPC(18:0_5:0(COOH))
$C_{32}H_{58}NO_{10}P$	[M+H]+	648.3899	648.3871	OxPC(18:1_6:1(COOH))
$C_{33}H_{62}NO_{9}P$	[M+H]+	648.4243	648.4235	OxPC(18:1_7:0(CHO))
$C_{33}H_{64}NO_{9}P$	[M+H]+	650.4407	650.4391	OxPC(16:0_9:0(CHO))
$C_{32}H_{62}NO_{10}P$	[M+H]+	652.4189	652.4184	OxPC(16:0_8:0(COOH))
$C_{33}H_{58}NO_{10}P$	[M+H]+	660.3869	660.3871	OxPC(15:1(OH)_10:2(CHO))
$C_{33}H_{62}NO_{10}P$	[M+H]+	664.4207	664.4184	OxPC(18:1_7:0(COOH))
$C_{33}H_{64}NO_{10}P$	[M+H]+ǀ[M+Na]+	666.4352	666.4340	OxPC(16:0_9:0(COOH))
$C_{35}H_{66}NO_{9}P$	[M+H]+	676.4559	676.4548	OxPC(18:1_9:0(CHO))
$C_{34}H_{64}NO_{10}P$	[M+H]+	678.4350	678.4340	OxPC(18:1_8:0(COOH))
$C_{34}H_{66}NO_{10}P$	[M+H]+	680.4512	680.4497	OxPC(18:0_8:0(COOH))
$C_{36}H_{64}NO_{9}P$	[M+H]+	686.4401	686.4391	OxPC(18:1_10:2(CHO))
$C_{34}H_{62}NO_{11}P$	[M+H]+	692.4142	692.4133	OxPC(17:2(OH)_9:0(COOH))
$C_{35}H_{68}NO_{10}P$	[M+H]+ǀ[M+Na]+	694.4663	694.4653	OxPC(18:0_9:0(COOH))
$C_{37}H_{72}NO_{9}P$	[M+H]+	706.4978	706.5017	OxPC(14:0_15:0(CHO))
$C_{35}H_{66}NO_{11}P$	[M+H]+	708.4451	708.4446	OxPC(18:1(OH)_9:0(COOH))
$C_{38}H_{70}NO_{9}P$	[M+H]+	716.4870	716.4861	OxPC(18:0_12:2(CHO))
$C_{38}H_{74}NO_{9}P$	[M+H]+	720.5131	720.5174	OxPC(16:0_14:0(CHO))
$C_{38}H_{68}NO_{11}P$	[M+H]+	746.4562	746.4602	OxPC(18:2(OH)_12:1(COOH))
$C_{40}H_{76}NO_{9}P$	[M+H]+	746.5342	746.5330	OxPC(14:0_18:2(OH))
$C_{41}H_{74}NO_{9}P$	[M+H]+	756.5197	756.5174	OxPC(18:3_15:0(CHO))
$C_{41}H_{78}NO_{9}P$	[M+H]+	760.5479	760.5487	OxPC(15:1_18:1(OH))
$C_{40}H_{78}NO_{10}P$	[M+H]+	764.5463	764.5436	OxPC(15:0_17:1(OO))
$C_{42}H_{78}NO_{9}P$	[M+H]+	772.5497	772.5487	OxPC(16:0_18:3(OH))
$C_{42}H_{80}NO_{9}P$	[M+H]+	774.5655	774.5643	OxPC(16:0_18:2(OH))
$C_{43}H_{70}NO_{9}P$	[M+H]+	776.4844	776.4861	OxPC(20:5_15:2(CHO))
$C_{41}H_{78}NO_{10}P$	[M+H]+	776.5437	776.5436	OxPC(15:0_18:2(OOH))
$C_{42}H_{82}NO_{9}P$	[M+H]+	776.5800	776.5800	OxPC(16:0_18:1(OH))
$C_{43}H_{76}NO_{9}P$	[M+H]+	782.5328	782.5330	OxPC(18:3_17:2(OH))
$C_{43}H_{78}NO_{9}P$	[M+H]+	784.5491	784.5487	OxPC(18:2_17:2(OH))
$C_{43}H_{80}NO_{9}P$	[M+H]+	786.5652	786.5643	OxPC(18:1_17:2(OH))
$C_{41}H_{74}NO_{11}P$	[M+H]+	788.5088	788.5072	OxPC(15:1(OH)_18:3(OO))
$C_{43}H_{82}NO_{9}P$	[M+H]+	788.5785	788.5800	OxPC(18:0_17:2(OH))
$C_{42}H_{72}NO_{11}P$	[M+H]+	798.4934	798.4915	OxPC(18:3(OH)_16:2(COOH))
$C_{44}H_{82}NO_{9}P$	[M+H]+	800.5808	800.5800	OxPC(18:1_18:2(OH))
$C_{44}H_{84}NO_{9}P$	[M+H]+	802.5958	802.5956	OxPC(18:1_18:1(OH))
$C_{44}H_{86}NO_{9}P$	[M+H]+	804.6100	804.6113	OxPC(18:0_18:1(OH))
$C_{41}H_{76}NO_{12}P$	[M+H]+	806.5196	806.5178	OxPC(24:1(OOO)_9:1(CHO))
$C_{42}H_{80}NO_{11}P$	[M+H]+	806.5569	806.5541	OxPC(16:0_18:2(OOO))
$C_{42}H_{82}NO_{11}P$	[M+H]+	808.5710	808.5698	OxPC(16:0_18:1(OOO))
$C_{43}H_{76}NO_{11}P$	[M+H]+	814.5243	814.5228	OxPC(17:2(OH)_18:3(OO))
$C_{44}H_{80}NO_{10}P$	[M+H]+	814.5603	814.5592	OxPC(18:1_18:3(OO))
$C_{45}H_{70}NO_{10}P$	[M+H]+	816.4859	816.4810	OxPC(20:5_17:4(COOH))

$C_{42}H_{74}NO_{12}P$	[M+H]+	816.5027	816.5021	OxPC(18:1(OO)_16:3(COOH))
$C_{43}H_{78}NO_{11}P$	[M+H]+	816.5386	816.5385	OxPC(18:2_17:2(OOO))
$C_{44}H_{82}NO_{10}P$	[M+H]+	816.5753	816.5749	OxPC(18:1_18:2(OOH))
$C_{43}H_{80}NO_{11}P$	[M+H]+	818.5545	818.5541	OxPC(18:2_17:1(OOO))
$C_{44}H_{84}NO_{10}P$	[M+H]+	818.5910	818.5905	OxPC(18:1_18:1(OO))
$C_{44}H_{86}NO_{10}P$	[M+H]+	820.6065	820.6062	OxPC(18:0_18:1(OO))
$C_{46}H_{80}NO_{9}P$	[M+H]+	822.5639	822.5643	OxPC(18:1_20:4(Ke))
$C_{44}H_{76}NO_{11}P$	[M+H]+	826.5227	826.5228	OxPC(18:3_18:3(OOO))
$C_{44}H_{78}NO_{11}P$	[M+H]+	828.5399	828.5385	OxPC(18:2(OH)_18:3(OO))
$C_{43}H_{76}NO_{12}P$	[M+H]+	830.5178	830.5178	OxPC(18:2(OOH)_17:2(KeOH))
$C_{44}H_{80}NO_{11}P$	[M+H]+	830.5559	830.5541	OxPC(18:2(OOH)_18:2(OH))
$C_{46}H_{88}NO_{9}P$	[M+H]+	830.6249	830.6269	OxPC(18:1_20:1(OH))
$C_{43}H_{78}NO_{12}P$	[M+H]+	832.5332	832.5334	OxPC(18:2(OH)_17:2(OOO))
$C_{44}H_{82}NO_{11}P$	[M+H]+	832.5722	832.5698	OxPC(18:1_18:2(OOO))
$C_{45}H_{72}NO_{11}P$	[M+H]+	834.4897	834.4915	OxPC(18:3(OH)_19:5(COOH))
$C_{43}H_{80}NO_{12}P$	[M+H]+	834.5499	834.5491	OxPC(18:1_17:2(OOOO))
$C_{44}H_{84}NO_{11}P$	[M+H]+	834.5851	834.5854	OxPC(18:0_18:2(OOO))
$C_{46}H_{78}NO_{10}P$	[M+H]+	836.5434	836.5436	OxPC(18:1_20:5(KeOH))
$C_{47}H_{82}NO_{9}P$	[M+H]+	836.5774	836.5800	OxPC(20:0_19:5(CHO))
$C_{43}H_{76}NO_{13}P$	[M+H]+	846.5119	846.5127	OxPC(18:3(OO)_17:2(OOO))
$C_{44}H_{80}NO_{12}P$	[M+H]+	846.5510	846.5491	OxPC(18:1(OO)_18:3(OO))
$C_{43}H_{78}NO_{13}P$	[M+H]+	848.5267	848.5283	OxPC(18:2(OH)_17:2(OOOO))
$C_{44}H_{82}NO_{12}P$	[M+H]+	848.5663	848.5647	OxPC(18:2(OOH)_18:1(OO))
$C_{43}H_{68}NO_{14}P$	[M+H]+	854.4420	854.4450	OxPC(22:5(OOOO)_13:3(COOH))
$C_{45}H_{76}NO_{12}P$	[M+H]+	854.5165	854.5178	OxPC(20:4(Ke)_17:2(OOO))
$C_{46}H_{80}NO_{11}P$	[M+H]+	854.5553	854.5541	OxPC(18:1_20:5(OOO))
$C_{46}H_{82}NO_{11}P$	[M+H]+	856.5702	856.5698	OxPC(18:1_20:4(OOO))
$C_{48}H_{92}NO_{11}P$	[M+H]+	890.6459	890.6480	OxPC(23:0_17:2(OOO))
$C_{32}H_{54}NO_{9}P$	[M+H]+	628.3605	628.3609	OxPE(17:2_10:2(CHO))
$C_{32}H_{60}NO_{9}P$	[M+H]+	634.4089	634.4078	OxPE(18:1_9:0(CHO))
$C_{33}H_{60}NO_{9}P$	[M+H]+	646.4093	646.4078	OxPE(18:0_10:2(CHO))
$C_{32}H_{60}NO_{10}P$	[M+H]+	650.4036	650.4027	OxPE(18:1_9:0(COOH))
$C_{31}H_{58}NO_{12}P$	[M+H]+	668.3784	668.3769	OxPE(20:1(OOO)_6:0(CHO))
$C_{35}H_{64}NO_{9}P$	[M+H]+	674.4390	674.4391	OxPE(18:0_12:2(CHO))
$C_{33}H_{60}NO_{11}P$	[M+H]+	678.3980	678.3977	OxPE(17:1(OO)_11:1(CHO))
$C_{34}H_{58}NO_{11}P$	[M+H]+	688.3823	688.3820	OxPE(18:3(OH)_11:1(COOH))
$C_{36}H_{62}NO_{10}P$	[M+H]+	700.4178	700.4184	OxPE(18:1_13:3(COOH))
$C_{37}H_{66}NO_{9}P$	[M+H]+	700.4552	700.4548	OxPE(18:1_14:2(CHO))

$C_{37}H_{68}NO_9P$	[M+H]+	702.4694	702.4704	OxPE(18:0_14:2(CHO))
$C_{41}H_{76}NO_9P$	[M+H]+	758.5336	758.5330	OxPE(18:0_18:3(OH))
$C_{43}H_{72}NO_9P$	[M+H]+	778.5014	778.5017	OxPE(18:1_20:5(Ke))
$C_{43}H_{70}NO_{10}P$	[M+H]+	792.4819	792.4810	OxPE(20:5_18:3(OO))
$C_{43}H_{70}NO_{11}P$	[M+H]+	808.4771	808.4759	OxPE(18:3(OH)_20:5(OO))
$C_{45}H_{68}NO_{10}P$	[M+H]+	814.4668	814.4653	OxPE(20:5_20:5(KeOH))
$C_{43}H_{74}NO_{12}P$	[M+H]+	828.5029	828.5021	OxPE(18:1_20:5(OOOO))
$C_{45}H_{74}NO_{11}P$	[M+H]+	836.5058	836.5072	OxPE(18:3(OH)_22:5(OO))
$C_{45}H_{76}NO_{11}P$	[M+H]+	838.5211	838.5228	OxPE(18:1_22:6(OOO))
$C_{40}H_{74}O_7$	[M+NH4]+	684.5733	684.5772	OxTG(14:0_16:0_7:0(CHO))
$C_{46}H_{80}O_8$	[M+NH4]+	778.6174	778.6191	OxTG(16:1_18:2_9:0(COOH))
$C_{46}H_{82}O_8$	[M+NH4]+	780.6365	780.6348	OxTG(16:0_18:2_9:0(COOH))
$C_{47}H_{86}O_7$	[M+NH4]+	780.6678	780.6711	OxTG(16:0_18:1_10:0(CHO))
$C_{46}H_{84}O_8$	[M+NH4]+	782.6529	782.6504	OxTG(16:0_18:1_9:0(COOH))
$C_{47}H_{82}O_8$	[M+NH4]+	792.6342	792.6348	OxTG(18:1_18:2_8:0(COOH))
$C_{48}H_{88}O_7$	[M+NH4]+	794.6858	794.6868	OxTG(16:1_18:0_11:0(CHO))
$C_{48}H_{84}O_8$	[M+NH4]+	806.6530	806.6504	OxTG(18:1_18:2_9:0(COOH))
$C_{49}H_{90}O_7$	[M+NH4]+	808.7035	808.7024	OxTG(14:0_16:0_16:1(CHO))
$C_{50}H_{88}O_7$	[M+NH4]+	818.6853	818.6868	OxTG(18:1_18:1_11:1(CHO))
$C_{51}H_{94}O_7$	[M+NH4]+	836.7337	836.7337	OxTG(16:0_16:1_16:1(OH))
$C_{51}H_{96}O_7$	[M+NH4]+	838.7480	838.7494	OxTG(14:0_16:0_18:1(OH))
$C_{53}H_{98}O_7$	[M+NH4]+	864.7665	864.7650	OxTG(16:0_16:0_18:2(OH))
$C_{55}H_{98}O_7$	[M+NH4]+	888.7679	888.7650	OxTG(16:0_18:2_18:2(OH))
$C_{55}H_{100}O_7$	[M+NH4]+	890.7809	890.7807	OxTG(16:0_18:1_18:2(OH))
$C_{55}H_{102}O_7$	[M+NH4]+	892.7979	892.7963	OxTG(16:0_18:1_18:1(OH))
$C_{55}H_{98}O_8$	[M+NH4]+	904.7600	904.7600	OxTG(16:0_18:1_18:3(OO))
$C_{55}H_{100}O_8$	[M+NH4]+	906.7759	906.7756	OxTG(16:0_18:1_18:2(OOH))
$C_{56}H_{94}O_8$	[M+NH4]+	912.7288	912.7287	OxTG(22:3(OH)_17:2(Ke)_14:1)
$C_{57}H_{98}O_7$	[M+NH4]+	912.7702	912.7650	OxTG(18:1_18:3_18:1(Ke))
$C_{57}H_{100}O_7$	[M+NH4]+	914.7849	914.7807	OxTG(18:1_18:1_18:3(OH))
$C_{57}H_{104}O_7$	[M+NH4]+	918.8115	918.8120	OxTG(16:0_18:0_20:3(OH))
$C_{57}H_{108}O_7$	[M+NH4]+	922.8393	922.8433	OxTG(18:0_18:0_18:1(OH))
$C_{57}H_{100}O_8$	[M+NH4]+	930.7766	930.7756	OxTG(18:1_18:1_18:3(OO))
$C_{57}H_{102}O_8$	[M+NH4]+	932.7901	932.7912	OxTG(18:1_18:1_18:2(OOH))
$C_{34}H_{66}NO_8P$	[M+H]+	648.4614	648.4599	PC(8:0_18:1)
$C_{34}H_{68}NO_8P$	[M+H]+	650.4761	650.4755	PC(12:0_14:0)
$C_{36}H_{72}NO_8P$	[M+H]+	678.5089	678.5068	PC(14:0_14:0)
$C_{37}H_{72}NO_8P$	[M+H]+	690.5081	690.5068	PC(13:0_16:1)
$C_{37}H_{74}NO_8P$	[M+H]+	692.5237	692.5225	PC(14:0_15:0)

$C_{38}H_{74}NO_8P$	[M+H]+	704.5236	704.5225	PC(14:0_16:1)
$C_{38}H_{76}NO_8P$	[M+H]+	706.5387	706.5381	PC(14:0_16:0)
$C_{39}H_{78}NO_8P$	[M+H]+	720.5541	720.5538	PC(15:0_16:0)
$C_{40}H_{74}NO_8P$	[M+H]+	728.5230	728.5225	PC(14:0_18:3)
$C_{40}H_{76}NO_8P$	[M+H]+	730.5392	730.5381	PC(14:0_18:2)
$C_{40}H_{80}NO_8P$	[M+H]+	734.5691	734.5694	PC(14:0_18:0)
$C_{41}H_{80}NO_8P$	[M+H]+	746.5701	746.5694	PC(15:0_18:1)
$C_{41}H_{82}NO_8P$	[M+H]+	748.5858	748.5850	PC(15:0_18:0)
$C_{42}H_{76}NO_8P$	[M+H]+	754.5386	754.5381	PC(14:0_20:4)
$C_{42}H_{78}NO_8P$	[M+H]+	756.5538	756.5538	PC(16:0_18:3)
$C_{42}H_{80}NO_8P$	[M+H]+	758.5704	758.5694	PC(16:0_18:2)
$C_{42}H_{84}NO_8P$	[M+H]+	762.5984	762.6007	PC(16:0_18:0)
$C_{43}H_{82}NO_8P$	[M+H]+ | [M+Na]+	772.5849	772.5850	PC(17:1_18:1)
$C_{43}H_{84}NO_8P$	[M+H]+ | [M+Na]+	774.6014	774.6007	PC(17:0_18:1)
$C_{43}H_{86}NO_8P$	[M+H]+	776.6174	776.6163	PC(17:0_18:0)
$C_{44}H_{80}NO_8P$	[M+H]+	782.5683	782.5694	PC(18:2_18:2)
$C_{44}H_{82}NO_8P$	[M+H]+	784.5859	784.5850	PC(18:0_18:3)
$C_{44}H_{84}NO_8P$	[M+H]+	786.6011	786.6007	PC(18:1_18:1)
$C_{45}H_{80}NO_8P$	[M+H]+	794.5693	794.5694	PC(17:0_20:5)
$C_{45}H_{82}NO_8P$	[M+H]+	796.5833	796.5850	PC(17:0_20:4)
$C_{45}H_{84}NO_8P$	[M+H]+	798.6001	798.6007	PC(18:3_19:0)
$C_{45}H_{86}NO_8P$	[M+H]+ | [M+Na]+	800.6168	800.6163	PC(18:2_19:0)
$C_{46}H_{76}NO_8P$	[M+H]+	802.5340	802.5381	PC(18:3_20:5)
$C_{46}H_{78}NO_8P$	[M+H]+	804.5551	804.5538	PC(18:2_20:5)
$C_{46}H_{80}NO_8P$	[M+H]+ | [M+Na]+	806.5696	806.5694	PC(18:1_20:5)
$C_{46}H_{82}NO_8P$	[M+H]+ | [M+Na]+	808.5854	808.5850	PC(18:1_20:4)
$C_{46}H_{84}NO_8P$	[M+H]+	810.6005	810.6007	PC(18:0_20:4)
$C_{46}H_{86}NO_8P$	[M+H]+	812.6168	812.6163	PC(18:1_20:2)
$C_{46}H_{92}NO_8P$	[M+H]+	818.6608	818.6633	PC(18:0_20:0)
$C_{47}H_{86}NO_8P$	[M+H]+	824.6191	824.6163	PC(19:0_20:4)
$C_{48}H_{88}NO_8P$	[M+H]+	838.6338	838.6320	PC(20:0_20:4)
$C_{48}H_{94}NO_8P$	[M+H]+	844.6810	844.6789	PC(18:1_22:0)
$C_{50}H_{96}NO_8P$	[M+H]+	870.6968	870.6946	PC(18:2_24:0)
$C_{50}H_{98}NO_8P$	[M+H]+	872.7123	872.7102	PC(18:1_24:0)
$C_{52}H_{96}NO_8P$	[M+H]+	894.6965	894.6946	PC(20:4_24:0)
$C_{52}H_{102}NO_8P$	[M+H]+	900.7411	900.7415	PC(18:1_26:0)
$C_{31}H_{54}NO_8P$	[M+H]+	600.3667	600.3660	PE(26:4)
$C_{31}H_{60}NO_8P$	[M+H]+	606.4153	606.4129	PE(26:1)
$C_{33}H_{58}NO_8P$	[M+H]+	628.3988	628.3973	PE(28:4)
$C_{34}H_{64}NO_8P$	[M+H]+	646.4457	646.4442	PE(29:2)
$C_{35}H_{64}NO_8P$	[M+H]+	658.4434	658.4442	PE(30:3)
$C_{35}H_{66}NO_8P$	[M+H]+	660.4597	660.4599	PE(30:2)
$C_{37}H_{70}NO_8P$	[M+H]+	688.4930	688.4912	PE(14:1_18:1)
$C_{38}H_{72}NO_8P$	[M+H]+	702.5056	702.5068	PE(15:0_18:2)
$C_{39}H_{72}NO_8P$	[M+H]+	714.5077	714.5068	PE(16:0_18:3)
$C_{39}H_{76}NO_8P$	[M+H]+	718.5390	718.5381	PE(16:0_18:1)
$C_{41}H_{72}NO_8P$	[M+H]+	738.5068	738.5068	PE(16:0_20:5)
$C_{41}H_{74}NO_8P$	[M+H]+	740.5235	740.5225	PE(16:0_20:4)
$C_{41}H_{76}NO_8P$	[M+H]+ | [M+Na]+	742.5387	742.5381	PE(18:1_18:2)
$C_{41}H_{78}NO_8P$	[M+H]+	744.5540	744.5538	PE(18:1_18:1)

$C_{43}H_{74}NO_8P$	[M+H]+	764.5232	764.5225	PE(18:1_20:5)
$C_{43}H_{76}NO_8P$	[M+H]+	766.5389	766.5381	PE(18:1_20:4)
$C_{43}H_{78}NO_8P$	[M+H]+	768.5541	768.5538	PE(18:0_20:4)
$C_{39}H_{77}O_{10}P$	[M+NH4]+	754.5544	754.5592	PG(14:0_19:0)
$C_{42}H_{79}O_{10}P$	[M+H]+	775.5514	775.5483	PG(18:1_18:1)
$C_{45}H_{85}O_{13}P$	[M+NH4]+	882.6079	882.6066	PI(36:1)
$C_{40}H_{74}NO_{10}P$	[M+H]+	760.5127	760.5123	PS(16:0_18:2)
$C_{40}H_{76}NO_{10}P$	[M+H]+	762.5321	762.5279	PS(16:0_18:1)
$C_{41}H_{74}NO_{10}P$	[M+H]+	772.5146	772.5123	PS(17:2_18:1)
$C_{41}H_{76}NO_{10}P$	[M+H]+	774.5325	774.5279	PS(17:0_18:2)
$C_{42}H_{72}NO_{10}P$	[M+H]+	782.4980	782.4966	PS(16:0_20:5)
$C_{42}H_{74}NO_{10}P$	[M+H]+	784.5140	784.5123	PS(18:1_18:3)
$C_{42}H_{76}NO_{10}P$	[M+H]+	786.5296	786.5279	PS(18:1_18:2)
$C_{42}H_{80}NO_{10}P$	[M+H]+	790.5626	790.5592	PS(18:0_18:1)
$C_{44}H_{76}NO_{10}P$	[M+H]+	810.5277	810.5279	PS(18:1_20:4)
$C_{44}H_{78}NO_{10}P$	[M+H]+	812.5439	812.5436	PS(18:0_20:4)
$C_{44}H_{80}NO_{10}P$	[M+H]+	814.5603	814.5592	PS(18:0_20:3)
$C_{34}H_{71}N_2O_6P$	[M+H]+	635.5126	635.5122	SM(d16:0/13:0)
$C_{35}H_{71}N_2O_6P$	[M+H]+	647.5136	647.5122	SM(d14:1/16:0)
$C_{36}H_{73}N_2O_6P$	[M+H]+	661.5290	661.5279	SM(d18:1/13:0)
$C_{36}H_{75}N_2O_6P$	[M+H]+	663.5437	663.5435	SM(d18:0/13:0)
$C_{37}H_{69}N_2O_6P$	[M+H]+	669.4964	669.4966	SM(d14:1/18:3)
$C_{37}H_{73}N_2O_6P$	[M+H]+	673.5286	673.5279	SM(d20:2/12:0)
$C_{37}H_{75}N_2O_6P$	[M+H]+	675.5444	675.5435	SM(d16:1/16:0)
$C_{38}H_{71}N_2O_6P$	[M+H]+	683.5131	683.5122	SM(d20:4/13:0)
$C_{38}H_{73}N_2O_6P$	[M+H]+	685.5282	685.5279	SM(d20:3/13:0)
$C_{38}H_{77}N_2O_6P$	[M+H]+	689.5587	689.5592	SM(d16:1/17:0)
$C_{38}H_{79}N_2O_6P$	[M+H]+	691.5754	691.5748	SM(d16:0/17:0)
$C_{39}H_{73}N_2O_6P$	[M+H]+	697.5264	697.5279	SM(d16:1/18:3)
$C_{39}H_{77}N_2O_6P$	[M+H]+	701.5602	701.5592	SM(d18:2/16:0)
$C_{39}H_{79}N_2O_6P$	[M+H]+	703.5756	703.5748	SM(d16:1/18:0)
$C_{39}H_{81}N_2O_6P$	[M+H]+	705.5909	705.5905	SM(d18:0/16:0)
$C_{40}H_{73}N_2O_6P$	[M+H]+	709.5266	709.5279	SM(d22:5/13:0)
$C_{40}H_{75}N_2O_6P$	[M+H]+	711.5415	711.5435	SM(d15:0/20:4)
$C_{40}H_{79}N_2O_6P$	[M+H]+	715.5757	715.5748	SM(d20:2/15:0)
$C_{41}H_{75}N_2O_6P$	[M+H]+	723.5436	723.5435	SM(d18:2/18:3)
$C_{41}H_{77}N_2O_6P$	[M+H]+	725.5582	725.5592	SM(d16:1/20:3)
$C_{41}H_{79}N_2O_6P$	[M+H]+	727.5758	727.5748	SM(d20:3/16:0)
$C_{41}H_{81}N_2O_6P$	[M+H]+	729.5916	729.5905	SM(d20:2/16:0)
$C_{41}H_{83}N_2O_6P$	[M+H]+	731.6064	731.6061	SM(d16:1/20:0)
$C_{42}H_{79}N_2O_6P$	[M+H]+	739.5734	739.5748	SM(d15:0/22:4)
$C_{42}H_{81}N_2O_6P$	[M+H]+	741.5920	741.5905	SM(d20:3/17:0)
$C_{42}H_{83}N_2O_6P$	[M+H]+	743.6078	743.6061	SM(d17:1/20:1)
$C_{42}H_{85}N_2O_6P$	[M+H]+	745.6239	745.6218	SM(d18:1/19:0)
$C_{43}H_{77}N_2O_6P$	[M+H]+	749.5608	749.5592	SM(d20:3/18:3)
$C_{43}H_{79}N_2O_6P$	[M+H]+	751.5746	751.5748	SM(d20:1/18:4)
$C_{43}H_{81}N_2O_6P$	[M+H]+	753.5894	753.5905	SM(d20:3/18:1)
$C_{43}H_{83}N_2O_6P$	[M+H]+	755.6078	755.6061	SM(d18:1/20:2)
$C_{43}H_{85}N_2O_6P$	[M+H]+	757.6233	757.6218	SM(d18:1/20:1)
$C_{43}H_{87}N_2O_6P$	[M+H]+	759.6397	759.6374	SM(d16:1/22:0)

$C_{44}H_{83}N_2O_6P$	[M+H]+	767.6067	767.6061	SM(d22:2/17:2)
$C_{44}H_{87}N_2O_6P$	[M+H]+	771.6405	771.6374	SM(d20:2/19:0)
$C_{44}H_{89}N_2O_6P$	[M+H]+	773.6562	773.6531	SM(d16:1/23:0)
$C_{45}H_{83}N_2O_6P$	[M+H]+	779.6060	779.6061	SM(d20:2/20:3)
$C_{45}H_{85}N_2O_6P$	[M+H]+	781.6211	781.6218	SM(d20:2/20:2)
$C_{45}H_{87}N_2O_6P$	[M+H]+	783.6413	783.6374	SM(d20:3/20:0)
$C_{45}H_{89}N_2O_6P$	[M+H]+	785.6551	785.6531	SM(d20:2/20:0)
$C_{45}H_{91}N_2O_6P$	[M+H]+	787.6701	787.6687	SM(d18:1/22:0)
$C_{46}H_{91}N_2O_6P$	[M+H]+	799.6712	799.6687	SM(d20:2/21:0)
$C_{47}H_{87}N_2O_6P$	[M+H]+	807.6356	807.6374	SM(d20:2/22:3)
$C_{47}H_{93}N_2O_6P$	[M+H]+	813.6860	813.6844	SM(d20:2/22:0)
$C_{47}H_{95}N_2O_6P$	[M+H]+	815.7012	815.7000	SM(d18:1/24:0)
$C_{48}H_{93}N_2O_6P$	[M+H]+	825.6880	825.6844	SM(d20:3/23:0)
$C_{48}H_{95}N_2O_6P$	[M+H]+	827.7022	827.7000	SM(d20:2/23:0)
$C_{49}H_{97}N_2O_6P$	[M+H]+	841.7180	841.7157	SM(d20:2/24:0)
$C_{51}H_{103}N_2O_6P$	[M+H]+	871.7646	871.7626	SM(d20:1/26:0)
$C_{15}H_{31}NO_2$	[M+H]+	258.2429	258.2427	So(d15:1)
$C_{16}H_{33}NO_2$	[M+H]+	272.2584	272.2584	So(d16:1)
$C_{16}H_{35}NO_2$	[M+H]+	274.2741	274.2740	So(d16:0)
$C_{17}H_{35}NO_2$	[M+H]+	286.2744	286.2740	So(d17:1)
$C_{18}H_{35}NO_2$	[M+H]+	298.2744	298.2740	So(d18:2)
$C_{18}H_{37}NO_2$	[M+H]+	300.2900	300.2897	So(d18:1)
$C_{18}H_{39}NO_2$	[M+H]+	302.3057	302.3053	So(d18:0)
$C_{42}H_{80}O_{12}S$	[M+NH4]+	826.5731	826.5709	SQDG(15:0_18:0)
$C_{33}H_{62}O_6$	[M+NH4]+	572.4895	572.4884	TG(10:0_12:0_8:0)
$C_{35}H_{66}O_6$	[M+NH4]+|[M+Na]+	600.5206	600.5197	TG(12:0_12:0_8:0)
$C_{37}H_{68}O_6$	[M+NH4]+	626.5365	626.5354	TG(18:1_8:0_8:0)
$C_{37}H_{70}O_6$	[M+NH4]+	628.5522	628.5510	TG(12:0_14:0_8:0)
$C_{39}H_{72}O_6$	[M+NH4]+	654.5685	654.5667	TG(10:0_18:1_8:0)
$C_{39}H_{74}O_6$	[M+NH4]+|[M+Na]+	656.5830	656.5823	TG(12:0_16:0_8:0)
$C_{40}H_{76}O_6$	[M+NH4]+	670.5982	670.5980	TG(13:0_16:0_8:0)
$C_{41}H_{76}O_6$	[M+NH4]+	682.5989	682.5980	TG(10:0_10:0_18:1)
$C_{42}H_{80}O_6$	[M+NH4]+	698.6306	698.6293	TG(15:0_16:0_8:0)
$C_{43}H_{78}O_6$	[M+NH4]+	708.6166	708.6136	TG(14:1_18:1_8:0)
$C_{43}H_{80}O_6$	[M+NH4]+|[M+Na]+	710.6308	710.6293	TG(10:0_12:0_18:1)
$C_{44}H_{82}O_6$	[M+NH4]+	724.6452	724.6449	TG(15:0_18:1_8:0)
$C_{44}H_{84}O_6$	[M+NH4]+	726.6619	726.6606	TG(10:0_15:0_16:0)
$C_{45}H_{82}O_6$	[M+NH4]+|[M+Na]+	736.6466	736.6449	TG(16:1_18:1_8:0)
$C_{46}H_{86}O_6$	[M+NH4]+	752.6776	752.6762	TG(13:0_14:0_16:1)
$C_{47}H_{88}O_6$	[M+NH4]+	766.6903	766.6919	TG(10:0_16:0_18:1)
$C_{51}H_{90}O_6$	[M+NH4]+	816.7037	816.7075	TG(14:1_16:1_18:2)
$C_{51}H_{98}O_6$	[M+NH4]+	824.7707	824.7701	TG(14:0_16:0_18:0)
$C_{53}H_{98}O_6$	[M+NH4]+	848.7715	848.7701	TG(16:0_16:1_18:1)
$C_{54}H_{98}O_6$	[M+NH4]+	860.7712	860.7701	TG(15:0_18:1_18:2)
$C_{55}H_{98}O_6$	[M+NH4]+	872.7720	872.7701	TG(16:0_18:2_18:2)
$C_{55}H_{100}O_6$	[M+NH4]+	874.7874	874.7858	TG(16:0_18:1_18:2)
$C_{56}H_{98}O_6$	[M+NH4]+	884.7734	884.7701	TG(17:1_18:2_18:2)
$C_{57}H_{92}O_6$	[M+NH4]+	890.7264	890.7232	TG(18:2_18:3_18:4)
$C_{57}H_{94}O_6$	[M+NH4]+	892.7401	892.7388	TG(18:2_18:3_18:3)
$C_{56}H_{108}O_6$	[M+NH4]+	894.8487	894.8484	TG(16:0_18:0_19:0)

$C_{57}H_{100}O_6$	[M+NH4]+	898.7867	898.7858	TG(18:1_18:2_18:2)
$C_{58}H_{106}O_6$	[M+NH4]+	916.8323	916.8327	TG(18:1_18:2_19:0)
$C_{58}H_{108}O_6$	[M+NH4]+	918.8488	918.8484	TG(16:1_18:1_21:0)
$C_{59}H_{100}O_6$	[M+NH4]+	922.7865	922.7858	TG(18:1_18:2_20:4)
$C_{59}H_{102}O_6$	[M+NH4]+	924.8019	924.8014	TG(18:1_18:1_20:4)
$C_{60}H_{112}O_6$	[M+NH4]+	946.8810	946.8797	TG(16:1_18:1_23:0)
$C_{60}H_{114}O_6$	[M+NH4]+	948.8943	948.8953	TG(16:0_16:1_25:0)
$C_{62}H_{116}O_6$	[M+NH4]+	974.9137	974.9110	TG(16:1_18:1_25:0)
$C_{63}H_{114}O_6$	[M+NH4]+	984.8988	984.8953	TG(18:2_18:2_24:0)
$C_{63}H_{116}O_6$	[M+NH4]+	986.9144	986.9110	TG(18:1_18:2_24:0)
$C_{65}H_{120}O_6$	[M+NH4]+	1014.9471	1014.9423	TG(18:1_18:2_26:0)

Table S4. List of all top-ranked identified lipids from *Hottentotta saulcyi* venom in negative-ion mode. The data obtained by LipidMatch software and the formula of the neutral lipids as well as the theoretical m/z values were obtained by using the LipidPioneer template.[1].

Formula of Neutral Lipid	Adducts Confirmed	Experimental *m/z*	Theoretical *m/z*	Lipid
$C_{34}H_{67}NO_3$	[M-H]-	536.5027	536.5048	Cer(d18:1/16:0)
$C_{34}H_{67}NO_4$	[M-H]-	552.4973	552.4997	Cer(t19:1/15:0)
$C_{32}H_{63}NO_3$	[M+HCO2]-|[M-H]-	554.4767	554.4790	Cer(d16:1/16:0)
$C_{34}H_{69}NO_4$	[M-H]-	554.5131	554.5154	Cer(t18:0/16:0)
$C_{36}H_{59}NO_4$	[M-H]-	568.4395	568.4371	Cer(d17:2/h19:5)
$C_{33}H_{65}NO_3$	[M+HCO2]-	568.4934	568.4946	Cer(d17:1/16:0)
$C_{34}H_{65}NO_3$	[M+HCO2]-	580.4926	580.4946	Cer(d18:2/16:0)
$C_{36}H_{71}NO_4$	[M-H]-	580.5293	580.5310	Cer(d20:1/h16:0)
$C_{34}H_{69}NO_3$	[M+HCO2]-	584.5234	584.5259	Cer(d18:0/16:0)
$C_{38}H_{75}NO_3$	[M-H]-	592.5657	592.5674	Cer(d16:1/22:0)
$C_{34}H_{67}NO_4$	[M+HCO2]-	598.5023	598.5052	Cer(d16:0/h18:1)
$C_{36}H_{67}NO_3$	[M+HCO2]-	606.5082	606.5103	Cer(d20:3/16:0)
$C_{36}H_{69}NO_3$	[M+HCO2]-|[M-H]-	608.5245	608.5259	Cer(d20:2/16:0)
$C_{36}H_{71}NO_3$	[M+HCO2]-|[M-H]-	610.5379	610.5416	Cer(d16:1/20:0)
$C_{40}H_{79}NO_3$	[M-H]-	620.5969	620.5987	Cer(d18:1/22:0)
$C_{37}H_{73}NO_3$	[M+HCO2]-	624.5555	624.5572	Cer(d15:1/22:0)
$C_{38}H_{73}NO_3$	[M+HCO2]-	636.5557	636.5572	Cer(d16:2/22:0)
$C_{38}H_{75}NO_3$	[M+HCO2]-|[M-H]-	638.5699	638.5729	Cer(d16:1/22:0)
$C_{38}H_{77}NO_3$	[M+HCO2]-	640.5860	640.5885	Cer(d16:0/22:0)
$C_{39}H_{77}NO_3$	[M+HCO2]-	652.5870	652.5885	Cer(d16:1/23:0)
$C_{38}H_{75}NO_4$	[M+HCO2]-	654.5657	654.5678	Cer(t16:1/22:0)
$C_{40}H_{77}NO_3$	[M+HCO2]-	664.5854	664.5885	Cer(d20:2/20:0)
$C_{43}H_{85}NO_4$	[M-H]-	678.6395	678.6406	Cer(d17:1/h26:0)
$C_{40}H_{77}NO_4$	[M+HCO2]-	680.5833	680.5834	Cer(t25:2/15:0)
$C_{41}H_{81}NO_3$	[M+HCO2]-	680.6185	680.6198	Cer(d18:1/23:0)
$C_{40}H_{79}NO_4$	[M+HCO2]-	682.5956	682.5991	Cer(t18:1/22:0)
$C_{42}H_{85}NO_5$	[M-H]-	682.6332	682.6355	Cer(t26:0/h16:0)
$C_{40}H_{81}NO_4$	[M+HCO2]-|[M-H]-	684.6120	684.6147	Cer(t18:0/22:0)
$C_{42}H_{79}NO_3$	[M+HCO2]-	690.6025	690.6042	Cer(d20:3/22:0)
$C_{42}H_{81}NO_3$	[M+HCO2]-|[M-H]-	692.6180	692.6198	Cer(d20:2/22:0)
$C_{44}H_{87}NO_4$	[M-H]-	692.6554	692.6562	Cer(d20:1/h24:0)
$C_{42}H_{83}NO_3$	[M+HCO2]-	694.6339	694.6355	Cer(d18:1/24:0)
$C_{42}H_{85}NO_3$	[M+HCO2]-	696.6494	696.6511	Cer(d18:0/24:0)

$C_{42}H_{81}NO_4$	[M+HCO2]-	708.6142	708.6147	Cer(t27:2/15:0)
$C_{44}H_{87}NO_5$	[M-H]-	708.6497	708.6511	Cer(t18:0/h26:1)
$C_{42}H_{83}NO_4$	[M+HCO2]-	710.6283	710.6304	Cer(t16:1/26:0)
$C_{44}H_{89}NO_5$	[M-H]-	710.6647	710.6668	Cer(t22:0/h22:0)
$C_{42}H_{85}NO_4$	[M+HCO2]-丨[M-H]-	712.6436	712.6460	Cer(t18:0/24:0)
$C_{44}H_{83}NO_3$	[M+HCO2]-	718.6333	718.6355	Cer(d22:3/22:0)
$C_{44}H_{85}NO_3$	[M+HCO2]-	720.6509	720.6511	Cer(d20:2/24:0)
$C_{46}H_{91}NO_4$	[M-H]-	720.6869	720.6875	Cer(d28:1/h18:0)
$C_{46}H_{93}NO_4$	[M-H]-	722.7020	722.7031	Cer(t22:0/24:0)
$C_{43}H_{85}NO_4$	[M+HCO2]-	724.6438	724.6460	Cer(t19:1/24:0)
$C_{45}H_{91}NO_5$	[M-H]-	724.6812	724.6824	Cer(t19:0/h26:0)
$C_{43}H_{87}NO_4$	[M+HCO2]-	726.6590	726.6617	Cer(t17:0/26:0)
$C_{45}H_{89}NO_3$	[M+HCO2]-	736.6807	736.6824	Cer(d19:1/26:0)
$C_{44}H_{87}NO_4$	[M+HCO2]-	738.6611	738.6617	Cer(d26:1/h18:0)
$C_{46}H_{93}NO_5$	[M-H]-	738.6969	738.6981	Cer(t26:0/h20:0)
$C_{44}H_{89}NO_4$	[M+HCO2]-	740.6760	740.6773	Cer(t18:0/26:0)
$C_{44}H_{87}NO_5$	[M+HCO2]-	754.6560	754.6566	Cer(t18:1/h26:0)
$C_{45}H_{91}NO_4$	[M+HCO2]-	754.6911	754.6930	Cer(t21:0/24:0)
$C_{44}H_{89}NO_5$	[M+HCO2]-	756.6694	756.6723	Cer(t20:0/h24:0)
$C_{46}H_{91}NO_4$	[M+HCO2]-丨[M-H]-	766.6920	766.6930	Cer(t20:1/26:0)
$C_{45}H_{91}NO_5$	[M+HCO2]-	770.6866	770.6879	Cer(t20:0/h25:0)
$C_{48}H_{95}NO_3$	[M+HCO2]-	778.7280	778.7294	Cer(d20:1/28:0)
$C_{47}H_{95}NO_4$	[M+HCO2]-	782.7237	782.7243	Cer(t22:0/25:0)
$C_{46}H_{93}NO_5$	[M+HCO2]-	784.7029	784.7036	Cer(t22:0/h24:0)
$C_{49}H_{97}NO_3$	[M+HCO2]-	792.7450	792.7450	Cer(d21:1/28:0)
$C_{48}H_{95}NO_4$	[M+HCO2]-	794.7225	794.7243	Cer(d28:1/h20:0)
$C_{48}H_{97}NO_4$	[M+HCO2]-	796.7395	796.7399	Cer(t22:0/26:0)
$C_{50}H_{101}NO_4$	[M+HCO2]-	824.7721	824.77124	Cer(t22:0/28:0)
$C_{75}H_{140}O_{17}P_2$	[M-2H]2-	686.4753	686.4710	CL(16:0_16:0_16:1_18:2)
$C_{77}H_{148}O_{17}P_2$	[M-2H]2-	702.5065	702.5023	CL(16:0_16:0_18:0_18:1)
$C_{79}H_{144}O_{17}P_2$	[M-2H]2-	712.4891	712.4866	CL(16:1_18:1_18:1_18:2)
$C_{79}H_{148}O_{17}P_2$	[M-2H]2-	714.5058	714.5023	CL(16:0_18:1_18:1_18:1)
$C_{81}H_{156}O_{17}P_2$	[M-2H]2-	730.5378	730.5336	CL(18:0_18:0_18:0_18:1)
$C_{83}H_{156}O_{17}P_2$	[M-2H]2-	742.5383	742.5336	CL(18:0_18:0_18:3_20:0)
$C_{83}H_{160}O_{17}P_2$	[M-2H]2-	744.5534	744.5492	CL(16:0_18:0_20:0_20:1)
$C_{85}H_{160}O_{17}P_2$	[M-2H]2-	756.5529	756.5492	CL(18:0_18:0_20:1_20:2)
$C_{87}H_{144}O_{17}P_2$	[M-2H]2-	760.4833	760.4866	CL(18:1_20:4_20:4_20:4)
$C_{87}H_{158}O_{17}P_2$	[M-2H]2-	767.5415	767.5414	CL(18:0_20:1_20:1_20:4)
$C_{87}H_{164}O_{17}P_2$	[M-2H]2-	770.5693	770.5649	CL(18:2_20:0_20:1_20:0)
$C_{37}H_{72}NO_8P$	[M-H]-	688.4908	688.4923	DMPE(14:0_16:1)
$C_{41}H_{76}NO_8P$	[M-H]-	740.5215	740.5236	DMPE(16:0_18:3)
$C_{43}H_{78}NO_8P$	[M-H]-	766.5363	766.5392	DMPE(18:2_18:2)
$C_{43}H_{84}NO_8P$	[M-H]-	772.5849	772.5861	DMPE(18:0_18:1)
$C_{49}H_{92}NO_8P$	[M-H]-	852.6515	852.6487	DMPE(20:3_22:0)
$C_{26}H_{52}NO_6P$	[M+HCO2]-	550.3502	550.3514	Ether-LPC(P-18:1)
$C_{26}H_{54}NO_6P$	[M+HCO2]-	552.3657	552.3671	Ether-LPC(P-18:0)
$C_{41}H_{82}NO_7P$	[M+HCO2]-	776.5824	776.5811	Ether-PC(O-16:0/17:1)
$C_{42}H_{82}NO_7P$	[M+HCO2]-	788.5816	788.5811	Ether-PC(O-18:1/16:1)
$C_{44}H_{78}NO_7P$	[M+HCO2]-	808.5484	808.5498	Ether-PC(P-18:1/18:4)
$C_{44}H_{80}NO_7P$	[M+HCO2]-	810.5642	810.5654	Ether-PC(P-18:1/18:3)

$C_{44}H_{82}NO_7P$	[M+HCO2]-	812.5797	812.5811	Ether-PC(P-16:0/20:3)
$C_{44}H_{84}NO_7P$	[M+HCO2]-	814.5956	814.5967	Ether-PC(O-16:1/20:2)
$C_{44}H_{86}NO_7P$	[M+HCO2]-	816.6108	816.6124	Ether-PC(O-18:0/18:2)
$C_{44}H_{88}NO_7P$	[M+HCO2]-	818.6266	818.6280	Ether-PC(O-20:1/16:0)
$C_{46}H_{84}NO_7P$	[M+HCO2]-	838.5956	838.5967	Ether-PC(P-18:1/20:3)
$C_{46}H_{86}NO_7P$	[M+HCO2]-	840.6102	840.6124	Ether-PC(O-18:1/20:3)
$C_{38}H_{78}NO_7P$	[M-H]-	690.5493	690.5443	Ether-PE(O-18:0/15:0)
$C_{39}H_{74}NO_7P$	[M-H]-	698.5112	698.5130	Ether-PE(O-16:0/18:3)
$C_{39}H_{76}NO_7P$	[M-H]-	700.5267	700.5286	Ether-PE(O-16:0/18:2)
$C_{39}H_{78}NO_7P$	[M-H]-	702.5401	702.5443	Ether-PE(P-18:0/16:0)
$C_{40}H_{76}NO_7P$	[M-H]-	712.5266	712.5286	Ether-PE(P-18:1/17:1)
$C_{41}H_{72}NO_7P$	[M-H]-	720.4956	720.4973	Ether-PE(P-16:0/20:5)
$C_{41}H_{74}NO_7P$	[M-H]-	722.5115	722.5130	Ether-PE(O-16:0/20:5)
$C_{41}H_{78}NO_7P$	[M-H]-	726.5426	726.5443	Ether-PE(O-18:0/18:3)
$C_{41}H_{80}NO_7P$	[M-H]-	728.5581	728.5599	Ether-PE(P-20:0/16:1)
$C_{41}H_{82}NO_7P$	[M-H]-	730.5732	730.5756	Ether-PE(P-18:0/18:0)
$C_{43}H_{74}NO_7P$	[M-H]-	746.5110	746.5130	Ether-PE(P-18:1/20:5)
$C_{43}H_{76}NO_7P$	[M-H]-	748.5239	748.5286	Ether-PE(P-18:1/20:4)
$C_{43}H_{78}NO_7P$	[M-H]-	750.5422	750.5443	Ether-PE(O-18:0/20:5)
$C_{43}H_{82}NO_7P$	[M-H]-	754.5732	754.5756	Ether-PE(O-20:0/18:3)
$C_{43}H_{84}NO_7P$	[M-H]-	756.5892	756.5912	Ether-PE(O-20:0/18:2)
$C_{43}H_{86}NO_7P$	[M-H]-	758.6034	758.6069	Ether-PE(O-18:0/20:1)
$C_{32}H_{60}NO_9P$	[M-H]-	632.3916	632.3933	Ether-PS(P-16:1/10:0)
$C_{32}H_{62}NO_9P$	[M-H]-	634.4073	634.4089	Ether-PS(P-16:0/10:0)
$C_{42}H_{74}NO_9P$	[M-H]-	766.5018	766.5028	Ether-PS(P-18:0/18:4)
$C_{42}H_{76}NO_9P$	[M-H]-	768.5153	768.5185	Ether-PS(P-18:0/18:3)
$C_{43}H_{82}NO_9P$	[M-H]-	786.5643	786.5654	Ether-PS(P-18:1/19:0)
$C_{44}H_{76}NO_9P$	[M-H]-	792.5146	792.5185	Ether-PS(P-18:0/20:5)
$C_{44}H_{78}NO_9P$	[M-H]-	794.5299	794.5341	Ether-PS(P-16:0/22:4)
$C_{44}H_{80}NO_9P$	[M-H]-	796.5512	796.5498	Ether-PS(P-20:0/18:3)
$C_{44}H_{82}NO_9P$	[M-H]-	798.5651	798.5654	Ether-PS(P-20:0/18:2)
$C_{46}H_{78}NO_9P$	[M-H]-	818.5307	818.5341	Ether-PS(P-20:1/20:5)
$C_{18}H_{31}O_4$	[M-H]-	311.2218	311.2228	FAHFA(15:1/3:0)
$C_{18}H_{34}O_4$	[M-H]-	313.2368	313.2384	FAHFA(15:0/3:0)
$C_{19}H_{36}O_4$	[M-H]-	325.2372	325.2384	FAHFA(17:1/2:0)
$C_{19}H_{34}O_4$	[M-H]-	327.2531	327.2541	FAHFA(17:0/2:0)
$C_{21}H_{33}O_4$	[M-H]-	349.2406	349.2384	FAHFA(18:3/3:0)
$C_{38}H_{73}NO_8$	[M+HCO2]-	716.5280	716.5318	GlcCer(d16:1/16:0)
$C_{43}H_{83}NO_8$	[M+HCO2]-	786.6108	786.6101	GlcCer(d16:1/21:0)
$C_{44}H_{83}NO_8$	[M+HCO2]-	798.6094	798.6101	GlcCer(d16:2/22:0)
$C_{44}H_{85}NO_8$	[M+HCO2]-	800.6250	800.6257	GlcCer(d16:1/22:0)
$C_{46}H_{89}NO_8$	[M+HCO2]-	828.6541	828.6570	GlcCer(d18:1/22:0)
$C_{48}H_{91}NO_8$	[M+HCO2]-	854.6697	854.6727	GlcCer(d20:2/22:0)
$C_{22}H_{46}NO_7P$	[M+HCO2]-	512.2973	512.2994	LPC(14:0)
$C_{24}H_{50}NO_7P$	[M+HCO2]-	540.3288	540.3307	LPC(16:0)
$C_{25}H_{50}NO_7P$	[M+HCO2]-	552.3292	552.3307	LPC(17:1)
$C_{26}H_{50}NO_7P$	[M+HCO2]-	564.3285	564.3307	LPC(18:2)
$C_{26}H_{52}NO_7P$	[M+HCO2]-	566.3445	566.3463	LPC(18:1)
$C_{26}H_{54}NO_7P$	[M+HCO2]-	568.3605	568.3620	LPC(18:0)
$C_{27}H_{56}NO_7P$	[M+HCO2]-	582.3770	582.3776	LPC(19:0)

$C_{28}H_{56}NO_7P$	[M+HCO2]-	594.3739	594.3776	LPC(20:1)
$C_{29}H_{57}NO_9P$	[M+HCO2]-	596.3923	596.3933	LPC(20:0)
$C_{21}H_{44}NO_7P$	[M-H]-	452.2764	452.2782	LPE(16:0)
$C_{23}H_{44}NO_7P$	[M-H]-	476.2756	476.2782	LPE(18:2)
$C_{23}H_{46}NO_7P$	[M-H]-	478.2917	478.2939	LPE(18:1)
$C_{23}H_{48}NO_7P$	[M-H]-	480.3075	480.3095	LPE(18:0)
$C_{24}H_{50}NO_7P$	[M-H]-	494.3226	494.3252	LPE(19:0)
$C_{25}H_{42}NO_7P$	[M-H]-	498.2604	498.2626	LPE(20:5)
$C_{25}H_{44}NO_7P$	[M-H]-	500.2759	500.2782	LPE(20:4)
$C_{25}H_{50}NO_7P$	[M-H]-	506.3220	506.3252	LPE(20:1)
$C_{25}H_{52}NO_7P$	[M-H]-	508.3395	508.3408	LPE(20:0)
$C_{29}H_{60}NO_7P$	[M-H]-	564.4034	564.4034	LPE(24:0)
$C_{27}H_{53}O_{12}P$	[M-H]-	599.3179	599.3202	LPI(18:0)
$C_{41}H_{72}O_{10}$	[M+HCO2]-	769.5089	769.5107	MGDG(14:0_18:3)
$C_{42}H_{78}O_{10}$	[M+HCO2]-	787.5615	787.5577	MGDG(15:0_18:1)
$C_{42}H_{80}O_{10}$	[M+HCO2]-	789.5760	789.5733	MGDG(16:0_17:0)
$C_{43}H_{74}O_{10}$	[M+HCO2]-	795.5314	795.5264	MGDG(16:0_18:4)
$C_{45}H_{82}O_{10}$	[M+HCO2]-	827.5887	827.5890	MGDG(18:0_18:2)
$C_{40}H_{76}NO_8P$	[M-H]-	728.5224	728.5236	MMPE(17:1_17:1)
$C_{42}H_{76}NO_8P$	[M-H]-	752.5218	752.5236	MMPE(18:2_18:2)
$C_{42}H_{78}NO_8P$	[M-H]-	754.5374	754.5392	MMPE(16:0_20:3)
$C_{79}H_{146}O_{19}P_2$	[M-2H]2-	729.4904	729.4894	OxCL(16:0_18:1_18:1(OOH)_18:2)
$C_{77}H_{140}O_{21}P_2$	[M-2H]2-	730.4622	730.4608	OxCL(16:0_16:1(OH)_18:1(OOH)_18:3(OH))
$C_{81}H_{148}O_{19}P_2$	[M-2H]2-	742.5018	742.4972	OxCL(18:1_18:1_18:1(OOH)_18:2)
$C_{79}H_{146}O_{21}P_2$	[M-2H]2-	745.4854	745.4843	OxCL(16:0_18:1_18:1(OOH)_18:2(OOH))
$C_{81}H_{140}O_{20}P_2$	[M-2H]2-	746.4611	746.4634	OxCL(18:1_18:2(OH)_18:3(OH)_18:3(OH))
$C_{83}H_{146}O_{19}P_2$	[M-2H]2-	753.4864	753.4894	OxCL(18:1_18:2(OOH)_18:1_20:4)
$C_{81}H_{144}O_{21}P_2$	[M-2H]2-	756.4783	756.4765	OxCL(16:1_18:1(OOH)_18:1(OOH)_20:4)
$C_{85}H_{152}O_{18}P_2$	[M-2H]2-	760.5118	760.5154	OxCL(16:1(OH)_20:1_20:1_20:4)
$C_{85}H_{148}O_{19}P_2$	[M-2H]2-	766.4988	766.4972	OxCL(18:1(OH)_18:1(OH)_20:3_20:4)
$C_{83}H_{144}O_{21}P_2$	[M-2H]2-	768.4801	768.4765	OxCL(16:0_18:1_20:4(OOH)_20:4(OOH))
$C_{83}H_{148}O_{21}P_2$	[M-2H]2-	770.4958	770.4921	OxCL(18:1_18:1_18:1(OOH)_20:4(OOH))
$C_{85}H_{144}O_{21}P_2$	[M-2H]2-	780.4779	780.4765	OxCL(16:1_20:3(OOH)_20:3(OOH)_20:4)
$C_{81}H_{146}O_{24}P_2$	[M-2H]2-	781.4729	781.4767	OxCL(16:0_18:1(OOH)_18:2(OH)_20:3(OOH)2)
$C_{85}H_{146}O_{21}P_2$	[M-2H]2-	781.4831	781.4843	OxCL(18:1_18:2_20:3(OOH)_20:4(OOH))
$C_{85}H_{148}O_{21}P_2$	[M-2H]2-	782.4942	782.4921	OxCL(18:1_18:2(OOH)_20:3(OH)_20:3(OH))

$C_{83}H_{144}O_{23}P_2$	[M-2H]2-	784.4762	784.4714	OxCL(18:1_18:2(OOH)_18:3_20:3(OOH)2)
$C_{83}H_{148}O_{23}P_2$	[M-2H]2-	786.4907	786.4870	OxCL(18:1_18:1_18:2(OOH)_20:3(OOH)2)
$C_{83}H_{136}O_{24}P_2$	[M-2H]2-	788.4330	788.4375	OxCL(18:3_18:3(OH)_18:3(OOH)_20:4(OOH)2)
$C_{81}H_{144}O_{25}P_2$	[M-2H]2-	788.4709	788.4663	OxCL(18:1_18:1(OOH)_18:2(OOH)2_18:3(OOH))
$C_{83}H_{146}O_{24}P_2$	[M-2H]2-	793.4718	793.4767	OxCL(18:1_18:1(OH)_18:2(OOH)_20:4(OOH)2)
$C_{85}H_{144}O_{23}P_2$	[M-2H]2-	796.4753	796.4714	OxCL(18:1_18:3(OH)_20:3(OOH)2_20:4(OH))
$C_{85}H_{148}O_{23}P_2$	[M-2H]2-	798.4890	798.4870	OxCL(18:1_18:1(OOH)_20:3(OOH)2_20:4)
$C_{85}H_{142}O_{24}P_2$	[M-2H]2-	803.4626	803.4610	OxCL(18:1(OOH)_18:3(OH)_20:4(OOH)_20:4(OOH))
$C_{85}H_{144}O_{25}P_2$	[M-2H]2-	812.4708	812.4663	OxCL(18:1_18:3(OOH)_20:3(OOH)2_20:4(OOH))
$C_{87}H_{150}O_{24}P_2$	[M-2H]2-	819.4879	819.4923	OxCL(18:1(OH)_20:3(OOH)_20:3(OOH)_20:3(OOH))
$C_{89}H_{148}O_{23}P_2$	[M-2H]2-	822.4897	822.4870	OxCL(20:3(OOH)_20:3(OOH)_20:3(OOH)_20:4)
$C_{83}H_{136}O_{29}P_2$	[M-2H]2-	828.4255	828.4248	OxCL(18:3(OOH)_18:3(OOH)_18:3(OOH)2_20:4(OOH)2)
$C_{85}H_{144}O_{27}P_2$	[M-2H]2-	828.4581	828.4612	OxCL(18:1_18:2(OOH)_20:4(OOH)2_20:4(OOH)2)
$C_{85}H_{148}O_{27}P_2$	[M-2H]2-	830.4734	830.4769	OxCL(18:1_18:1(OOH)_20:3(OOH)2_20:4(OOH)2)
$C_{81}H_{142}O_{31}P_2$	[M-2H]2-	835.4424	835.4432	OxCL(18:2(OOH)_18:2(OOH)2_18:2(OOH)2_18:2(OOH)2)
$C_{85}H_{144}O_{29}P_2$	[M-2H]2-	844.4541	844.4561	OxCL(18:1_18:2(OOH)2_20:4(OOH)2_20:4(OOH)2)
$C_{85}H_{148}O_{29}P_2$	[M-2H]2-	846.4675	846.4718	OxCL(18:1_18:2(OOH)2_20:3(OOH)2_20:3(OOH)2)
$C_{85}H_{140}O_{31}P_2$	[M-2H]2-	858.4347	858.4354	OxCL(18:2(OOH)2_18:3(OOH)_20:4(OOH)2_20:4(OOH)2)
$C_{85}H_{148}O_{31}P_2$	[M-2H]2-	862.4621	862.4667	OxCL(18:1(OOH)_18:2(OOH)2_20:3(OOH)2_20:3(OOH)2)
$C_{37}H_{64}NO_{10}P$	[M+HCO2]-	758.4278	758.4250	OxPC(18:3(OH)_11:1(CHO))
$C_{42}H_{78}NO_9P$	[M+HCO2]-	816.5378	816.5396	OxPC(16:0_18:3(1O))
$C_{42}H_{80}NO_9P$	[M+HCO2]-	818.5529	818.5553	OxPC(16:0_18:2(1O))
$C_{42}H_{82}NO_9P$	[M+HCO2]-	820.5684	820.5709	OxPC(16:0_18:1(1O))
$C_{43}H_{78}NO_9P$	[M+HCO2]-	828.5394	828.5396	OxPC(17:0_18:4(OH))
$C_{42}H_{80}NO_{10}P$	[M+HCO2]-	834.5506	834.5502	OxPC(16:0_18:2(2O))
$C_{44}H_{76}NO_9P$	[M+HCO2]-	838.5243	838.5240	OxPC(18:1_18:4(CHO))
$C_{44}H_{78}NO_9P$	[M+HCO2]-	840.5367	840.5396	OxPC(18:2_18:2(Ke))
$C_{44}H_{80}NO_9P$	[M+HCO2]-	842.5537	842.5553	OxPC(18:1_18:3(1O))
$C_{44}H_{82}NO_9P$	[M+HCO2]-	844.5684	844.5709	OxPC(18:1_18:2(1O))
$C_{42}H_{76}NO_{11}P$	[M+HCO2]-	846.5115	846.5138	OxPC(16:0_18:4(OOO))
$C_{44}H_{84}NO_9P$	[M+HCO2]-	846.5846	846.5866	OxPC(18:0_18:2(1O))
$C_{42}H_{80}NO_{11}P$	[M+HCO2]-	850.5445	850.5451	OxPC(16:1_18:1(OOO))

$C_{44}H_{74}NO_{10}P$	[M+HCO2]-	852.5038	852.5032	OxPC(18:2_18:4(COOH))
$C_{42}H_{82}NO_{11}P$	[M+HCO2]-	852.5607	852.5607	OxPC(16:0_18:1(3O))
$C_{44}H_{78}NO_{10}P$	[M+HCO2]-	856.5345	856.5345	OxPC(16:0_20:5(2O))
$C_{44}H_{80}NO_{10}P$	[M+HCO2]-	858.5524	858.5502	OxPC(16:0_20:3(2O(1Cyc)))
$C_{44}H_{82}NO_{10}P$	[M+HCO2]-	860.5652	860.5658	OxPC(18:1_18:2(2O))
$C_{44}H_{84}NO_{10}P$	[M+HCO2]-	862.5793	862.5815	OxPC(18:0_18:2(2O))
$C_{44}H_{82}NO_{11}P$	[M+HCO2]-	876.5609	876.5607	OxPC(18:0_18:3(OOO))
$C_{44}H_{84}NO_{11}P$	[M+HCO2]-	878.5768	878.5764	OxPC(18:1_18:1(3O))
$C_{44}H_{86}NO_{11}P$	[M+HCO2]-	880.5923	880.5920	OxPC(18:0_18:1(3O))
$C_{32}H_{60}NO_{10}P$	[M-H]-	648.3861	648.3882	OxPE(16:0_11:1(COOH))
$C_{34}H_{66}NO_{9}P$	[M-H]-	662.4383	662.4402	OxPE(18:0_11:0(CHO))
$C_{35}H_{66}NO_{10}P$	[M-H]-	690.4330	690.4351	OxPE(18:0_12:1(COOH))
$C_{35}H_{68}NO_{10}P$	[M-H]-	692.4491	692.4508	OxPE(19:0_11:0(COOH))
$C_{39}H_{78}NO_{9}P$	[M-H]-	734.5361	734.5341	OxPE(16:0_18:0(1O))
$C_{39}H_{74}NO_{10}P$	[M-H]-	746.4961	746.4977	OxPE(16:0_18:2(2O))
$C_{41}H_{72}NO_{9}P$	[M-H]-	752.4858	752.4872	OxPE(18:2_18:3(OH))
$C_{41}H_{80}NO_{9}P$	[M-H]-	760.5521	760.5498	OxPE(18:1_18:0(1O))
$C_{41}H_{82}NO_{9}P$	[M-H]-	762.5632	762.5654	OxPE(18:0_18:0(1O))
$C_{41}H_{76}NO_{10}P$	[M-H]-	772.5120	772.5134	OxPE(18:1_18:2(2O))
$C_{41}H_{78}NO_{10}P$	[M-H]-	774.5281	774.5290	OxPE(18:0_18:2(2O))
$C_{41}H_{80}NO_{10}P$	[M-H]-	776.5440	776.5447	OxPE(18:1_18:0(2O))
$C_{43}H_{74}NO_{9}P$	[M-H]-	778.5019	778.5028	OxPE(18:0_20:5(1O(1Cyc)))
$C_{43}H_{78}NO_{9}P$	[M-H]-	782.5316	782.5341	OxPE(18:1_20:3(1O))
$C_{43}H_{80}NO_{9}P$	[M-H]-	784.5479	784.5498	OxPE(18:0_20:3(1O))
$C_{41}H_{76}NO_{11}P$	[M-H]-	788.5065	788.5083	OxPE(16:0e/20:4(4O(1Cyc)))
$C_{41}H_{78}NO_{11}P$	[M-H]-	790.5220	790.5239	OxPE(18:1_18:1(3O))
$C_{43}H_{72}NO_{10}P$	[M-H]-	792.4814	792.4821	OxPE(18:1_20:5(KeOH))
$C_{41}H_{80}NO_{11}P$	[M-H]-	792.5388	792.5396	OxPE(18:0_18:1(3O))
$C_{43}H_{74}NO_{10}P$	[M-H]-	794.4968	794.4977	OxPE(18:1_20:5(2O))
$C_{43}H_{76}NO_{10}P$	[M-H]-	796.5117	796.5134	OxPE(18:1_20:4(OOH))
$C_{43}H_{78}NO_{10}P$	[M-H]-	798.5270	798.5290	OxPE(18:1_20:3(2O))
$C_{43}H_{80}NO_{10}P$	[M-H]-	800.5419	800.5447	OxPE(18:0_20:3(2O))
$C_{41}H_{74}NO_{12}P$	[M-H]-	802.4855	802.4876	OxPE(16:0_20:4(4O(1Cyc)))
$C_{41}H_{76}NO_{12}P$	[M-H]-	804.5013	804.5032	OxPE(18:1_18:2(OOOO))
$C_{45}H_{78}NO_{9}P$	[M-H]-	806.5371	806.5341	OxPE(20:1_20:5(OH))
$C_{41}H_{80}NO_{12}P$	[M-H]-	808.5319	808.5345	OxPE(18:0_18:1(OOOO))
$C_{43}H_{74}NO_{11}P$	[M-H]-	810.4914	810.4926	OxPE(18:1_20:5(3O))
$C_{43}H_{76}NO_{11}P$	[M-H]-	812.5071	812.5083	OxPE(18:1_20:4(3O))
$C_{43}H_{74}NO_{12}P$	[M-H]-	826.4853	826.4876	OxPE(18:1_20:5(4O(2Cyc)))
$C_{43}H_{76}NO_{12}P$	[M-H]-	828.5014	828.5032	OxPE(18:0_20:5(4O(2Cyc)))
$C_{43}H_{78}NO_{12}P$	[M-H]-	830.5169	830.5189	OxPE(18:1_20:3(4O(1Cyc)))
$C_{43}H_{80}NO_{12}P$	[M-H]-	832.5321	832.5345	OxPE(18:0_20:3(4O(1Cyc)))
$C_{43}H_{76}NO_{13}P$	[M-H]-	844.4955	844.4981	OxPE(18:1(OH)_20:4(OOOO))
$C_{45}H_{83}O_{14}P$	[M-H]-	877.5430	877.5447	OxPI(18:1_18:1(1O))
$C_{40}H_{74}NO_{11}P$	[M-H]-	774.4908	774.4926	OxPS(16:0_18:2(1O))
$C_{42}H_{76}NO_{11}P$	[M-H]-	800.5056	800.5083	OxPS(18:1_18:1(1O(1Cyc)))
$C_{42}H_{78}NO_{11}P$	[M-H]-	802.5220	802.5239	OxPS(18:1_18:0(1O(1Cyc)))
$C_{42}H_{76}NO_{12}P$	[M-H]-	816.5013	816.5032	OxPS(18:1_18:2(2O))
$C_{42}H_{78}NO_{12}P$	[M-H]-	818.5170	818.5189	OxPS(18:0_18:2(2O))
$C_{44}H_{74}NO_{11}P$	[M-H]-	822.4888	822.4926	OxPS(18:1_20:4(1O(1Cyc)))

$C_{44}H_{76}NO_{11}P$	[M-H]-	824.5080	824.5083	OxPS(18:0_20:5(1O))
$C_{42}H_{78}NO_{13}P$	[M-H]-	834.5115	834.5138	OxPS(18:1_18:1(3O))
$C_{42}H_{80}NO_{13}P$	[M-H]-	836.5273	836.5294	OxPS(18:0_18:1(3O))
$C_{44}H_{74}NO_{12}P$	[M-H]-	838.4844	838.4876	OxPS(18:1_20:5(2O))
$C_{44}H_{76}NO_{12}P$	[M-H]-	840.5000	840.5032	OxPS(18:1_20:3(2O(1Cyc)))
$C_{44}H_{76}NO_{13}P$	[M-H]-	856.4946	856.4981	OxPS(18:0_20:5(3O))
$C_{44}H_{78}NO_{13}P$	[M-H]-	858.5121	858.5138	OxPS(18:0_20:4(3O))
$C_{39}H_{65}O_{8}P$	[M-H]-	691.4351	691.4344	PA(18:3_18:3)
$C_{39}H_{73}O_{8}P$	[M-H]-	699.4958	699.4970	PA(18:0_18:2)
$C_{36}H_{72}NO_{8}P$	[M+HCO2]-	722.4998	722.4977	PC(14:0_14:0)
$C_{38}H_{76}NO_{8}P$	[M+HCO2]-	750.5327	750.5290	PC(15:0_15:0)
$C_{40}H_{80}NO_{8}P$	[M+HCO2]-	778.5650	778.5603	PC(14:0_18:0)
$C_{42}H_{82}NO_{8}P$	[M+HCO2]-	804.5737	804.5760	PC(16:0_18:1)
$C_{42}H_{84}NO_{8}P$	[M+HCO2]-	806.5897	806.5916	PC(16:0_18:0)
$C_{43}H_{78}NO_{8}P$	[M+HCO2]-	812.5400	812.5447	PC(17:1_18:3)
$C_{43}H_{84}NO_{8}P$	[M+HCO2]-	818.5900	818.5916	PC(16:1_19:0)
$C_{43}H_{86}NO_{8}P$	[M+HCO2]-	820.6033	820.6073	PC(17:0_18:0)
$C_{44}H_{76}NO_{8}P$	[M+HCO2]-	822.5270	822.5290	PC(18:3_18:3)
$C_{44}H_{78}NO_{8}P$	[M+HCO2]-	824.5410	824.5447	PC(18:1_18:4)
$C_{44}H_{80}NO_{8}P$	[M+HCO2]-	826.5583	826.5603	PC(18:0_18:4)
$C_{44}H_{82}NO_{8}P$	[M+HCO2]-	828.5764	828.5760	PC(18:1_18:2)
$C_{44}H_{84}NO_{8}P$	[M+HCO2]-	830.5896	830.5916	PC(18:1_18:1)
$C_{44}H_{86}NO_{8}P$	[M+HCO2]-	832.6028	832.6073	PC(18:0_18:1)
$C_{45}H_{86}NO_{8}P$	[M+HCO2]-	844.6058	844.6073	PC(18:2_19:0)
$C_{46}H_{80}NO_{8}P$	[M+HCO2]-	850.5576	850.5603	PC(18:1_20:5)
$C_{46}H_{82}NO_{8}P$	[M+HCO2]-	852.5766	852.5760	PC(18:2_20:3)
$C_{46}H_{86}NO_{8}P$	[M+HCO2]-	856.6059	856.6073	PC(18:1_20:2)
$C_{35}H_{68}NO_{8}P$	[M-H]-	660.4609	660.4610	PE(14:0_16:1)
$C_{39}H_{76}NO_{8}P$	[M-H]-	716.5261	716.5236	PE(16:1_18:0)
$C_{41}H_{78}NO_{8}P$	[M-H]-	742.5372	742.5392	PE(18:0_18:2)
$C_{43}H_{80}NO_{8}P$	[M-H]-	768.5526	768.5549	PE(18:3_20:0)
$C_{43}H_{82}NO_{8}P$	[M-H]-	770.5692	770.5705	PE(18:1_20:1)
$C_{38}H_{69}O_{10}P$	[M-H]-	715.4537	715.4555	PG(14:0_18:3)
$C_{38}H_{71}O_{10}P$	[M-H]-	717.4702	717.4712	PG(16:1_16:1)
$C_{38}H_{73}O_{10}P$	[M-H]-	719.4848	719.4868	PG(16:0_16:1)
$C_{40}H_{71}O_{10}P$	[M-H]-	741.4704	741.4712	PG(16:0_18:4)
$C_{40}H_{73}O_{10}P$	[M-H]-	743.4869	743.4868	PG(16:1_18:2)
$C_{40}H_{75}O_{10}P$	[M-H]-	745.5020	745.5025	PG(17:1_17:1)
$C_{41}H_{81}O_{10}P$	[M-H]-	763.5501	763.5494	PG(15:0_20:0)
$C_{42}H_{79}O_{10}P$	[M-H]-	773.5352	773.5338	PG(16:0_20:2)
$C_{41}H_{79}O_{13}P$	[M-H]-	809.5165	809.5185	PI(16:0_16:0)
$C_{42}H_{79}O_{13}P$	[M-H]-	821.5176	821.5185	PI(16:1_17:0)
$C_{43}H_{79}O_{13}P$	[M-H]-	833.5165	833.5185	PI(16:0_18:2)
$C_{43}H_{81}O_{13}P$	[M-H]-	835.5324	835.5342	PI(14:0_20:1)
$C_{44}H_{81}O_{13}P$	[M-H]-	847.5335	847.5342	PI(17:2_18:0)
$C_{44}H_{83}O_{13}P$	[M-H]-	849.5477	849.5498	PI(17:1_18:0)
$C_{45}H_{79}O_{13}P$	[M-H]-	857.5162	857.5185	PI(18:2_18:2)
$C_{45}H_{81}O_{13}P$	[M-H]-	859.5323	859.5342	PI(16:1_20:2)
$C_{45}H_{83}O_{13}P$	[M-H]-	861.5477	861.5498	PI(18:0_18:2)
$C_{45}H_{85}O_{13}P$	[M-H]-	863.5634	863.5655	PI(16:0_20:1)

$C_{47}H_{83}O_{13}P$	[M-H]-	885.5476	885.5498	PI(16:1_22:3)
$C_{40}H_{74}NO_{10}P$	[M-H]-	758.4956	758.4977	PS(16:0_18:2)
$C_{41}H_{78}NO_{10}P$	[M-H]-	774.5273	774.5290	PS(17:1_18:0)
$C_{42}H_{72}NO_{10}P$	[M-H]-	780.4820	780.4821	PS(36:5)
$C_{42}H_{74}NO_{10}P$	[M-H]-	782.4940	782.4977	PS(18:2_18:2)
$C_{42}H_{76}NO_{10}P$	[M-H]-	784.5094	784.5134	PS(36:3)
$C_{42}H_{78}NO_{10}P$	[M-H]-	786.5285	786.5290	PS(18:1_18:1)
$C_{42}H_{80}NO_{10}P$	[M-H]-	788.5423	788.5447	PS(36:1)
$C_{42}H_{82}NO_{10}P$	[M-H]-	790.5585	790.5603	PS(18:0_18:0)
$C_{44}H_{74}NO_{10}P$	[M-H]-	806.4934	806.4977	PS(18:2_20:4)
$C_{44}H_{76}NO_{10}P$	[M-H]-	808.5109	808.5134	PS(38:5)
$C_{44}H_{78}NO_{10}P$	[M-H]-	810.5247	810.5290	PS(38:4)
$C_{44}H_{84}NO_{10}P$	[M-H]-	816.5729	816.5760	PS(38:1)
$C_{46}H_{74}NO_{10}P$	[M-H]-	830.4943	830.4977	PS(20:4_20:4)
$C_{46}H_{76}NO_{10}P$	[M-H]-	832.5095	832.5134	PS(40:7)
$C_{37}H_{64}O_{12}S$	[M-H]-	731.4037	731.4046	SQDG(28:3)

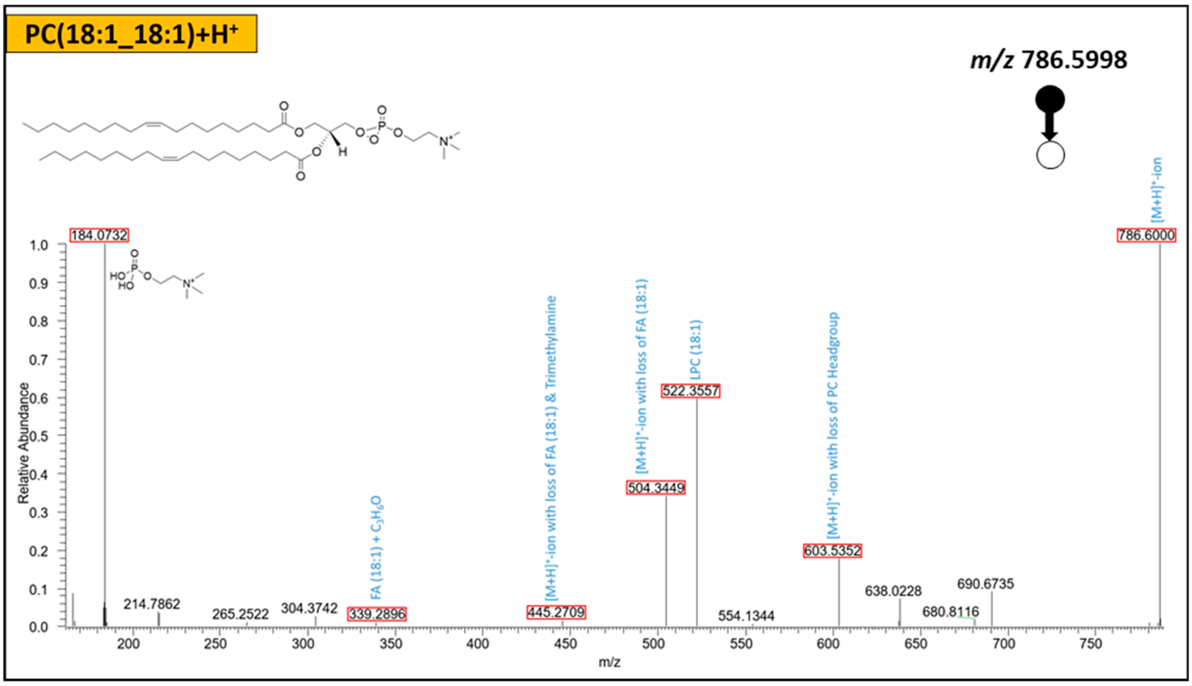

Figure S1. HCD MS/MS fragmentation pattern of PC(18:1_18:1)+H+.

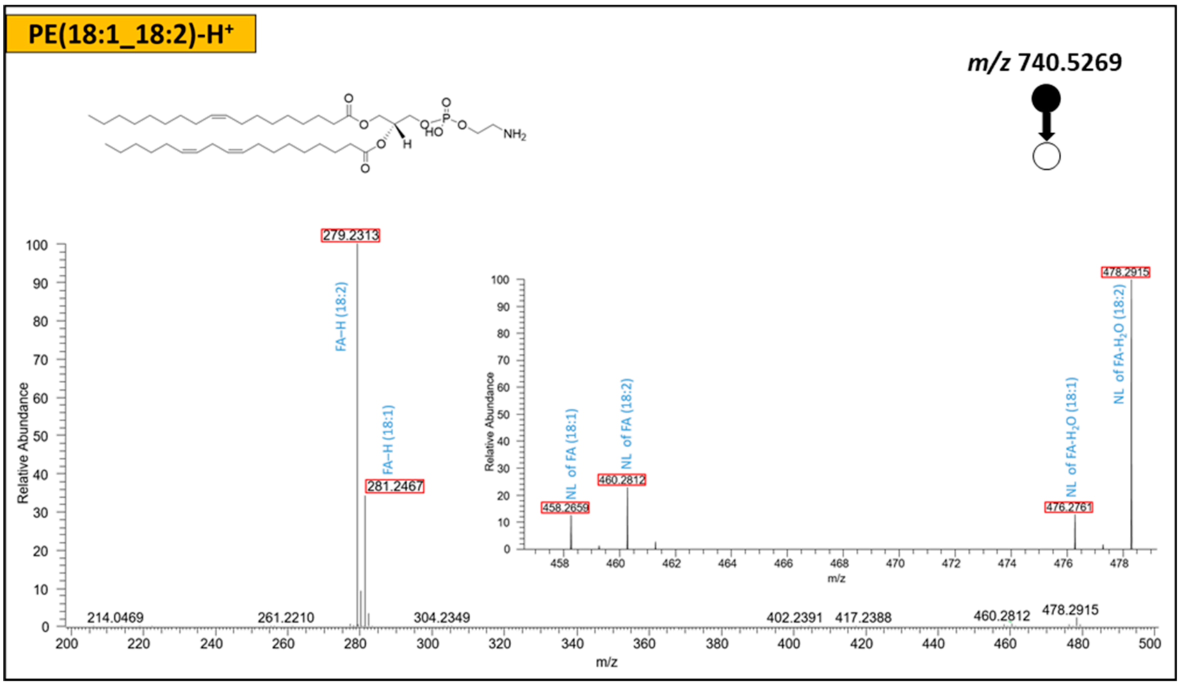

Figure S2. HCD MS/MS fragmentation pattern of PE(18:1_18:2)-H+.

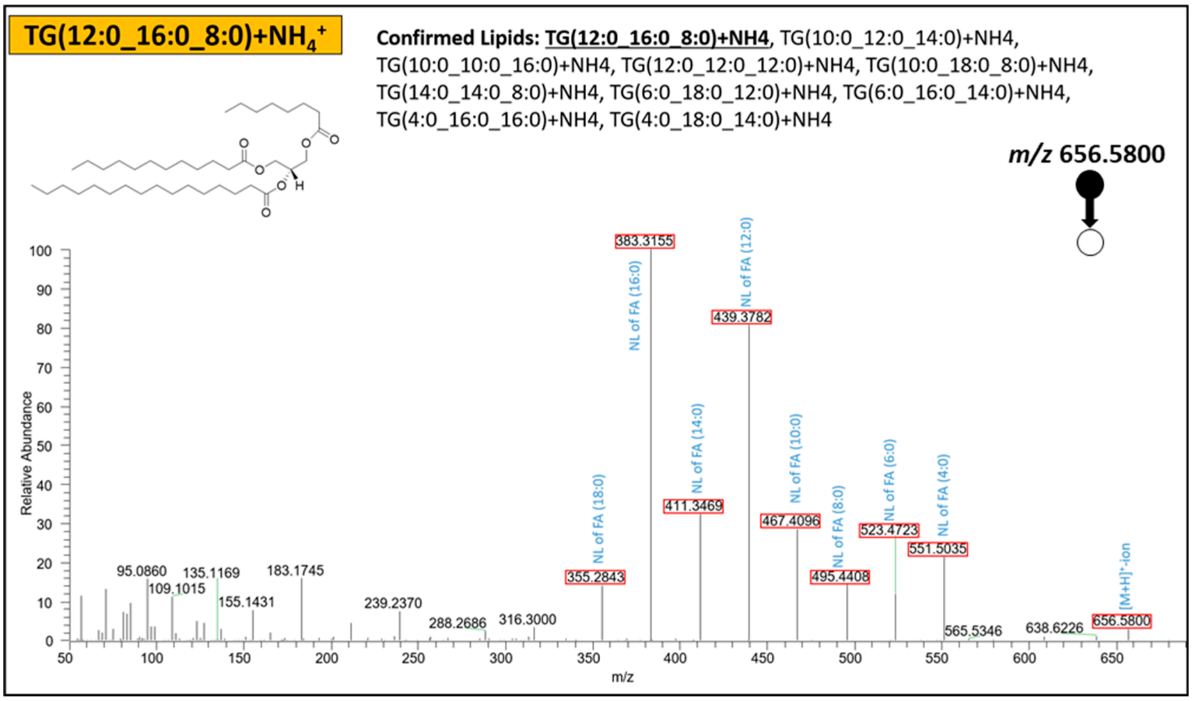

Figure S3. HCD MS/MS fragmentation pattern of TG(12:0_16:0_8:0)+NH4+.

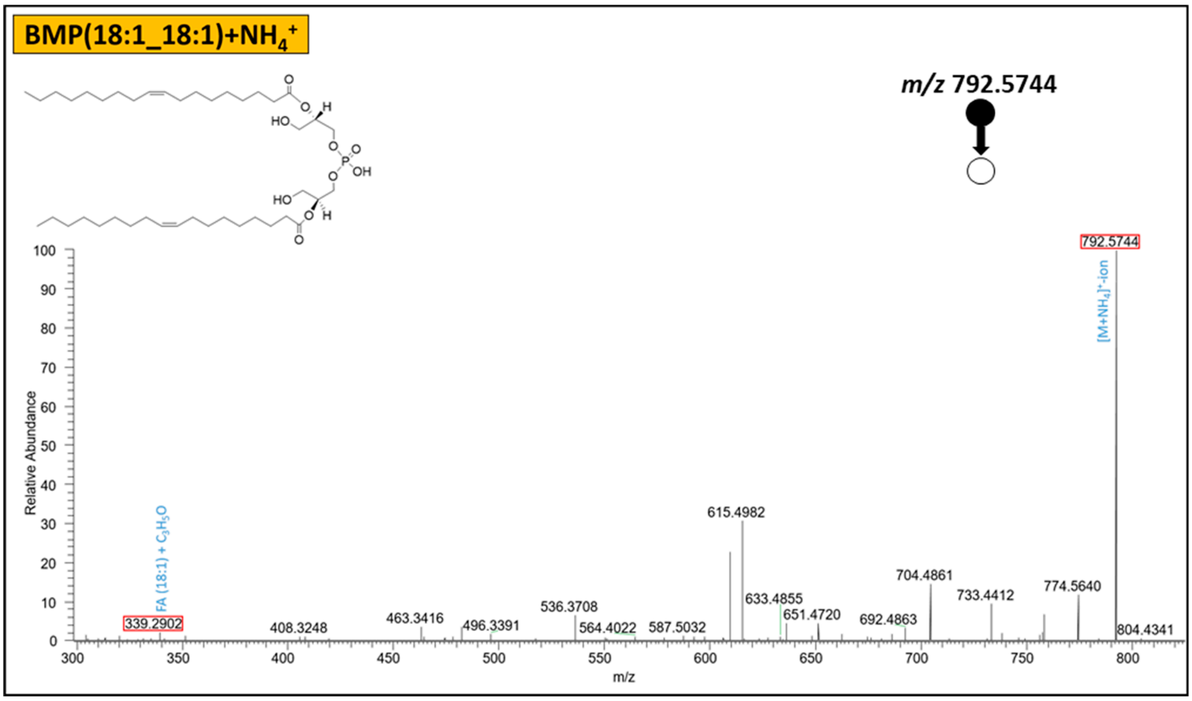

Figure S4. HCD MS/MS fragmentation pattern of BMP(18:1_18:1)+NH_4^+.

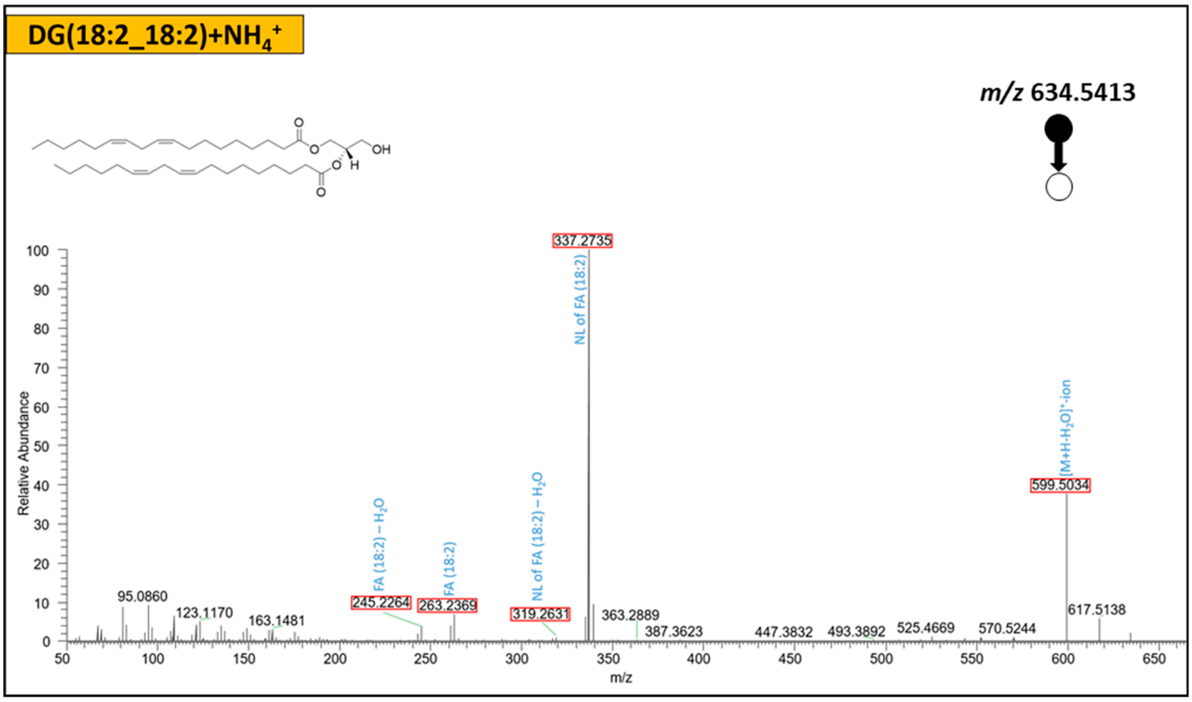

Figure S5. HCD MS/MS fragmentation pattern of DG(18:2_18:2)+NH_4^+.

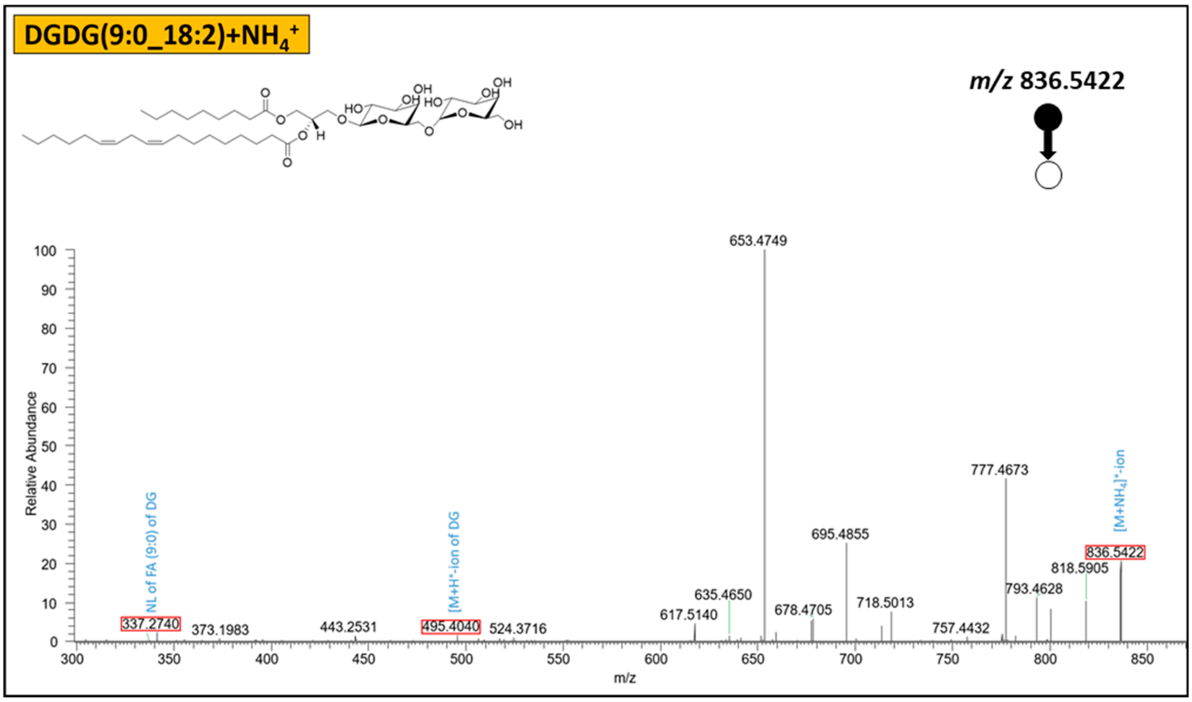

Figure S6. HCD MS/MS fragmentation pattern of DGDG(9:0_18:2)+NH_4^+.

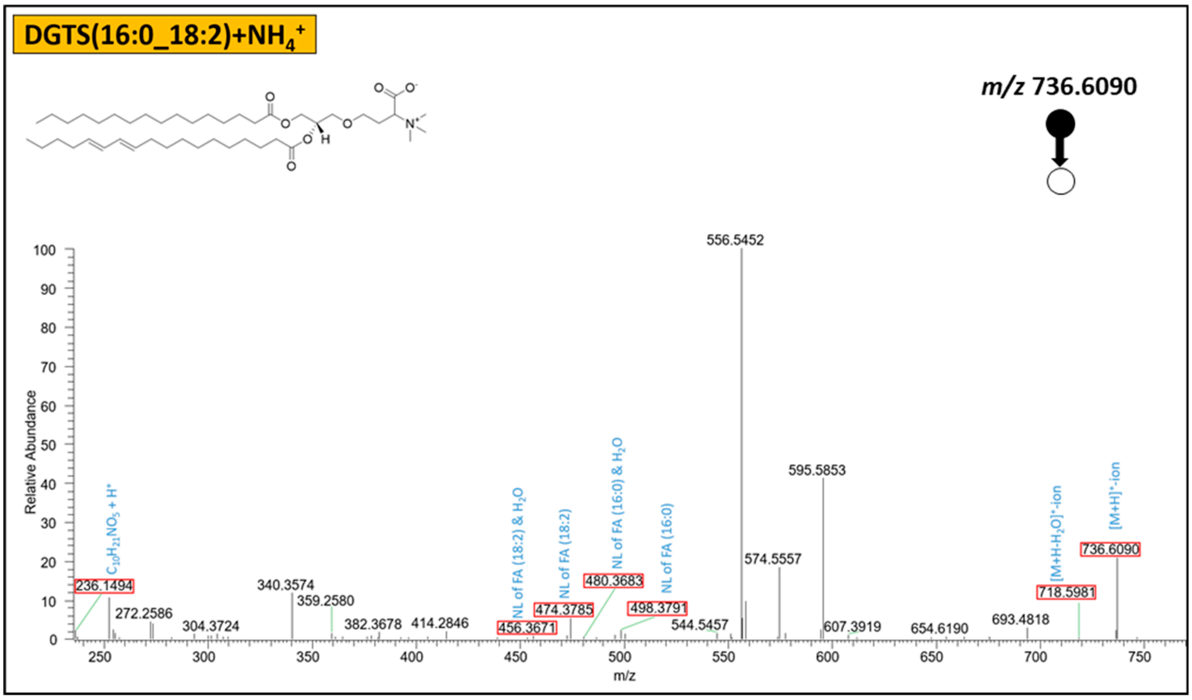

Figure S7. HCD MS/MS fragmentation pattern of DGTS(16:0_18:2)+NH_4^+.

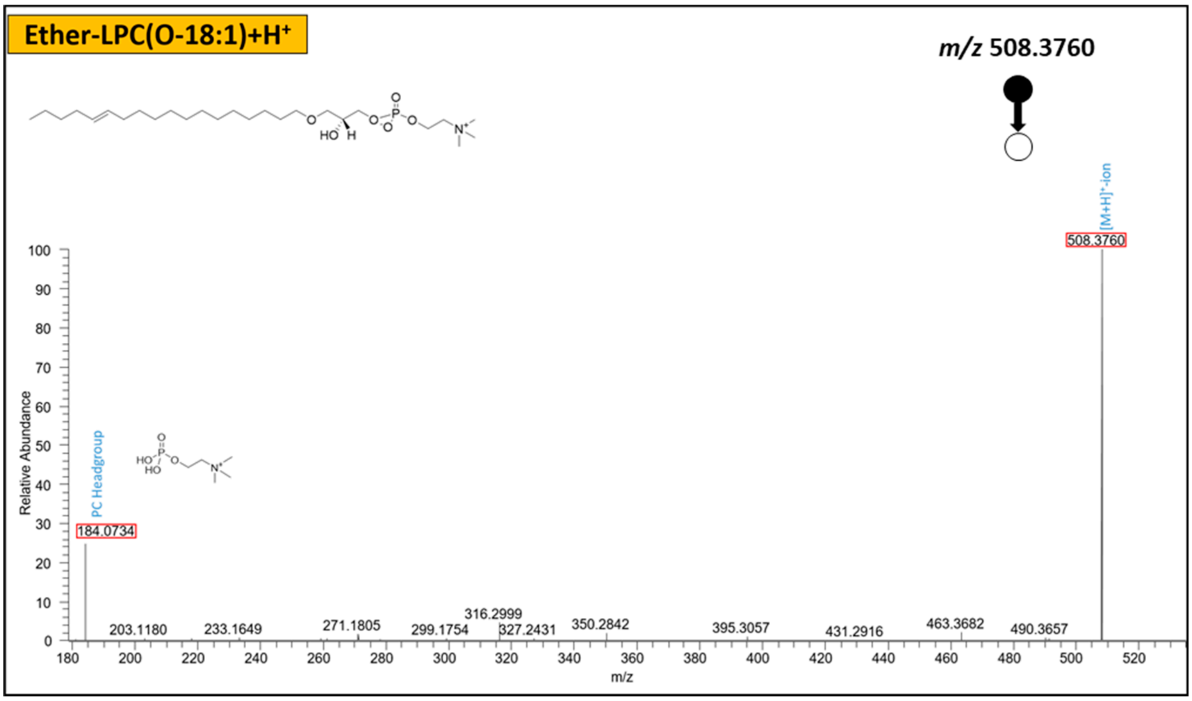

Figure S8. HCD MS/MS fragmentation pattern of ether-LPC(O-18:1)+H+.

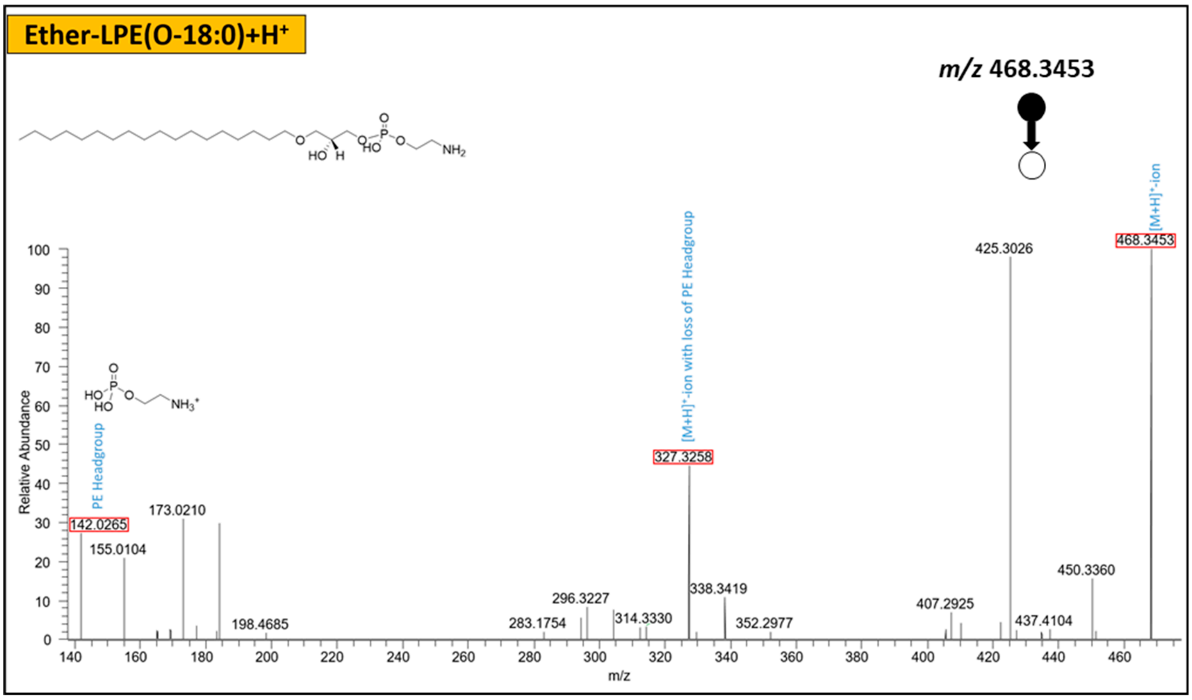

Figure S9. HCD MS/MS fragmentation pattern of ether-LPE(O-18:0)+H+.

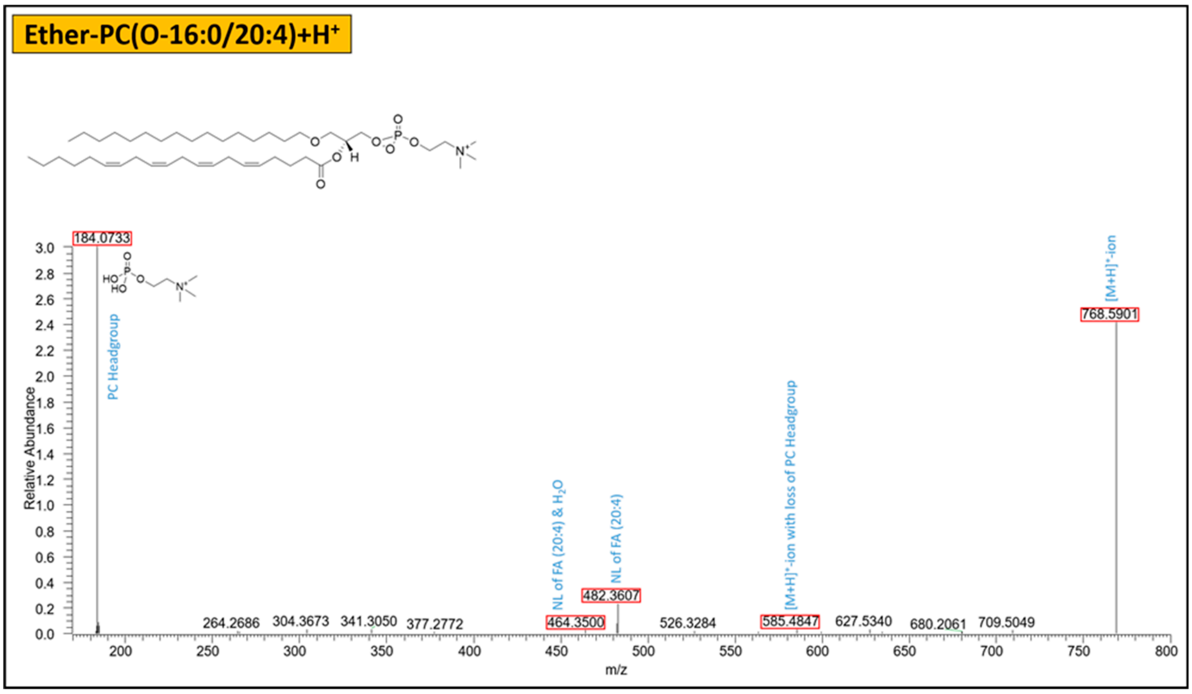

Figure S10. HCD MS/MS fragmentation pattern of ether-PC(O-16:0/20:4)+H+.

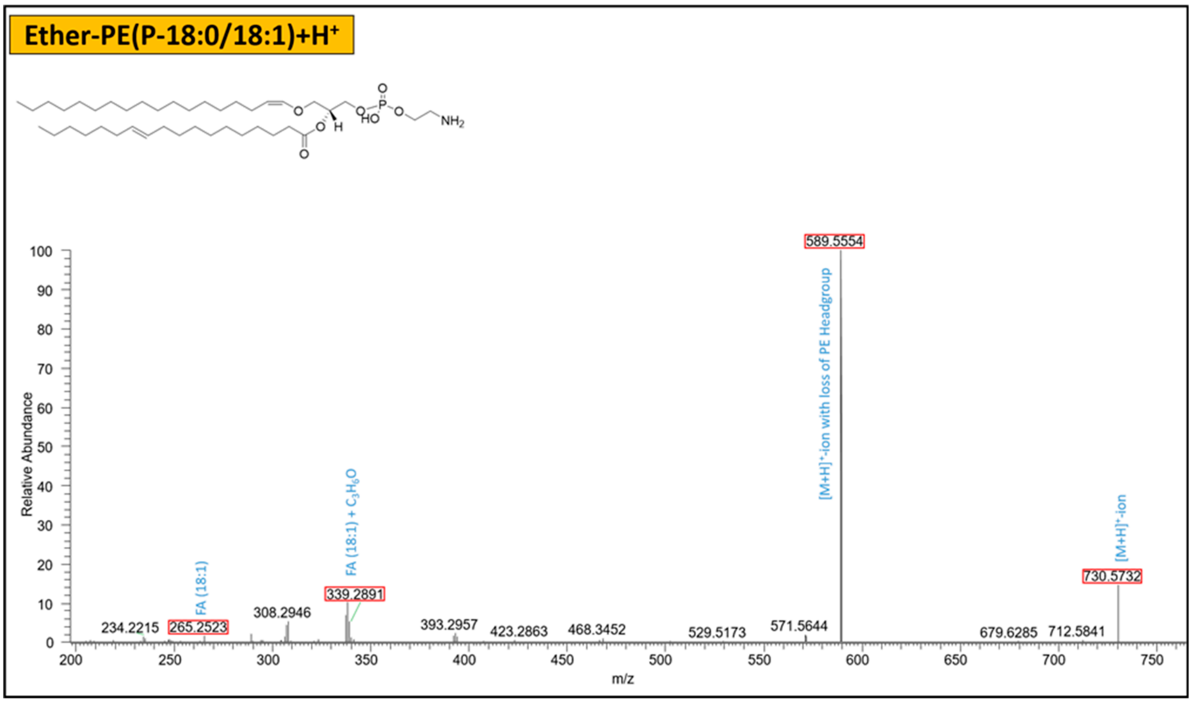

Figure S11. HCD MS/MS fragmentation pattern of ether-PE(P-18:0/18:1)+H+.

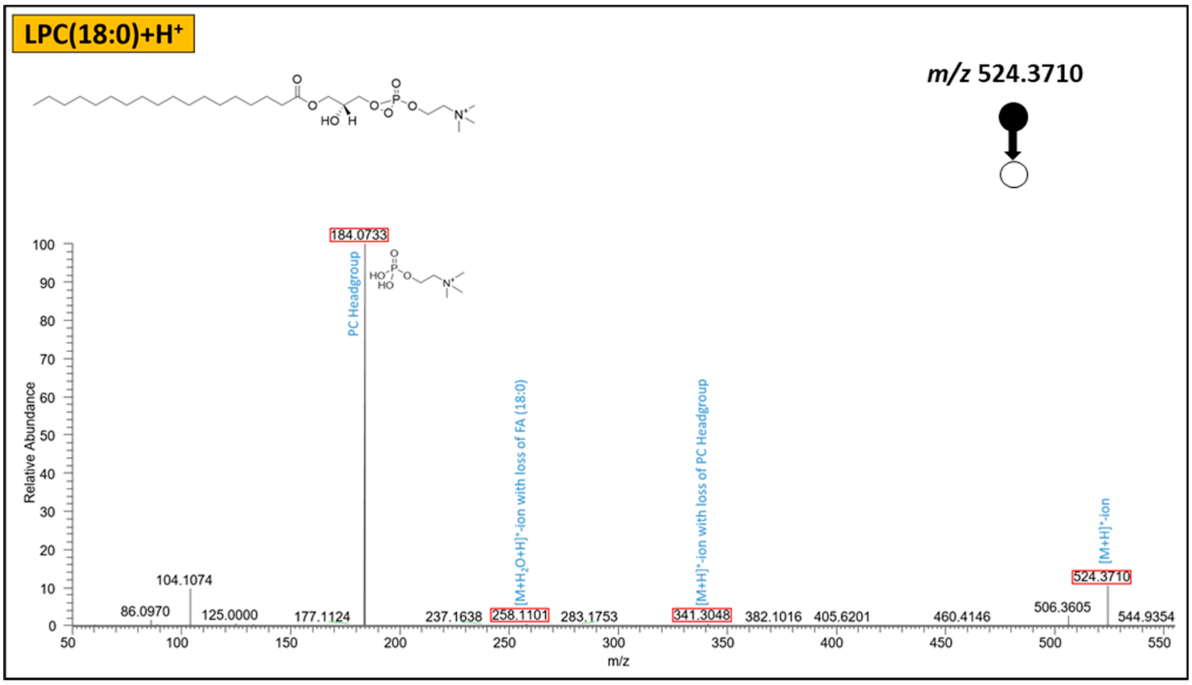

Figure S12. HCD MS/MS fragmentation pattern of LPC(18:0)+H+.

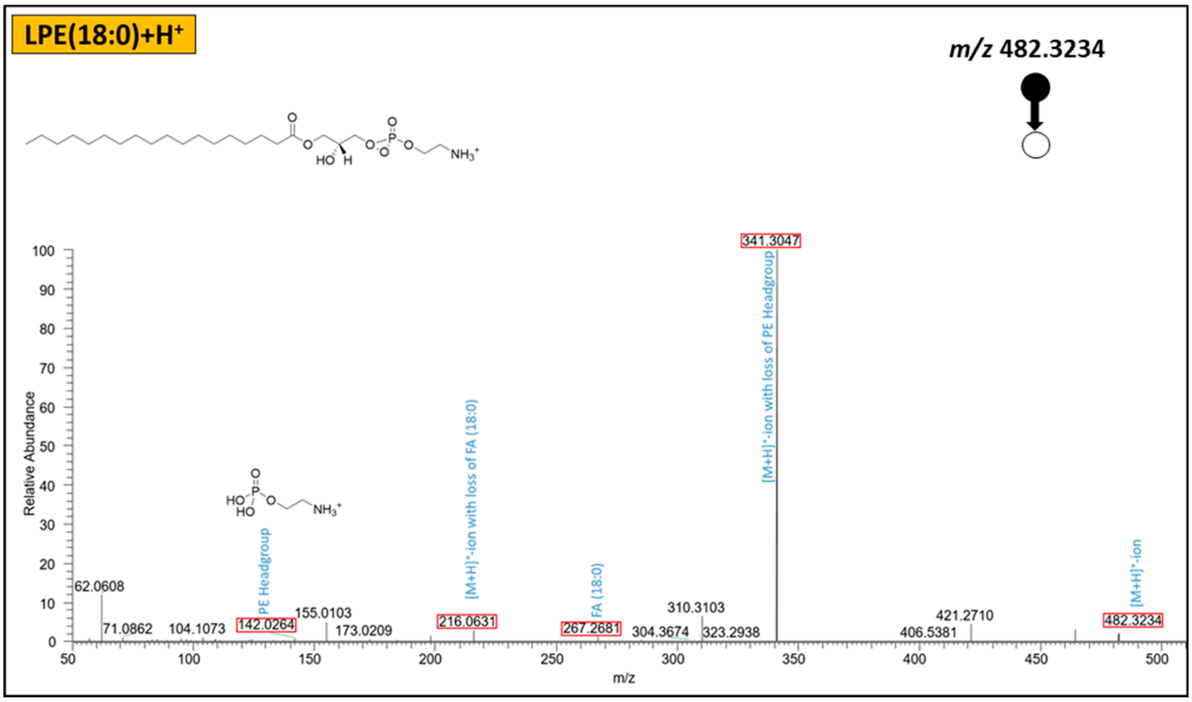

Figure S13. HCD MS/MS fragmentation pattern of LPE(18:0)+H+.

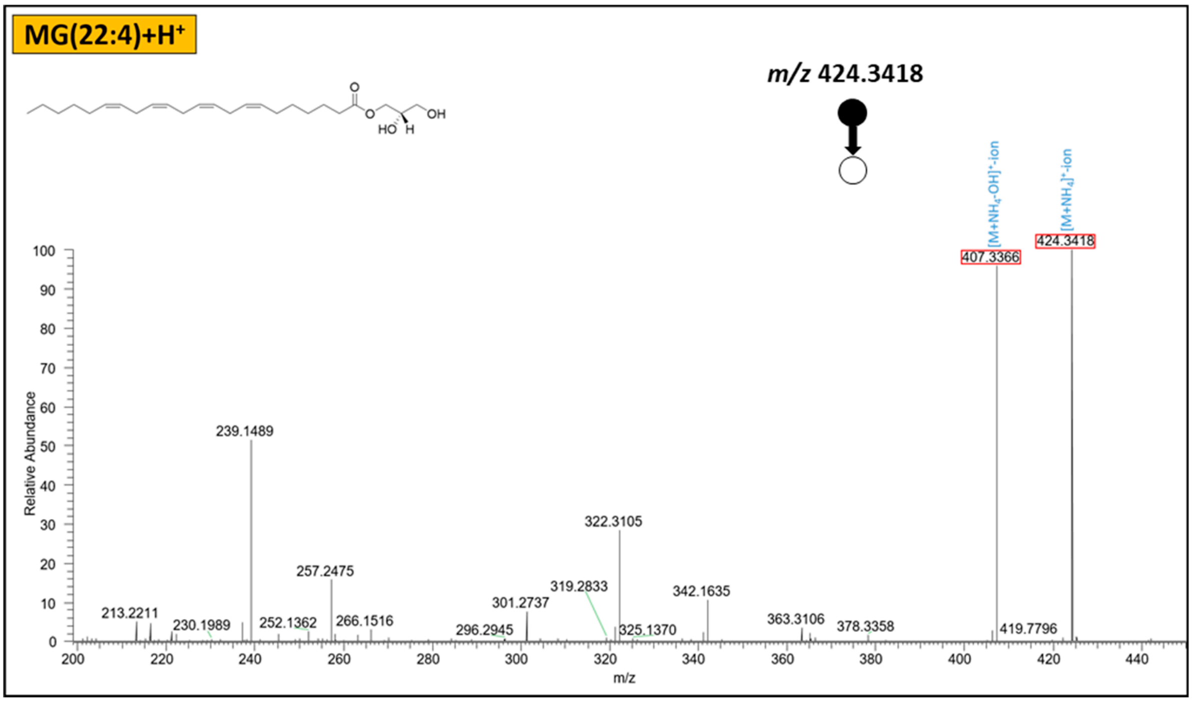

Figure S14. HCD MS/MS fragmentation pattern of MG(22:4)+H+.

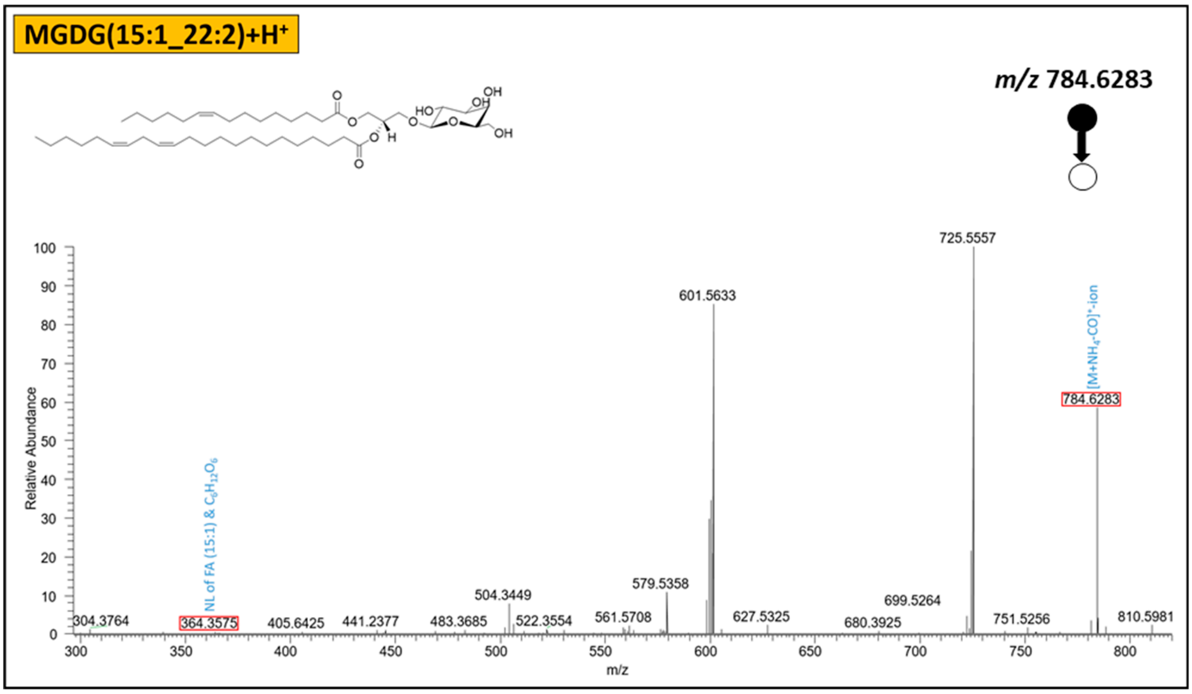

Figure S15. HCD MS/MS fragmentation pattern of MGDG(15:1_22:2)+H+.

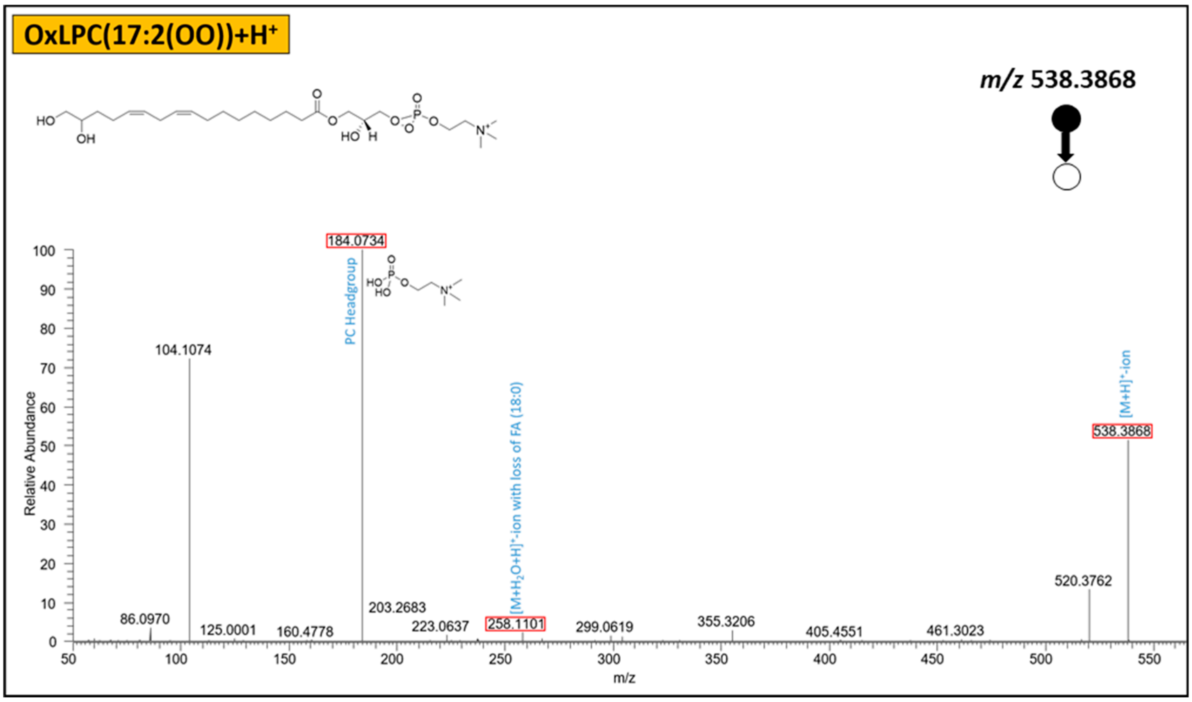

Figure S16. HCD MS/MS fragmentation pattern of OxLPC(17:2(OO))+H+.

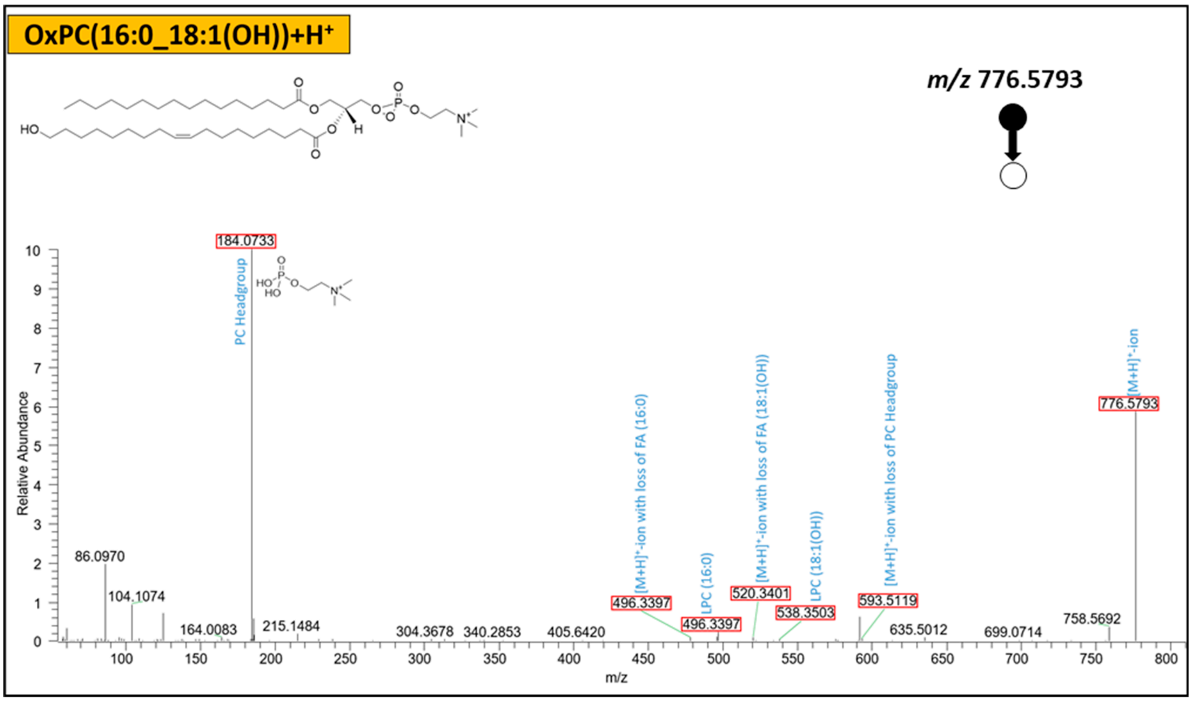

Figure S17. HCD MS/MS fragmentation pattern of OxPC(16:0_18:1(OH))+H+.

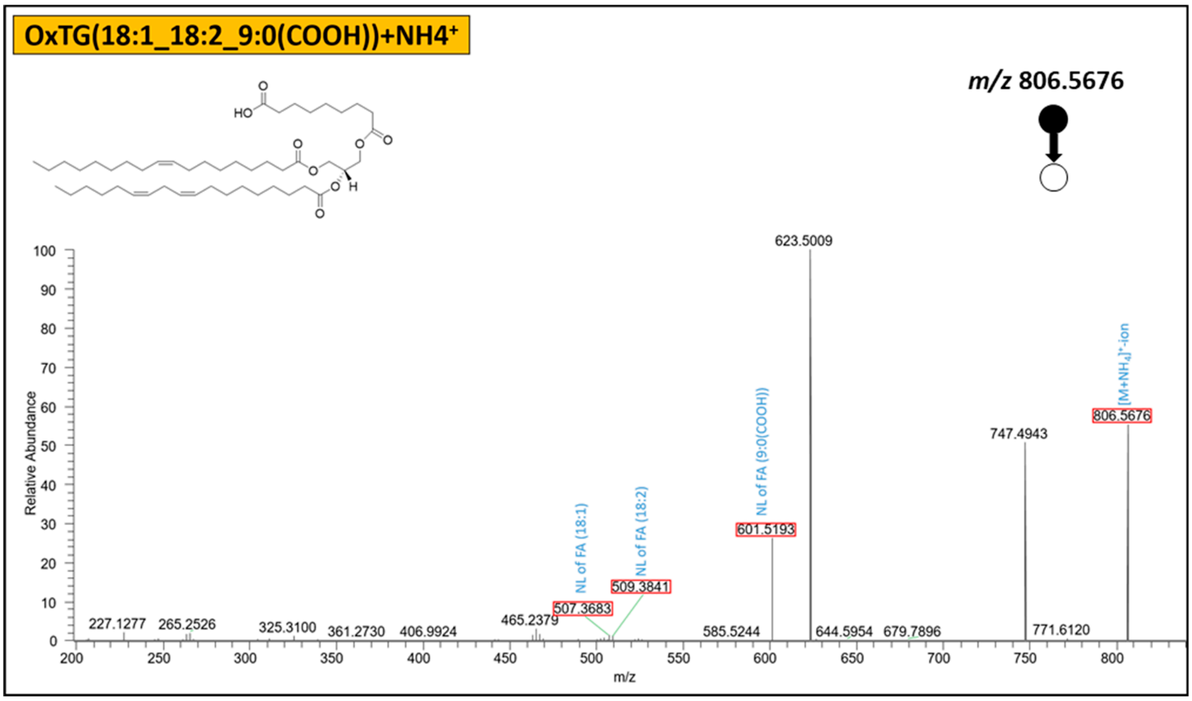

Figure S18. HCD MS/MS fragmentation pattern of OxTG(18:1_18:2_9:0(COOH))+NH_4^{+}.

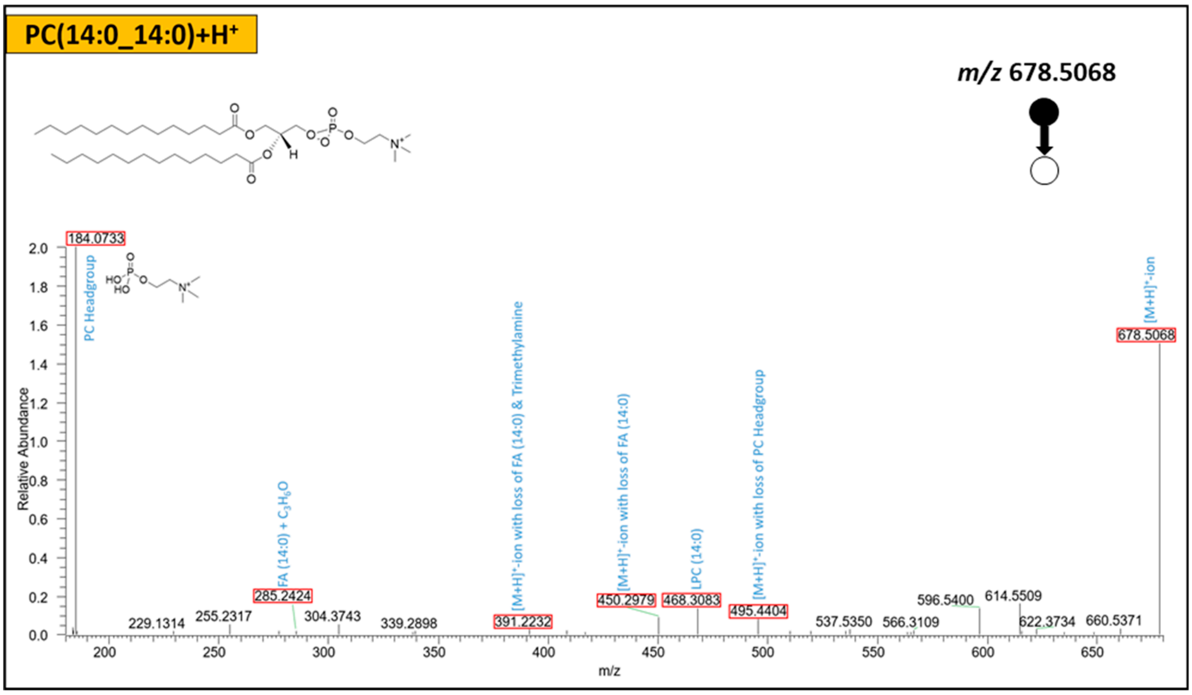

Figure S19. HCD MS/MS fragmentation pattern of PC(14:0_14:0)+H^{+}.

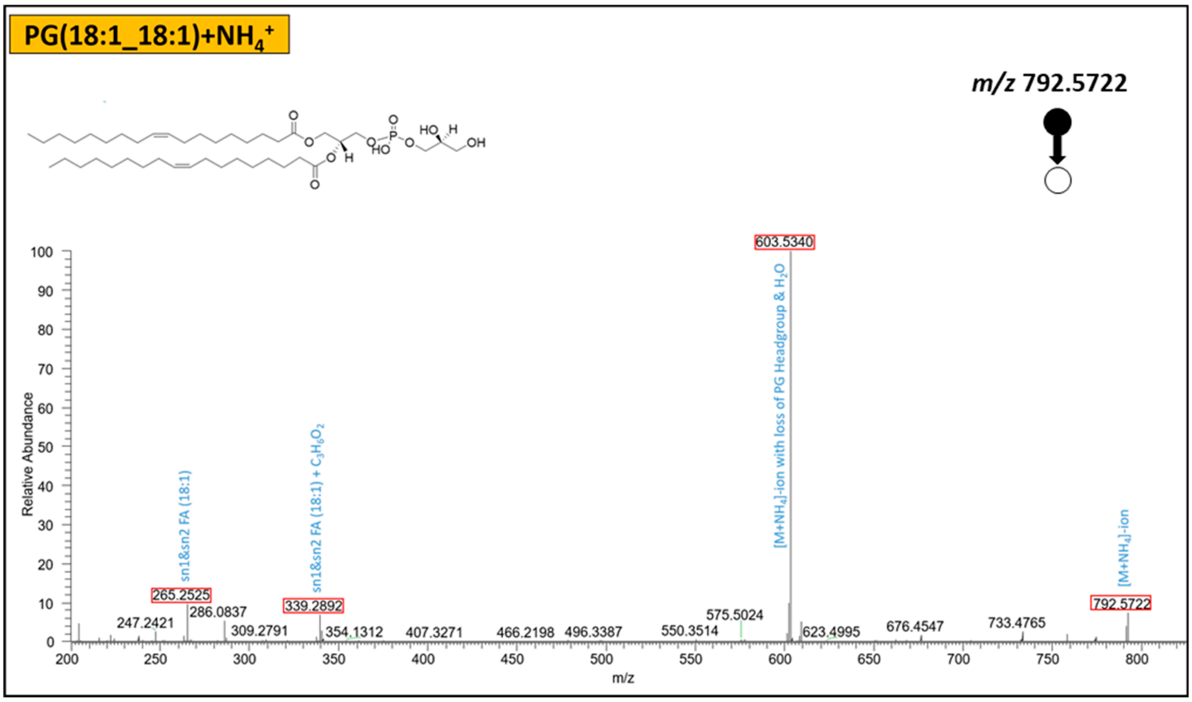

Figure S20. HCD MS/MS fragmentation pattern of PG(18:1_18:1)+NH_4^{+}.

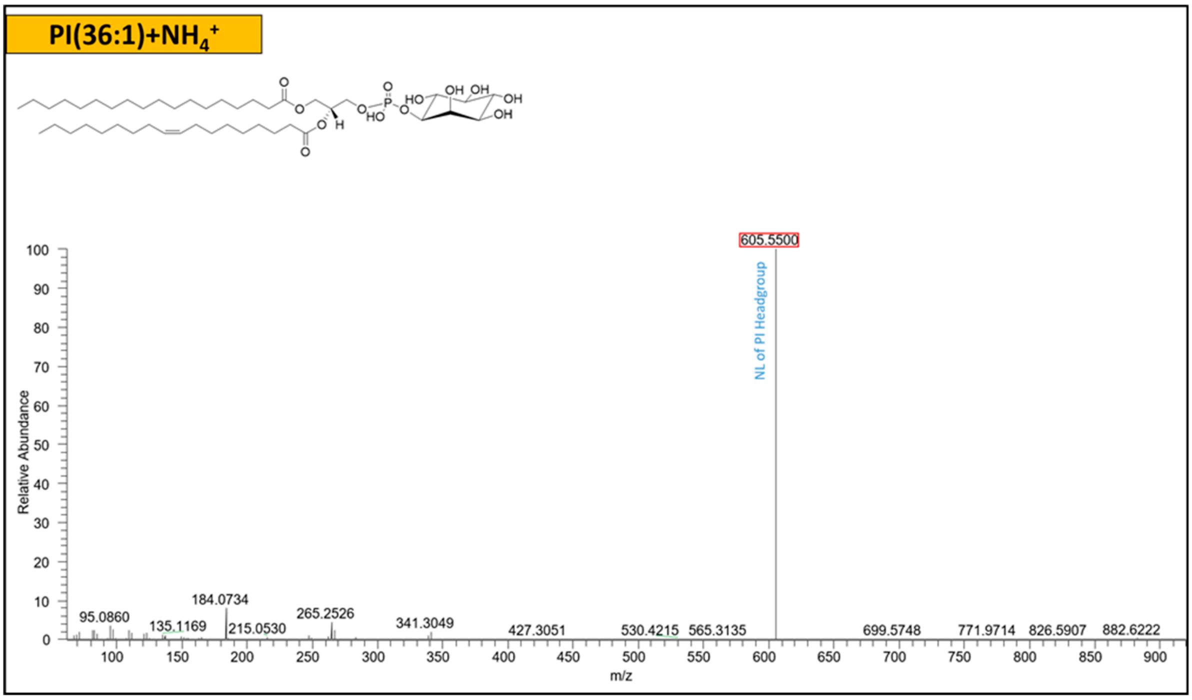

Figure S21. HCD MS/MS fragmentation pattern of PI(36:1)+ NH_4^{+}.

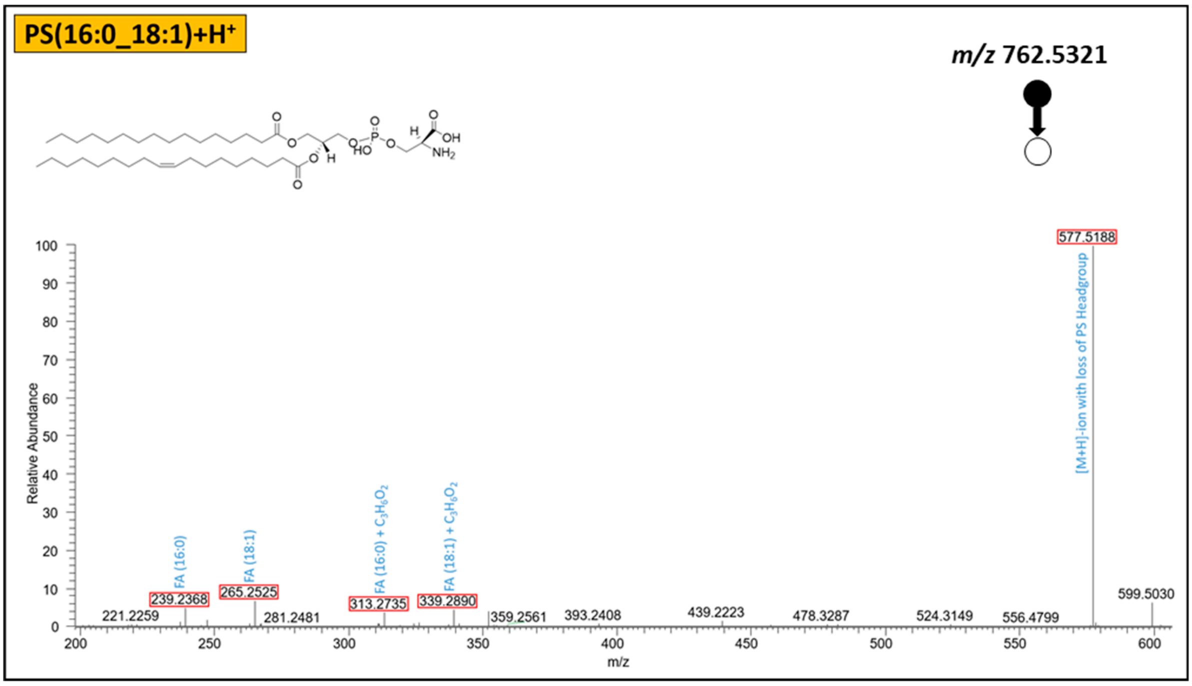

Figure S22. HCD MS/MS fragmentation pattern of PS(16:0_18:1)+H+.

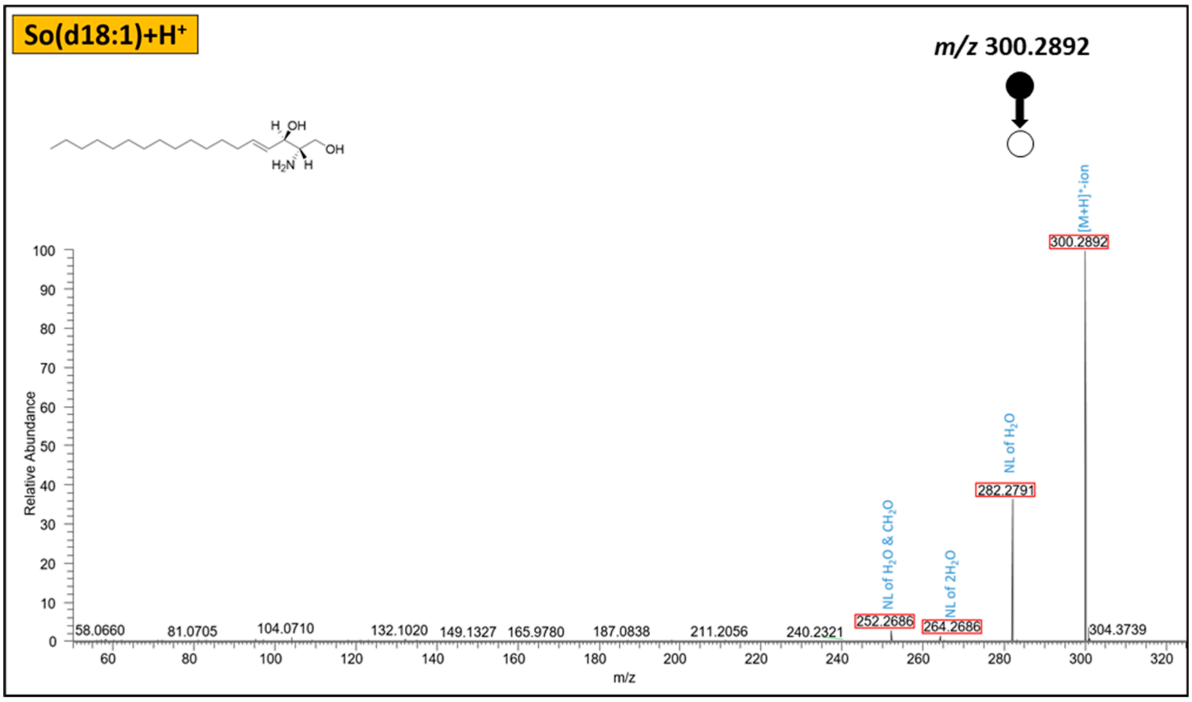

Figure S23. HCD MS/MS fragmentation pattern of So(d18:1)+H+.

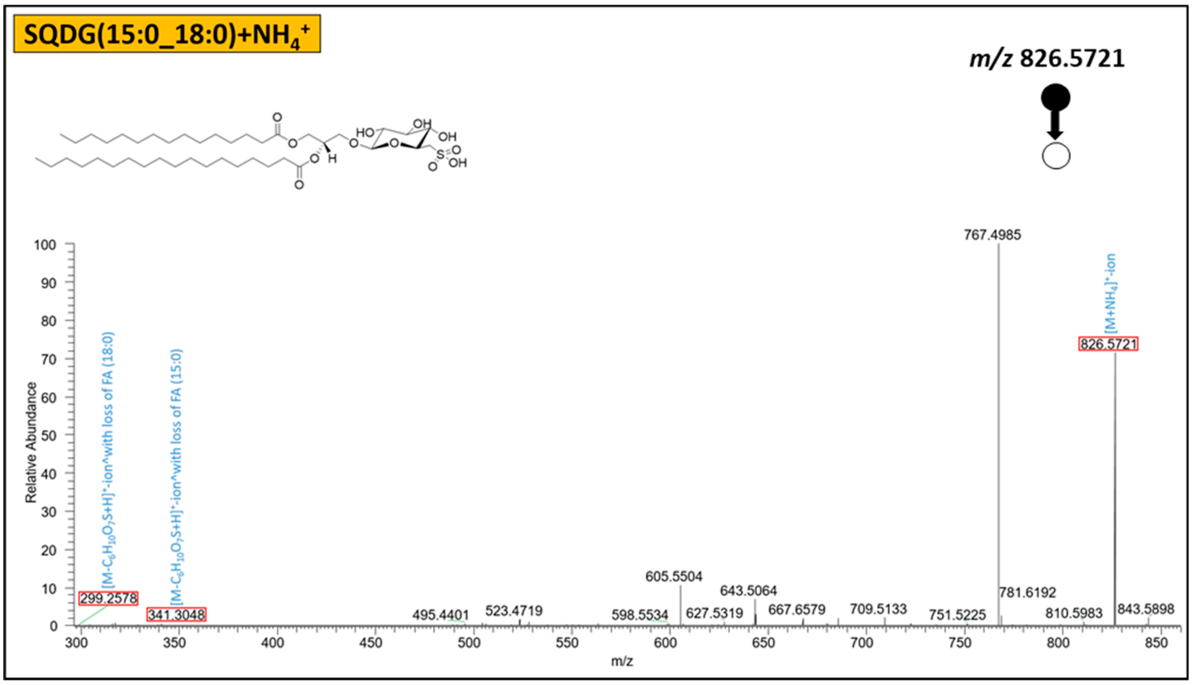

Figure S24. HCD MS/MS fragmentation pattern of SQDG(15:0_18:0)+ $NH_4^{+\cdot}$

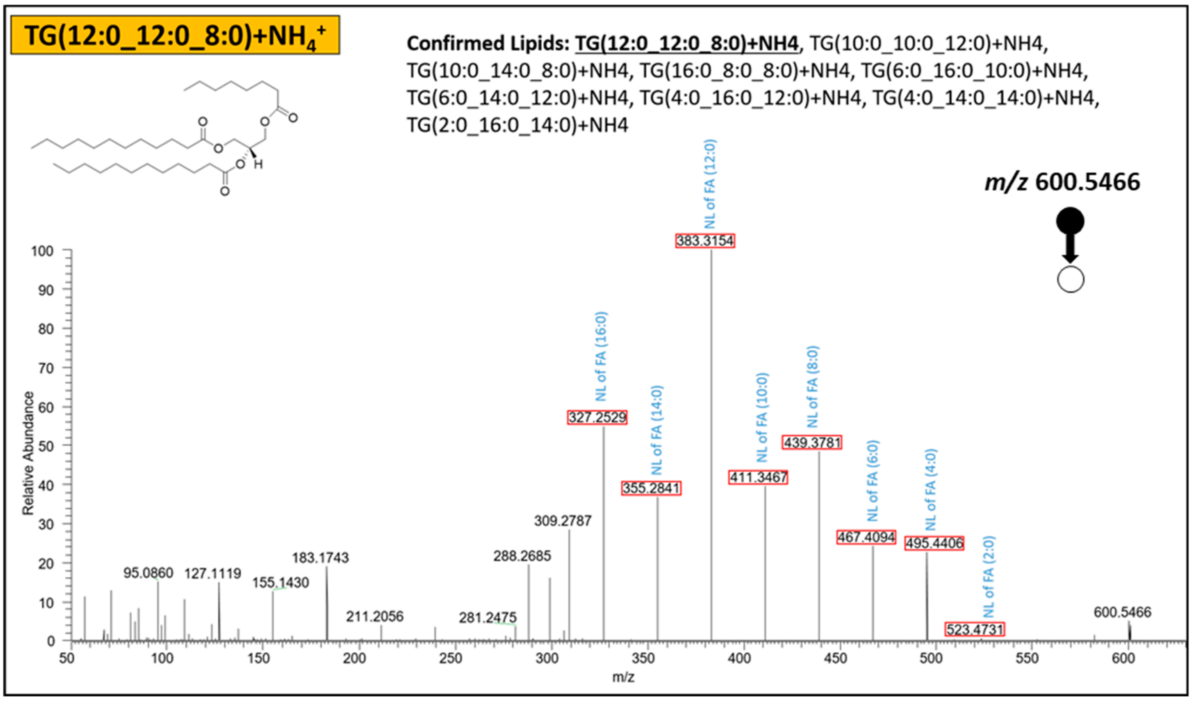

Figure S25. HCD MS/MS fragmentation pattern of TG(12:0_12:0_8:0)+ $NH_4^{+\cdot}$

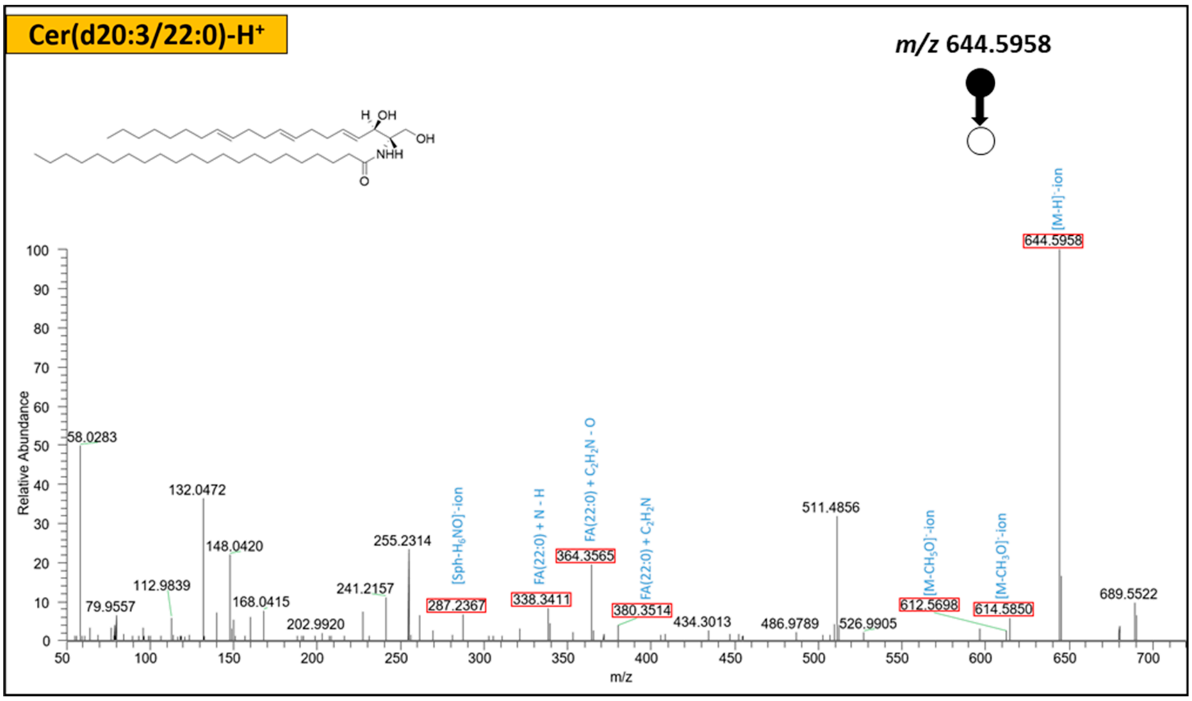

Figure S26. HCD MS/MS fragmentation pattern of Cer(d20:3/22:0)-H⁺.

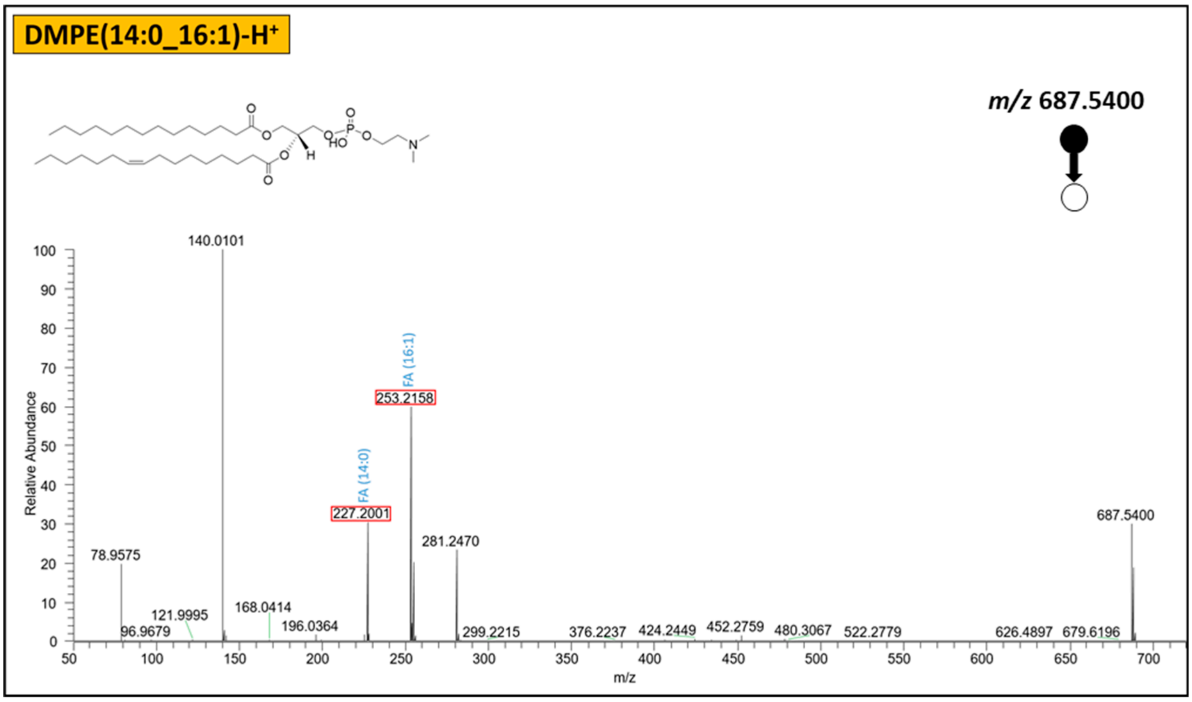

Figure S27. HCD MS/MS fragmentation pattern of DMPE(14:0_16:1)-H⁺.

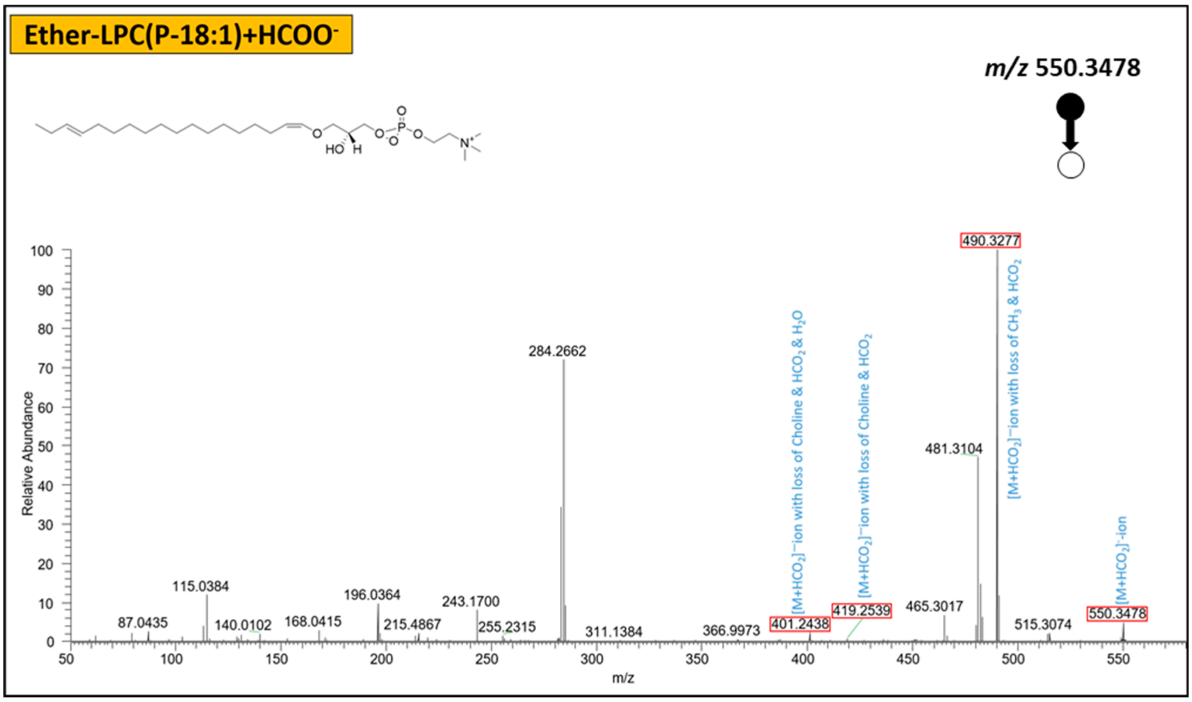

Figure S28. HCD MS/MS fragmentation pattern of ether-LPC(P-18:1)+HCOO-.

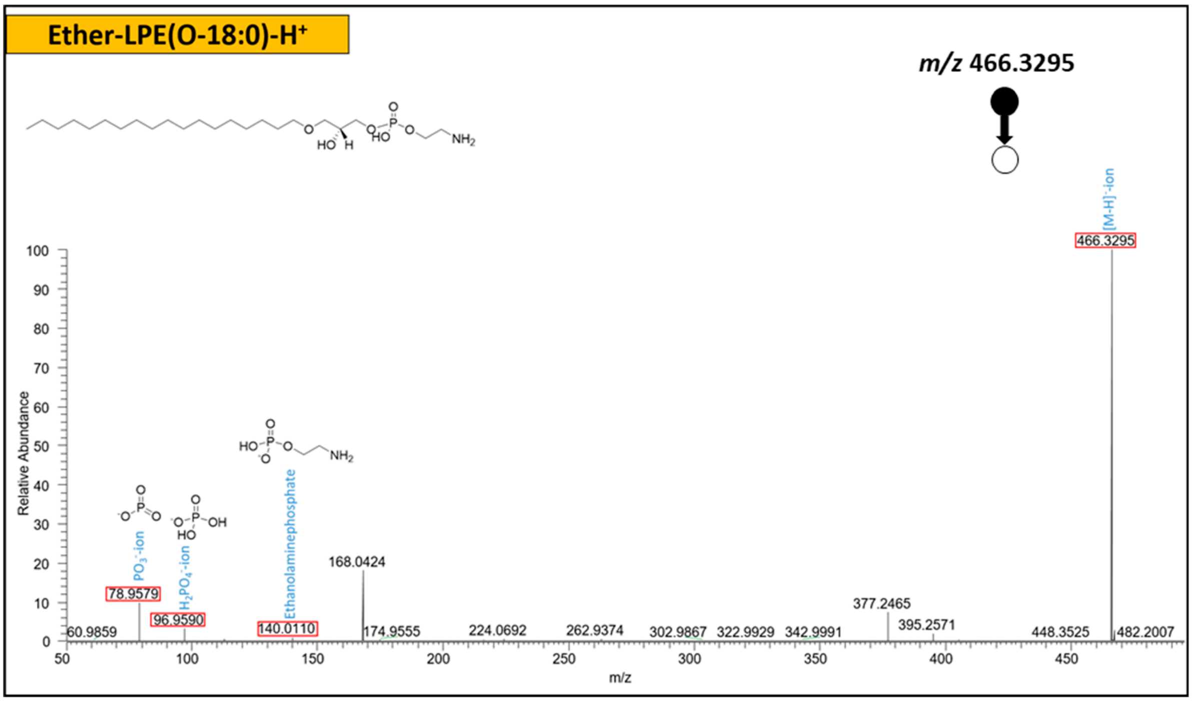

Figure S29. HCD MS/MS fragmentation pattern of ether-LPE(O-18:0)-H+.

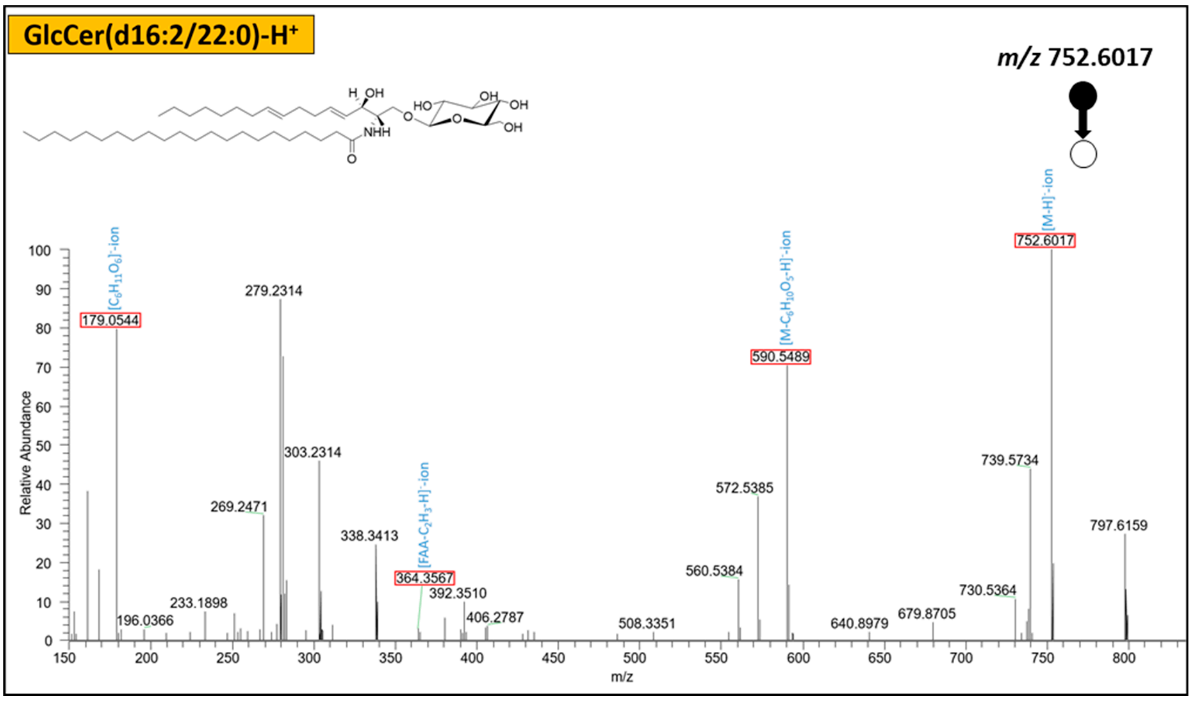

Figure S30. HCD MS/MS fragmentation pattern of GlcCer(d16:2/22:0)-H+.

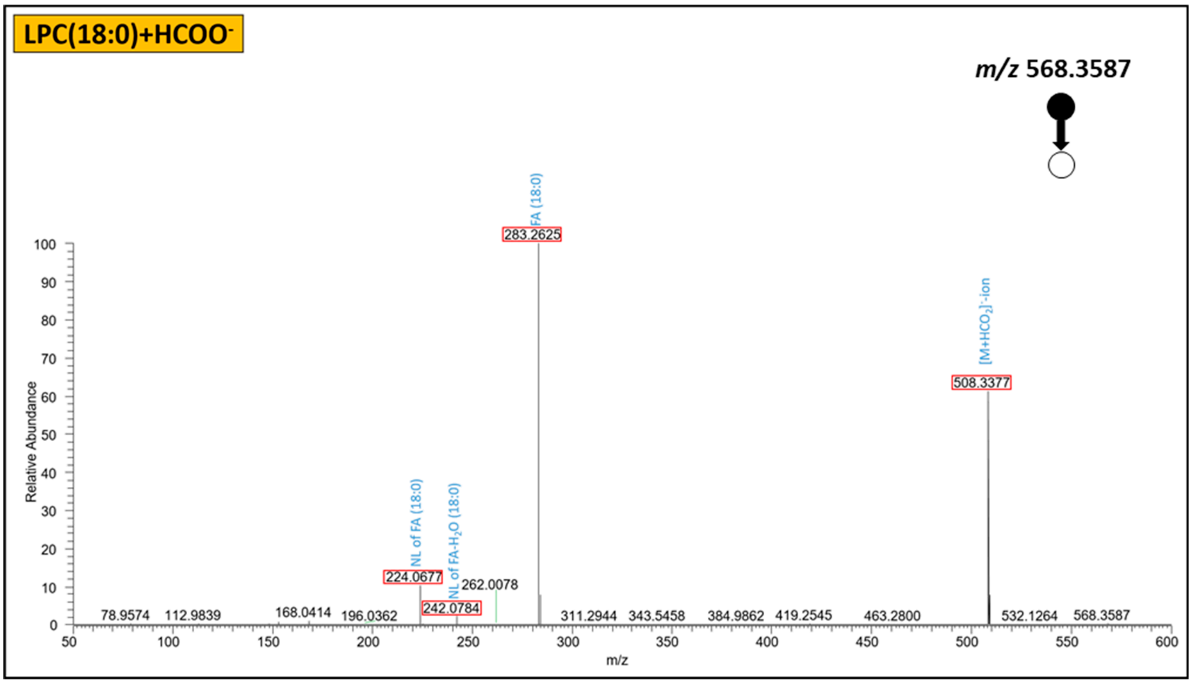

Figure S31. HCD MS/MS fragmentation pattern of LPC(18:0)+HCOO-.

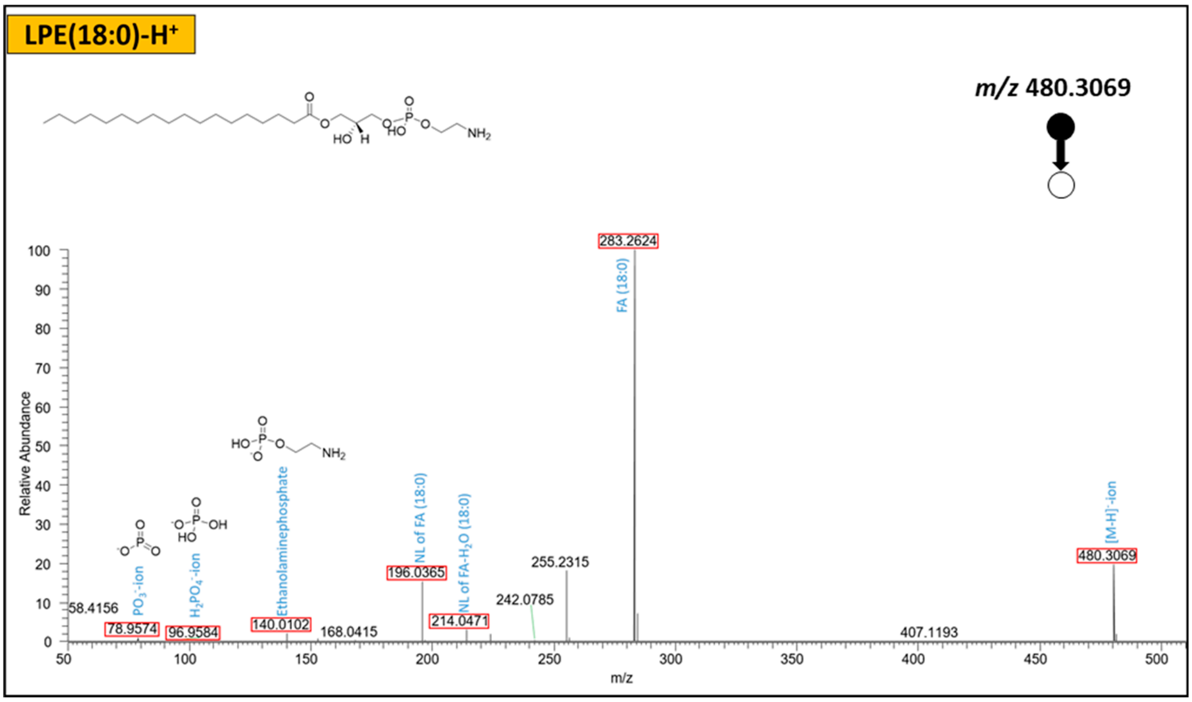

Figure S32. HCD MS/MS fragmentation pattern of LPE(18:0)-H+.

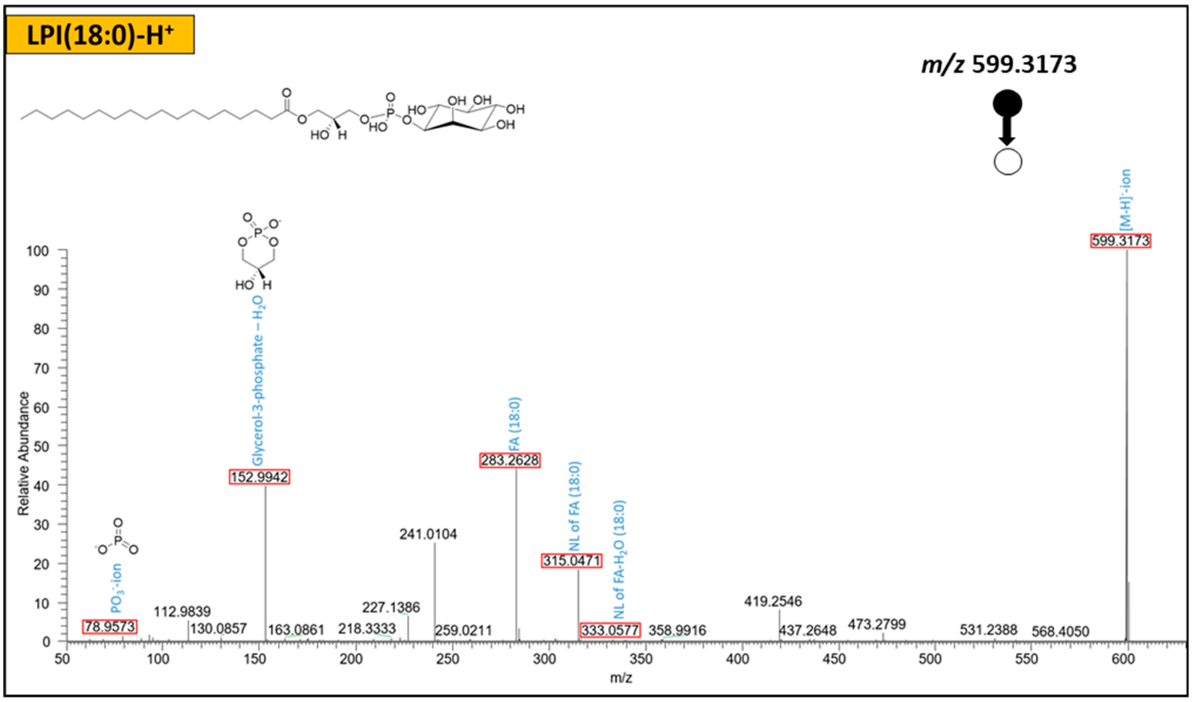

Figure S33. HCD MS/MS fragmentation pattern of LPI(18:0)-H+.

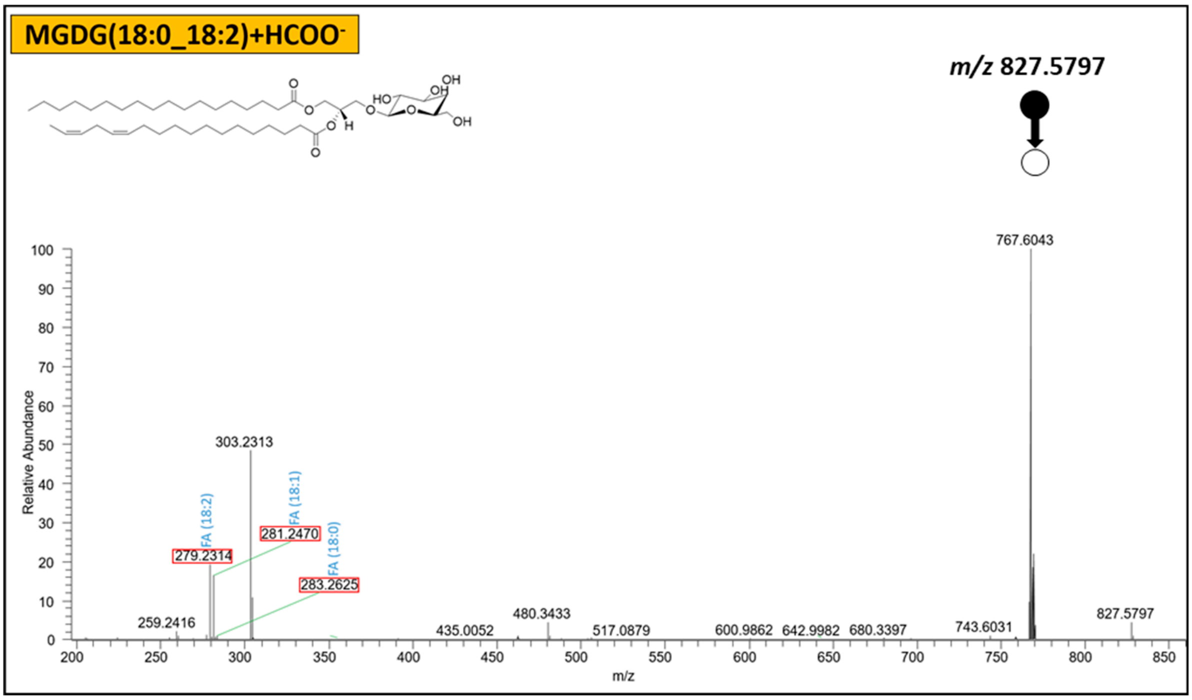

Figure S34. HCD MS/MS fragmentation pattern of MGDG(18:0_18:2)+HCOO-.

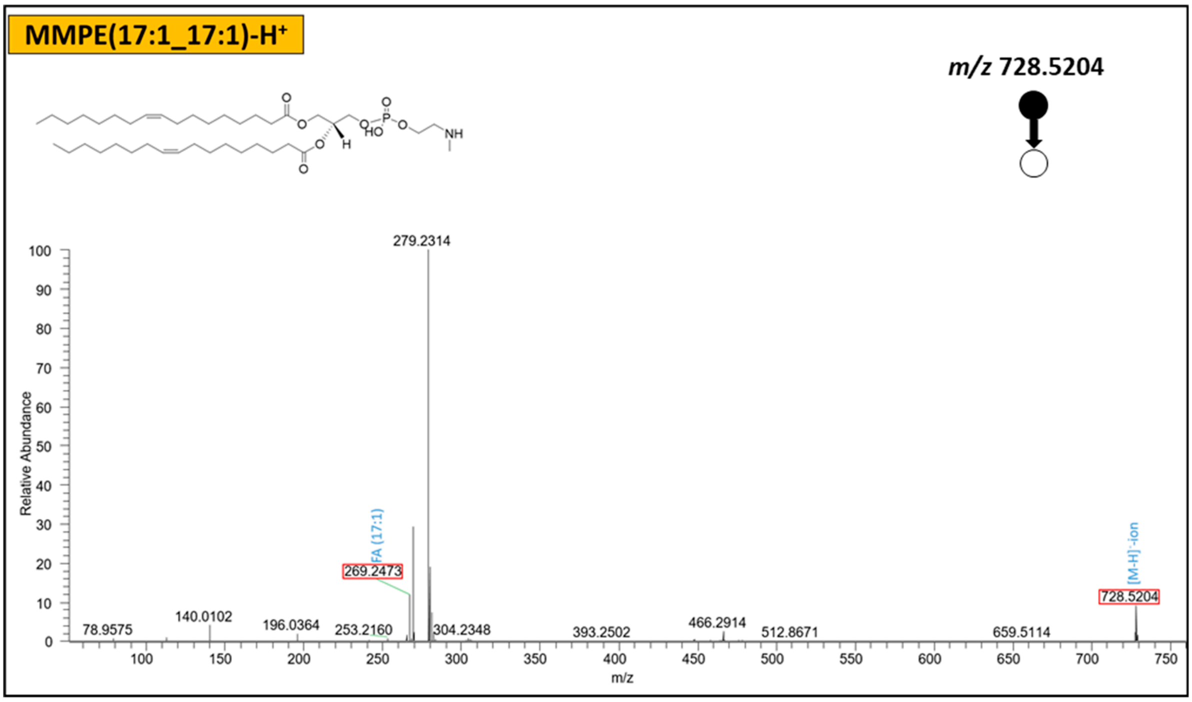

Figure S35. HCD MS/MS fragmentation pattern of MMPE(17:1_17:1)-H+.

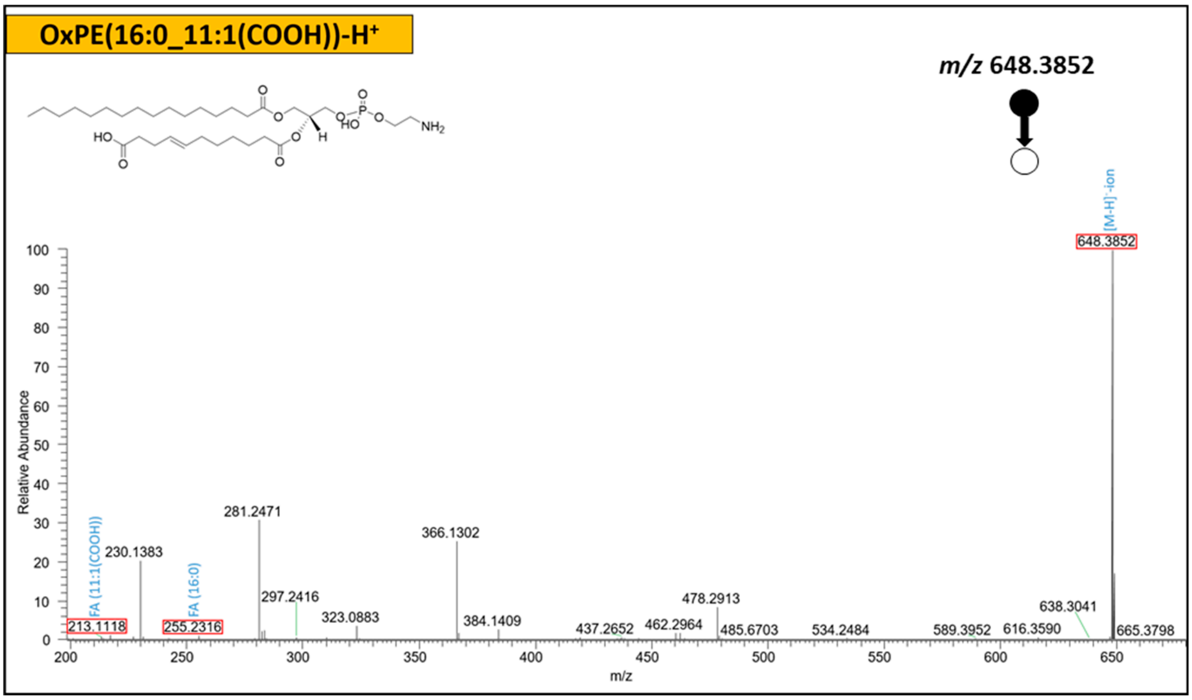

Figure S36. HCD MS/MS fragmentation pattern of OxPE(16:0_11:1(COOH))-H+.

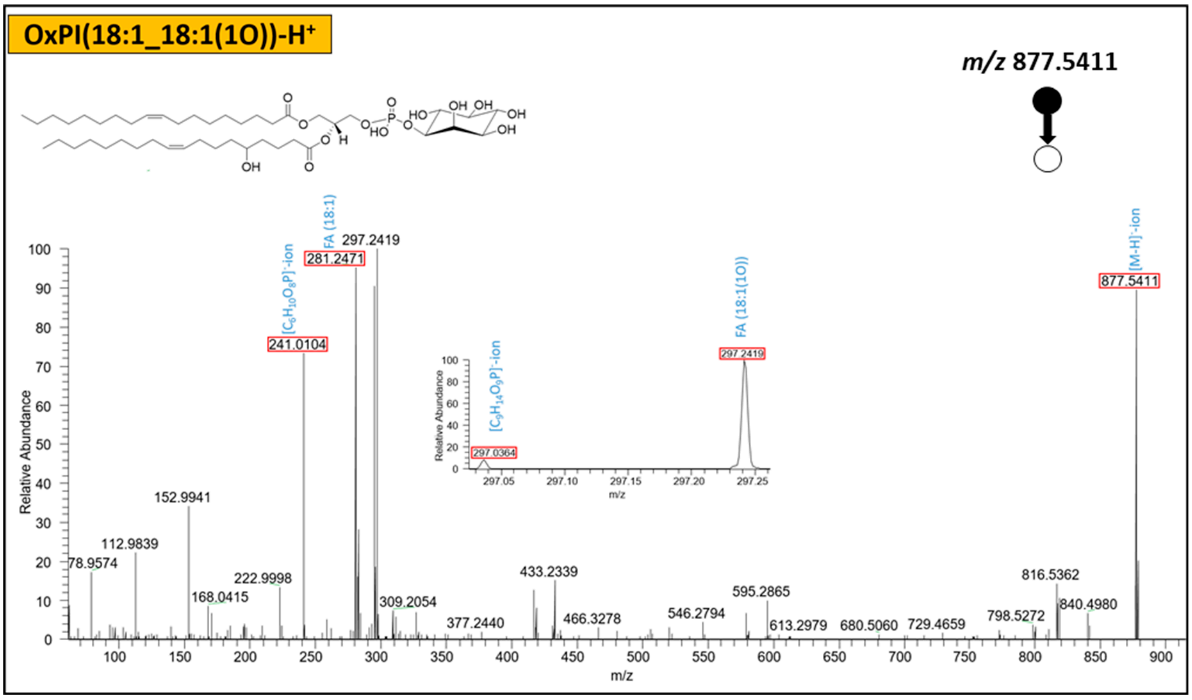

Figure S37. HCD MS/MS fragmentation pattern of OxPI(18:1_18:1(1O))-H+.

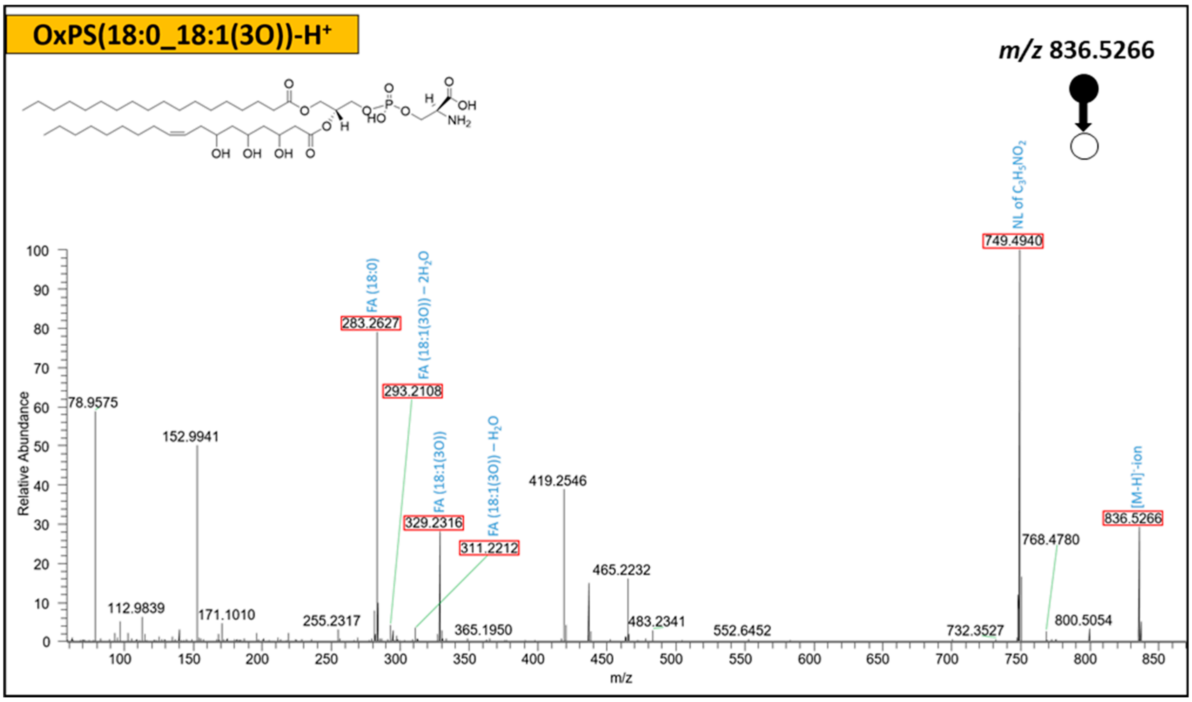

Figure S38. HCD MS/MS fragmentation pattern of OxPS(18:0_18:1(3O))-H+.

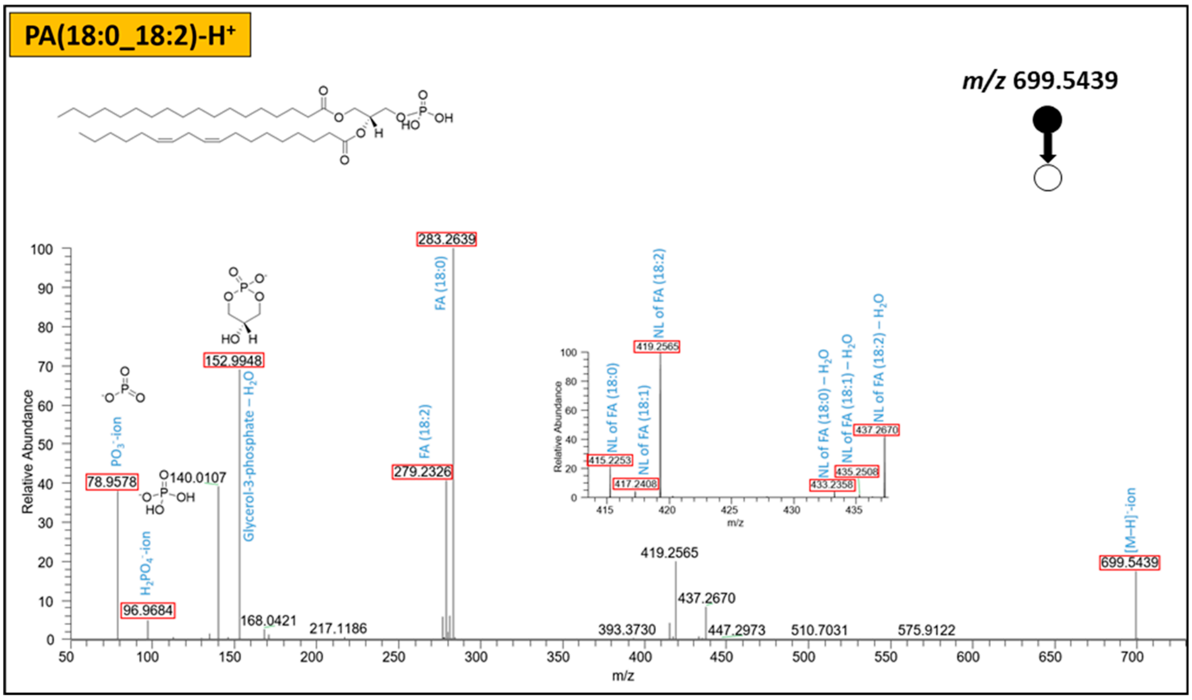

Figure S39. HCD MS/MS fragmentation pattern of PA(18:0_18:2)-H+.
